# Identification of Metabolites, Clinical Chemistry Markers and Transcripts Associated with Hepatotoxicity

**DOI:** 10.1371/journal.pone.0097249

**Published:** 2014-05-16

**Authors:** Andreas Buness, Adrian Roth, Annika Herrmann, Oliver Schmitz, Hennicke Kamp, Kristina Busch, Laura Suter

**Affiliations:** 1 F. Hoffmann-La Roche, Basel, Switzerland; 2 Metanomics GmbH, Berlin, Germany; 3 Metanomics Health GmbH, Berlin, Germany; 4 BASF SE, Ludwigshafen, Germany; University of Colorado Denver, United States of America

## Abstract

Early and accurate pre-clinical and clinical biomarkers of hepatotoxicity facilitate the drug development process and the safety monitoring in clinical studies. We selected eight known model compounds to be administered to male Wistar rats to identify biomarkers of drug induced liver injury (DILI) using transcriptomics, metabolite profiling (metabolomics) and conventional endpoints. We specifically explored early biomarkers in serum and liver tissue associated with histopathologically evident acute hepatotoxicity. A tailored data analysis strategy was implemented to better differentiate animals with no treatment-related findings in the liver from animals showing evident hepatotoxicity as assessed by histopathological analysis. From the large number of assessed parameters, our data analysis strategy allowed us to identify five metabolites in serum and five in liver tissue, 58 transcripts in liver tissue and seven clinical chemistry markers in serum that were significantly associated with acute hepatotoxicity. The identified markers comprised metabolites such as taurocholic acid and putrescine (measured as sum parameter together with agmatine), classical clinical chemistry markers like AST (aspartate aminotransferase), ALT (alanine aminotransferase), and bilirubin, as well as gene transcripts like Igfbp1 (insulin-like growth factor-binding protein 1) and Egr1 (early growth response protein 1). The response pattern of the identified biomarkers was concordant across all types of parameters and sample matrices. Our results suggest that a combination of several of these biomarkers could significantly improve the robustness and accuracy of an early diagnosis of hepatotoxicity.

## Introduction

Drug-induced liver injury (DILI) is a major concern during drug development and beyond. Pre-clinical candidate drug molecules frequently fail in development due to induced organ toxicities, often hepatotoxicity. Likewise, a common reason of withdrawal of approved drugs is toxicity. Although the incidence of drug-related hepatotoxicity is only 1 in 10000 to 100000, DILI is the leading cause of acute liver failure among patients referred to liver transplantation in the United States [1]. Despite the efforts from the last years in this field, appropriate biomarkers for monitoring liver function and to identify onset, progression and reversibility of DILI remain a major need in pharmaceutical research and in the clinic.

In this context, we define biomarkers as measurable characteristics that reflect physiological, pharmacological, or disease processes in animals or humans. They are indicative of certain biological processes or responses like adverse drug effects or treatment efficacy. Preferably, biomarkers should be measurable in body fluids, non-invasive and sensitive to be applied in clinical monitoring of potential toxicities of investigational drugs. Translational biomarkers are such for which research based on pre-clinical models can provide novel biomarkers which then may be used in clinical practice. Evolving technologies continuously improve the capabilities to measure smaller amounts of various types of biomolecules and parameters rapidly, more accurately, and in parallel in several biological matrices. These technologies include, for example, real time quantitative polymerase chain reaction (RT-PCR) and microarrays for gene expression profiling, multiplex enzyme-linked immunosorbent assay (ELISA) for the detection of proteins, and chromatography separation methods combined with mass spectroscopy for the quantification of peptides and small metabolites. These technological advancements enhance our means to systematically identify and establish biomarkers. For example in the field of nephrotoxicity, the combined analysis of gene and protein expression, metabolite concentration and in-situ hybridization and immunohistochemistry was successful in identifying putative biomarkers such as the kidney injury molecule-1 (Kim-1) that has been shown to outperform traditional markers [2], [3], [4]. Interestingly, these pre-clinical results were not only translated from the pre-clinic to the clinic and from tissue to urine, but also from the transcriptome to the proteome. In addition, the application of liquid and gas chromatography followed by mass spectrometry discovered metabolites as further potential biomarkers for the early detection of drug-induced nephrotoxicity [5]. The use of specific metabolite profiles determined using different measurement platforms for the toxicological assessment has also been published by several groups either on its own or with concomitant transcriptomic data [6], [7], [8]. In these examples, metabolite profiling is useful to identify putative biomarkers that can be readily measured in body fluids.

Given these promising published results we set out to identify biomarkers that were indicative of hepatotoxicity after acute exposure to a number of known hepatotoxicants. In order to gain granularity and mechanistic understanding, we decided to base our biomarker research not only on a single analytical technology or a single sample type, but on multiple technologies and two relevant matrices, serum and liver tissue. Therein we measured transcripts, enzymatic activities and metabolites while aiming to a more comprehensive view on the molecular changes associated with hepatotoxicity. We also aimed at combining established parameters (e.g. enzymatic activities) with less standard ones (e.g. transcriptional changes in the tissue and the small metabolite composition). In addition, appropriate phenotypic anchoring was obtained by thorough histopathological assessment of the liver tissue.

## Materials and Methods

### Study Design

Male Han Wistar rats (11–14 weeks old) were housed individually and randomly assigned to groups of 5. Animals were given water and chow *ad libitum* at all times. Regarding selection of compounds, doses and times for sample collection, studies were designed based on the known effect of the compounds, with the aim of eliciting subtle to marked hepatotoxicity. All animals were dosed either with one of following compounds (ANIT, Amineptine, Cyclosporine A, Erythromycin, Glibenclamide, Methylene Dianiline, Phalloidin, Tetracycline) or the corresponding vehicle (Table 1). Immediately preceding sacrifice, terminal blood samples for clinical chemistry investigations and determination of endogene metabolites (metabolite profiling) were collected from the retroorbital sinus. Liver samples were collected for further analysis, including gene expression measurements (transcriptomics), endogene metabolite determination (metabolite profiling) and histopathological examination. Experiments were conducted with authorization from the Swiss Federal Veterinary Office and the Association for Assessment and Accreditation of Laboratory Animal Care International.

**Table 1:** Overview of treatments.

Treatment	Dose	Route	Time	N
ANIT	30; 150 mg/kg	p.o.	24 h	10
Amineptine	0.125 mM/kg / 0.25; 0.5 mM/kg	i.p.	24 h / 3; 6; 24 h	35
Cyclosporine A	5*; 15; 30 mg/kg	i.v.	1,5*; 3*; 6 h	42
Erythromycin	734 mg/kg	i.p.	1.5; 3; 6 h	13
Glibenclamide	2.5; 25 mg/kg	i.v.	24 h	10
Methylene Dianiline	20; 100 mg/kg	p.o.	3; 6; 24 h	30
Phalloidin	0.2; 0.8 mg/kg	i.v.	3*; 6*; 24 h	30
Tetracycline	0.2 mM/kg	i.p.	3; 6 h	10
Vehicle (Control)	0 mg/kg	several	1.5; 3; 6; 24 h	100

The total number of samples is 280. Samples marked with * were not used for metabolite profiling analyses.

### Clinical Chemistry

A panel of up to 23 clinical chemistry parameters was assessed for 274 samples. The following determinations were made in serum: alanine aminotransferase (ALT), albumin (ALB), alkaline phosphatase (ALP), alpha globulins (AGLOB), aspartate aminotransferase (AST), beta globulins (BGLOB), bilirubin, bile acids (BA), calcium (Ca^2+^), chloride (Cl^–^), cholesterol, creatinine, gamma globulins (GGLOB), gamma-glutamyltransferase (GGT), glucose, inorganic phosphate (PI)), lactate dehydrogenase (LDH), potassium (K^+^), sodium (Na^+^), sorbitol dehydrogenase (SDH), total protein, triglycerides, and urea.

### Gene Expression Analysis (Transcriptomics)

A portion of liver tissue from the left medial lobe was stored at necropsy into RNALater (Ambion, Austin, TX) for RNA preservation. RNA isolation, processing and hybridization were essentially carried out as recommended by Affymetrix (www.affymetrix.com, Affymetrix, Santa Clara, CA) with minor modifications. RNA was extracted from 274 liver tissues, processed and hybridized onto Affymetrix rat genome microarray U34A. This microarray quantifies 8799 different sequences of transcripts (probe sets). The resulting gene expression intensity data were quality controlled and normalized.

### Metabolite Profiling (Metabolomics)

Two types of mass spectrometry analyses were applied: GC-MS (gas chromatography-mass spectrometry) and LC-MS/MS (liquid chromatography-MS/MS) were used for broad profiling, as described elsewhere [9]. Proteins were removed from serum samples by precipitation. Subsequently polar and non-polar fractions were separated by adding water and a mixture of ethanol and dichloromethane. Liver tissue samples were homogenized and extracted with a mixture of water, ethanol and dichloromethane. After filtration and centrifugation polar and non-polar fractions were separated. For GC-MS analysis, the non-polar fraction was treated with methanol under acidic conditions to yield the fatty acid methyl esters derived from both free fatty acids and hydrolyzed complex lipids. The non-polar and polar fractions were further derivatized with O-methyl-hydroxylamine hydrochloride and pyridine to convert oxo-groups to O-methyl-oximes and subsequently with a silylating agent before analysis [10]. For LC-MS/MS analysis, both fractions were reconstituted in appropriate solvent mixtures. High-performance liquid chromatography (HPLC) was performed by gradient elution using methanol/water/formic acid on reversed phase separation columns. Mass spectrometric detection technology was applied which allows target and high sensitivity MRM (multiple reaction monitoring) profiling in parallel to a full screen analysis (patent application: WO2003073464). For GC-MS and LC-MS/MS profiling, data were normalized to the median of reference samples which were derived from a pool formed from aliquots of all samples to account for inter- and intra-instrumental variation. Metabolite profiling of liver and serum samples was performed by Metanomics Health (Berlin). In serum, we identified 200 metabolites at concentration levels above the limit of quantification allowing semi-quantitative (SQ) analysis. In liver tissue, 545 SQ metabolites were measured.

### Histopathology

Representative liver samples were fixed in 10% neutral buffered formalin. One additional liver sample from top half of the left lateral lobe was placed in Carnoy fixative. All samples were processed using routine procedures and embedded in Paraplast. Tissue sections approximately 2–3 microns were cut and stained as follows for histopathological evaluation: hematoxylin-eosin, periodic acid-Schiff for glycogen, and Oil Red-O for lipid. Hematoxylin-eosin and Oil Red O stained liver slides were evaluated histopathologically. The histopathological assessment was the basis for the classification of the samples into positive and negative for liver toxicity. Any of following findings was considered to indicate liver toxicity and drove the classification of the samples: apoptosis or single cell necrosis, hemorrhage, hepatocellular cytoplasmic vacuolation different from fat droplets (Oil Red O slides), necrosis and inflammation. All other observations noted in the histology evaluation like glycogen content were not considered to reflect hepatotoxicity and were thus not used for the classification.

### Data Analysis

We transformed the measurements of all parameters (gene expression, metabolite profiling and clinical chemistry) to the log2-scale. The mean of the time matched control group (vehicle treatment) was subtracted from each data point. Hence, the control groups were centered at zero. The histopathological classification of the samples was refined and the samples were divided into three classes: negative, positive and increased. The negative class comprised all samples which neither showed any relevant histopathological alteration nor any substantial changes in ALT or AST measurements. None of the measurements of the control samples, i.e. vehicle treated samples, deviated from the average of their group and thus all control samples were classified negative. The positive class encompassed all samples with histopathological findings as described above, irrespective of ALT and AST changes. The third class, termed ‘increased’, contained all samples which were classified as negative by means of the histopathological descriptors mentioned above, but showed at least a two-fold increase in either ALT or AST compared to the mean of the corresponding control group. Following this partitioning a two-tailed t-test with equal variance assumption was applied to identify parameters with significant differences between the positive and the negative class. To account for multiple testing and to reduce and control the number of false positive parameters, we calculated the false discovery rate (FDR, Benjamini and Hochberg method) for each type of measurement separately. All calculations were performed with the statistic software R, Bioconductor and in-house tools (www.r-project.org, www.bioconductor.org). The interpretation of obtained results primarily focused on parameters which were significantly deregulated (FDR value <0.05) and in addition exhibited fold-changes of at least twofold (FC >2).

## Results

Eight independent animal studies performed in male Wistar rats were used to identify early biomarkers associated with histopathologically evident hepatotoxicity. The inclusion of measurements within 24 hours of dosing allowed us to monitor potential changes in biomarker concentration before toxic injuries were detectable at the microscopic level. Details on all studies, treatments and time points are summarized in Table 1.

Histopathologically positive samples were found in animals treated with ANIT, Erythromycin, Methylene Dianiline or Phalloidin (Table 2). ANIT, Erythromycin, Methylene Dianiline and Phalloidin caused hepatocellular damage characterized by single cell necrosis/apoptosis, inflammation and vacuolation. Phalloidin in addition caused hepatic hemorrhage. The results, with the observed incidence are summarized in Table 2. The other tested compounds did not cause histopathological changes in the liver. Clinical chemistry results showed increase in liver parameters in the animals showing histopathological findings in the livers, but also in treated animals without microscopic indications of liver damage (Figures S1 and S2).

**Table 2:** Histopathological findings in the liver (positive samples/total of samples).

Histopathologic findings	Treatment, dose level, time points									
	ANIT		Erythromycin		Methylene Dianiline			Phalloidin		
	30 mg/kg 24 h	150 mg/kg 24 h	734 mg/kg 3h	734 mg/kg 6h	100 mg/kg 3h	100 mg/kg 6h	100 mg/kg 24h	0.8 mg/kg 3h	0.8 mg/kg 6h	0.8 mg/kg 24h
**Apoptosis/single cell necrosis**	–	3/5	1/5	2/5	4/5	5/5	5/5	5/5	5/5	5/5
**Inflammation**	1/5	2/5	–	–	–	5/5	5/5	–	–	–
**Hepatocellular vacuolation**	–	–	1/5	1/5	–	–	–	–	–	–
**Hemorrhage**	–	–	–	–	–	–	–	5/5	5/5	5/5
**Necrosis**	–	–	–	–	–	–	–		5/5	4/5

Compounds not listed did not cause any relevant changes in the liver.

Based on the histopathological findings and on the ALT and AST measurements, individual animals were assigned to one of the three classes defined in materials and methods, e.g. negative, positive and increased (Table 3). The statistical analysis aimed at identifying differences while enhancing the contrast between the negative and positive class. For this purpose, a two-tailed t-test was applied to determine parameters which were significantly deregulated between the negative class (no histopathological finding in the liver) and the positive class (histopathological evident hepatotoxicity). Samples with increased ALT or AST but without concomitant histopathological manifestation (classified as “increased”) were left out in this step. This approach was motivated by the following considerations. Firstly, we wanted to base our analysis on confirmed toxicities, i.e. the positive class, rather than on expectations on the effects of the treatment. Therefore, we only classified as positive samples that had a clear phenotypic anchoring (histopathology) [11]. Secondly, we expected that molecular responses indicative of hepatotoxicity would arise prior to the morphological evidence (e.g. in animals without histopathological findings that were treated with known toxicants). Hence, a direct comparison based solely on histopathologically detected hepatotoxicity (negative versus the positive class) may bias the results in the direction of late stages molecular changes since early molecular alterations are presumably already present before histopathological correlate. Thirdly, ALT and AST are well known, sensitive serum markers of hepatotoxicity [12]. Thus, while aiming at an enhanced contrast between the positive and negative class we decided to remove samples which potentially represent early stages of hepatotoxicity from the negative class. This was achieved by leaving out all samples with increased ALT or AST. An overview of the sample numbers for each parameter type and each class is given in Table 4. As indicated in Table 2 fewer treatment groups were used for metabolite profiling, particularly reducing the larger sample numbers of the negative class. For the clinical chemistry parameter gamma-glutamyltransferase (GGT) substantially less sample measurements were available (see Figure S1 for the details of all clinical chemistry parameters).

**Table 3:** Subdivision of samples into three classes split by treatment.

Treatment	Negative	Increased	Positive	N (total)
ANIT	6	0	4	10
Amineptine	15	20	0	35
Cyclosporine A	41	1	0	42
Erythromycin	2	8	3	13
Glibenclamide	10	0	0	10
Methylene Dianiline	16	0	14	30
Phalloidin	15	1	14	30
Tetracycline	1	9	0	10
Vehicle (Control)	100	0	0	100
**Sum**	206	39	35	280

**Table 4:** Overview of samples per class and parameter type (highest numbers are shown).

Parameter	Negative	Increased	Positive	N (total)
Clinical Chemistry	202	39	30	271
Metabolites in Liver	131	37	25	193
Metabolites in Serum	130	31	20	181
Transcripts in Liver	202	39	33	274

As a first step we quantified the sensitivity and specificity of the ALT and AST serum measurements in detecting histopathologically evident hepatotoxicity based on our criteria. Serum ALT and AST data were available for 30 out of the 35 samples in the positive class. From these samples, 16 showed a two-fold increase in at least one enzyme and 14 did not exceed this cut-off value. In addition, the class termed ‘increased’ contained further 39 samples above the two-fold cut-off in either ALT or AST. This corresponds to a sensitivity of 53% and specificity of 84% in our data set (200 true negative samples).

Regarding other putative biomarkers, our tailored approach identified 75 parameters in serum and liver tissue which were found to be significantly associated with acute hepatotoxicity (FDR smaller than 0.05) and which were changed at a fold-change larger than two. They are listed in separate tables for each type of parameters and consist of 7 clinical chemistry markers in serum, 5 metabolites in liver tissue, 5 metabolites in serum and 58 transcripts, mapping to 45 distinct genes, in liver tissue (Tables 5–8, respectively, Figures S3–S6 and Tables S1–S3). The multiple occurrences of some probe sets in the result list underlined the reproducibility of their measurement, e.g. we identified four times Egr1 (early growth response protein 1) and three times Btg2 (B-cell translocation gene 2) and Jun (*jun proto-oncogene*).

**Table 5:** Significantly changed (FDR <0.05) clinical chemistry markers in serum exhibiting absolute fold changes (FC) >2.

Name	FDR	logFC	FC
Bilirubin	0.00	3.18	9.06
BA	0.00	2.94	7.70
ALT	0.00	2.75	6.72
GGT	0.00	2.61	6.09
AST	0.00	2.38	5.19
SDH	0.00	2.12	4.34
LDH	0.00	1.74	3.35

**Table 6:** Significantly changed (FDR <0.05) metabolites in liver tissue exhibiting absolute fold changes (FC) >2.

Name	FDR	logFC	FC
Putrescine (additional: agmatine)	0.00	2.02	4.04
Glycochenodeoxycholic Acid	0.01	−1.34	0.39
Taurocholic Acid	0.00	1.15	2.21
Unknown	0.00	1.12	2.17
Maltotriose	0.03	−1.06	0.48

**Table 7:** Significantly changed (FDR <0.05) metabolites in serum exhibiting absolute fold changes (FC) >2.

Name	FDR	logFC	FC
Taurocholic Acid	0.00	5.35	40.77
Glycocholic Acid	0.00	3.42	10.72
Taurochenodeoxycholic Acid	0.00	3.21	9.23
Glucuronic Acid	0.00	1.39	2.61
Arginine	0.00	−1.25	0.42

**Table 8:** Top 10 ranking significantly changed (FDR <0.05) transcripts in liver tissue exhibiting absolute fold changes (FC) >2.

Identifier	Name	FDR	logFC	FC
M58634_at	Igfbp1	0.00	2.94	7.67
AF023087_s_at	Egr1	0.00	2.73	6.65
U75397UTR#1_s_at	Egr1	0.00	2.65	6.28
M18416_at	Egr1	0.00	2.63	6.18
D38066exon_s_at	Ugt1a2	0.00	2.23	4.68
M60921_g_at	Btg2	0.00	2.2	4.59
rc_AI175959_at	Jun	0.00	2.05	4.14
rc_AA944156_s_at	Btg2	0.00	2.04	4.1
rc_AA900505_at	Rhob	0.00	1.97	3.91
M63282_at	Atf3	0.00	1.86	3.63

**Figure 1:**
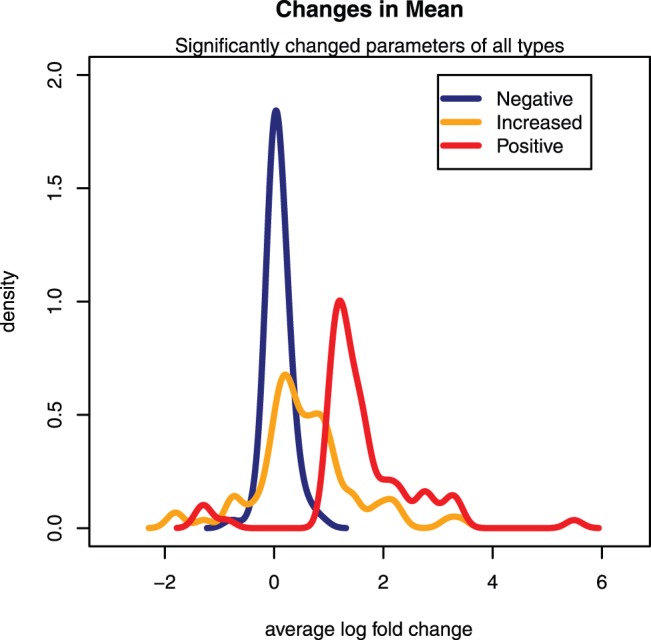
Changes in Mean. In total 75 parameters were found to be significantly changed between histopathologically negative and positive scored samples (false discovery rate <0.05) and exhibiting fold-changes >2 or <0.5. The average fold-changes of all 75 parameters were calculated with respect to the matching control group and their smoothed distribution is shown for each class. Control group means centered at zero are not shown. Samples which are scored negative and show increased ALT or AST were omitted in the statistical comparison and are shown as separate line in yellow. Their intermediate distribution reassures the validity of this approach and the relevance of the identified parameters.

The magnitude of deregulation varied across these 75 parameters. The distribution is visualized in Figure 1. The average fold-change when compared to the matching control group is shown as a colored line for each class separately. As a consequence of the selection criteria, the changes of the mean of the class of the negative samples for the 75 parameters are centered on zero (no change) when compared to the matching control group. Likewise, as a consequence of the selection criteria the changes in mean of the positive class discriminate well from those of the negative class for the 75 identified parameters. The number of parameters with an increased level in the positive class by far exceeded those which were down-regulated. Most interestingly to note is that, the distribution of the class termed ‘increased’ lies between the negative and positive classes, thus reassuring us of the validity of our tailored approach. Hence, samples with no evident histopathological finding, but two-fold increase in either ALT or AST, clearly show further alterations on the molecular level in serum and liver tissue.

**Figure 2:**
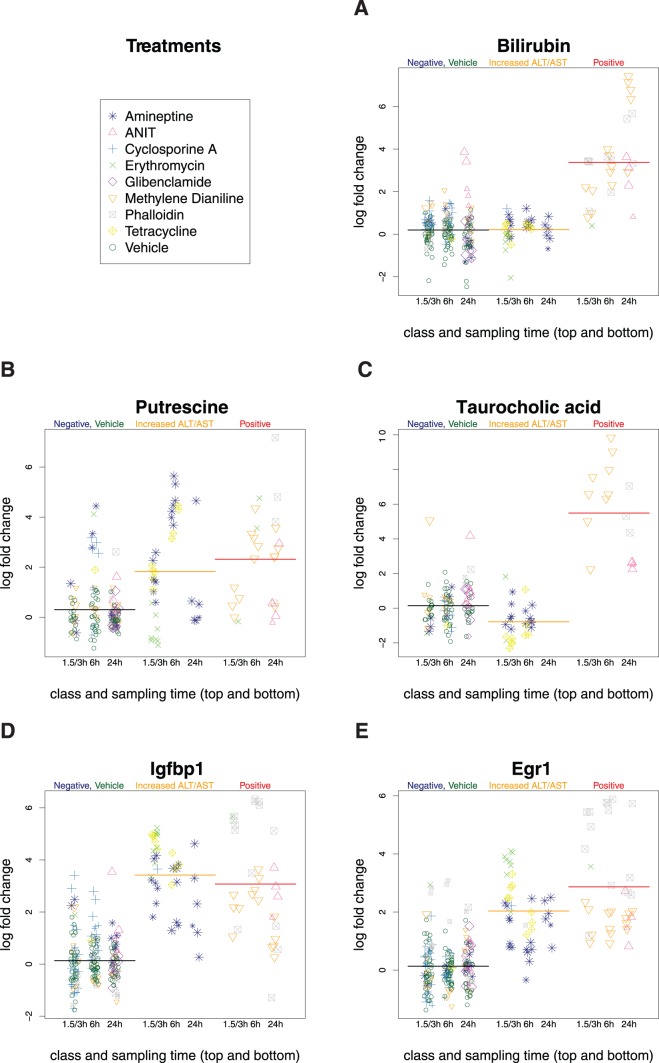
Top Markers. All measurements are visualized in detail for the 1–2 best ranked markers for each type of parameter (subfigure A: clinical chemistry in serum, B: metabolite in liver tissue, C: metabolite in serum, D/E: transcript in liver tissue). The fold-change of each sample is calculated with respect to the matching control group (vehicle treatment). The mean of each class is shown as horizontal line (black: negative including controls, yellow: increased in ALT or AST, red: positive). The size of the treatment symbols increase with dose. The time points are resolved by aligning the symbols in columns.

A detailed graphical visualization of the measurements is given for the best ranked parameters in Figure 2 and all other parameters in Figures S3–S6. The parameters were ranked by significance level (p-value) followed by amplitude of change (fold-change), see Tables 5–8 and Tables S1–S3. The best ranked parameters for each type of measurement were selected, i.e. bilirubin for clinical chemistry, putrescine and taurocholic acid from the metabolite profiling measurements and Igfbp1 (insulin-like growth factor-binding protein 1) and Egr1 from the gene expression data. The graphical representation depicts the variation from the controls since all measurements were calibrated including the vehicle treated control groups which were centered at zero. These variations of the controls tend to be within the two-fold range, but some extreme cases show changes up to four-fold. This outcome appears to be irrespective of the type of measurement and sample type, i.e. serum or tissue. Moreover, this outcome appears to be unrelated to the matrix (serum or tissue) or the technology of measurement. In addition, the data shows a dynamic response in time, as seen in the graphical representation. For example, bilirubin levels increase in a time-dependent manner in animals treated with Methylene Dianiline and histopathologically positive scored samples. In addition to these detailed graphs, a combined view of all 75 parameters is presented as a heatmap-like representation (Figure S2).

A closer inspection of the detailed graphical visualization revealed two individuals that clearly deviated from the rest of the animals in the negative class (Figure 2). These two animals were treated with a high dose of ANIT or Methylene Dianiline, respectively. In particular, they showed increases in taurocholic acid of approximately 16- and 32-fold at 24 h and 3 h, respectively. Moreover they also showed increase in bilirubin (16x, 2x), in Igfbp (11x, 4x), in Egr1 (2x, 4x) and in putrescine (3x, none). Given the consistent deviation of these individual animals from the negative class, we decided to re-evaluate the histopathological classification. In the case of the animals at the early time point of 3h treated with Methylene Dianiline we could not detect any histopathological changes upon re-evaluation. However, re-assessment of the liver tissue of the rat treated with ANIT showed single cell necrosis of grade one, which had been overlooked in the first evaluation. These observations suggest the relevance of our results, indicating that objective quantitative measurements can at times outperform histopathological evaluation and suggesting a higher sensitivity of analyte measurements compared to morphological assessment.

Taking into account treatment effects with fold changes <2, a large number of additional metabolites exhibiting significant changes could be identified both in serum (n = 97) and liver (n = 144). The total number of significantly changed metabolites with known structure ID was 63 and 85 for liver and serum, respectively (Tables S1 and S2; metabolites are sorted according to their ontology classes). Metabolite concentration changes in serum were largely observed for amino acids and related (including tryptophan, metabolites of the tryptophan metabolism and metabolites of the creatine metabolism) as well as lipids, e.g. several (lyso)phospho- and sphingolipids and other complex lipids (e.g. triacylglycerols) and fatty acids derived from complex lipids. In addition to the already discussed bile acids, increases were observed in particular for complex lipids and fatty acids that are components of cell membranes. The metabolite classes amino acids (and related) as well as lipids (again different lipid classes) exhibited major changes also in liver (Table S1). It is noteworthy to mention that particularly for amino acids and related we observed quite some overlap of metabolites being significantly changed in the same direction both in serum and liver (see Tables S1 and S2, respectively). This applied to glutamate, alanine and trans-4-Hydroxyproline (decreased by up to 27%) as well as leucine, valine, creatine, creatinine and ornithine (increased by up to 70%). In contrast, lysine and arginine are examples exhibiting opposite changes in both matrices, the latter one being decreased in serum by 58% and increased in liver by 30%).

The observed gene expression changes were summarized with regard to gene ontology (GO) classes. To this end, all measured transcripts were annotated and mapped to GO classes. We focused on the most significant 500 deregulated transcripts irrespective their fold change (p-value <10^–7^). Those GO classes were selected which were enriched in our list of 500 deregulated transcripts by means of the Fisher exact test. The following 9 GO classes were found overrepresented in our data set with a p-value <10^–5^: response to stimulus, external stimulus, chemical stimulus, stress, wounding, hormone stimulus, organic substance and endogenous stimulus; and regulation of angiogenesis.

## Discussion

The aim of this investigation was to identify putative early biomarkers for hepatotoxicity. With this purpose in mind, we utilized clinical chemistry parameters, metabolite profiling (LC-MS/MS and GC-MS) and gene expression in serum and/or liver. We identified parameters which were associated with histopathological findings in the rat liver. Most of these parameters were elevated with an overall concordant pattern across the different types of measurements and matrices (e.g. tissue transcripts and serum metabolites). Here, we present the results of our study which offer a repertoire of potential biomarkers to assess and monitor DILI.

Our approach compares samples with treatment-related histopathological findings in the liver (called ‘positive’) with those which do not show any substantial alterations neither histopathologically nor with regards to the their serum levels of ALT or AST (called ‘negative’). The approach implicitly aims to identify universal biomarkers, i.e. biomarkers which are indicative for DILI irrespective of the type of the toxic injury or its underlying molecular mechanism. However, a single, universal marker associated with any kind of hepatotoxicity may not exist, since early toxic events may differ among different mechanisms of toxicity. A biomarker discovery strategy that initially stratifies hepatotoxicity based on the type of finding or mechanism could be used to identify mechanism-specific markers [13]. Here, we did not attempt to do so, but rather focused on histopathologically positive samples, irrespective of the mode of action. Biological understanding of the roles of the detected putative biomarkers provided additional evidence of the true (rather than spurious) association between the detected markers and the underlying cellular processes.

Thus, we assessed the set of parameters identified as significantly changed upon toxic insult to the liver considering their biological function. The parameters included in the clinical chemistry panel and that were elevated in the positive samples are known to be associated with hepatotoxicity (Table 5 and Figure S3). For example, Hy’s law, defined as elevations of circulating transaminases (ALT or AST) and bilirubin has become the standard of practice to determine the severity of liver damage in the clinic. This combination of parameters, however, will only indicate serious cases of liver damage in patients [14]. Gamma-glutamyltransferase (GGT) is localized to bile ducts, so that elevations in circulating levels are indicative of hepatobiliary damage. Sorbitol dehydrogenase (SDH) elevations were found to be specific for liver damage, but much less sensitive than the transaminases. Lactate dehydrogenase (LDH) is considered a general marker of tissue damage, although certain isoforms (LDH-4 and LDH-5) are more specific to liver or muscle disease.

In association with hepatotoxicity, we detected increased bile acids in serum (total bile acids, glycocholic and taurochenodeoxycholic acids) and in liver tissue either by clinical chemistry or by metabolite profiling. The fact that this metabolite family can be detected in both matrices, irrespective of the analytical method confirms that bile acids are robust and informative markers of liver damage. These organic anions are synthesized in hepatocytes from cholesterol, conjugated, and excreted into the canaliculus [15]. Measurement of serum bile acid concentrations is a more specific indicator of functional hepatic excretory capacity than is serum bilirubin [16]. Our data suggest that differential determination of total and/or specific bile acids may add information on subtle excretory abnormalities and improve sensitivity by employing of more accurate analytical methods such as GC/MS or LC/MS. Further indication of detoxification processes triggered in the liver by the treatments is the increase in circulating glucuronic acid.

Metabolite profiling has proven high reliability and reproducibility in the past. In many cases rather small yet physiologically meaningful changes observed in a highly controlled matrix like serum or plasma could be assigned to specific toxicological mode of actions. In particular, it has been shown that plasma metabolome changes, e.g. for amino acids, fatty acids and complex lipids, below a fold change of 2 can be indicative for liver toxicity [17], [18], [9]. Furthermore, increased values of creatinine in rat urine have been reported to be related to treatment with the hepatotoxicant hydrazine [19] and increases of urinary bile acids have been associated with ANIT treatment [20]. These results are in line with highly significant metabolome changes observed in this study, such as increases of creatinine and creatine levels both in liver and in serum. Recently it was reported that changes in circulating bile acids as well as amino acids are associated with acetaminophen-induced hepatotoxicity in rats [21]. In particular, elevated blood bile acids have been observed, with increases in cholic acid, deoxycholic acid and glycocholic acid being statistically significant. Strong correlations with the hepatotoxic outcome of acetaminophen were also observed for e.g. arginine and derivatives [21]. In line with this observation, in the current study serum arginine levels decreased while serum ornithine levels increased (Table S2). The corresponding urea cycle enzyme arginase I, catalyzing the conversion of arginine to ornithine and urea, exhibited a significant increase in transcript levels by ∼20% in liver tissue as well (not shown). Remarkably, the metabolites arginine, ornithine, citrulline and urea exhibited significant increases in liver (Table S1), confirming deregulation of the urea cycle as seen in the transcriptomics analysis in the current study: in addition to the increase of arginase I, an increase of argininosuccinate dehydrogenase by ∼30% and a decrease of ornithine carbamoyltransferase by ∼13% was observed (not shown).

Serum metabolite profiling revealed changes in tryptophan metabolism: tryptophan decrease by 30% was accompanied by decreases of indole-3-propionic acid and indole-3-lactic acid by 28% and 13%, respectively, whereas kynurenic acid was considerably increased by a factor of ∼1.5 (Table S2). Remarkably, expression of tryptophan-2,3-dioxygenase (TDO), catalyzing the first and rate-limiting step of tryptophan degradation in liver, thereby producing formylkynurenine, was significantly increased by 15% whereas expression levels of kynurenine 3-monooxygenase (converting kynurenine to 3-hydroxykynurenine) and kynureninase (catalyzing the following enzymatic step converting 3-hydroxykynurenine further to 3-hydroxyanthranilic acid) were decreased by 30% and 26%, respectively (not shown). This is in line with the observed increase of kynurenic acid indicating that tryptophan degradation to NAD was shifted towards synthesis of kynurenic acid.

The most prominent candidate marker identified based on metabolite profiling is putrescine (measured as sum parameter together with agmatine), a simple polyamine, which showed an increase of more than four-fold (Table 6, Figure 2 and Figure S4). Although our analytical methods, based on full tissue homogenates, do not allow for cellular localization of the changes, it is interesting to note that the increase of putrescine may be linked to activation of macrophages. The clearance of debris of necrotic cells containing danger signals makes macrophages one of the primary sensors of danger present in the host. Alternatively activated macrophages primary role appears to be related to wound healing, decreased inflammation and tissue repair. These macrophages contribute to the production of polyamines and collagen. Remarkably, major components of collagen, i.e. proline, trans-4-hydroxyproline, glycine and others, are decreased in the current study, primarily in serum (Table S2). Interleukin-4 (IL-4) is known to induce wound healing macrophages and to stimulate their arginase activity [22]. The latter may be related to the observed arginine levels (Table 7 and Figure S5). Our measurements of the Methylene Dianiline treatment support the hypothesis which links putrescine to macrophages. Samples with histopathologically verified inflammation are all those at the time points 6 h and 24 h. These samples coincide with those showing an increase of putrescine (Figure 2). In support of this theory, the increase of polyamines and amino acids in urine has already been associated with nephrotoxicity prior to observable histological kidney damage [5].

In any case, polyamines are known to induce cell proliferation, which is in agreement with some of the observed gene expression modulations in the liver indicative of cell cycle regulation and tissue repair. Among the top 10 most significantly regulated genes (Table 8 and Figure S6), the zinc finger transcription factor Egr1 (early growth response 1 or Krox-24) represents an important activator of target genes involved in a variety of pathophysiological processes, including hepatocyte injury while Atf3 and Jun have also been associated with liver injury. Egr1 is among the common genes induced after a hepatocellular challenge leading to toxicity [23] and is known as immediate early growth response activated by cellular stimuli such as irradiation and small molecules [24]. Moreover, hypoxia induced up-regulation has been found in macrophages [25]. Igfbp1 (insulin-like growth factor-binding protein-1) listed as best ranked gene in Table 8 has been linked to liver regeneration [26]. It is also known to antagonize the proapoptotic effect of p53 while possibly allowing time for repair of damage or resolution of metabolic distress [27].

The results of this study show the expected response in well-known markers of DILI (AST, ALT and bilirubin) in many, but not all the treated animals. In addition, we have also identified putative biomarkers with biological anchoring such as putrescine, bile acids, and genes such as Igfbp1 and Egr1. Among the most interesting markers, bilirubin and taurocholic acid seem to strongly correlate with the occurrence of histopathological liver damage, while putrescine in serum and Igfbp1 and Egr1 in tissue were also elevated in animals showing transaminase increases or no changes. These results reinforce the usefulness of standard clinical chemistry parameters, but also show that additional parameters may improve the performance in terms of sensitivity. Further data should be collected to determine if these additional biomarkers may be also able to provide additional evidence with regards of onset, relevance, and progression of DILI in cases where ALT or AST are elevated but no tissue damage is evident microscopically.

Translational biomarkers that allow the monitoring of patients with regards of onset, progression and reversibility of toxicity would offer means to design better and safer clinical trials. In particular, sensitive, non-invasive and translational biomarkers are useful for the clinical testing of compounds which show toxicity in animals but may be safe in patients due to species differences of different dose regimens. Our set of best ranked parameters based on different technologies and different sample matrices is biologically meaningful, in agreement with previous results and shows an overall concordant measurement pattern across all samples. Also, our tailored analysis demonstrated that the identified biomarkers outperform histopathology evaluation in terms of sensitivity. Hence, this set of biomarkers can be implemented for the identification of early events leading to hepatotoxicity. Future studies increasing the number of tested compounds would improve the coverage of mechanisms causing toxicity and hence the universal validity of our findings, as the generalization power of our biomarkers may be constrained by the limited number of toxicity mechanisms which were included in the study.

## Supporting Information

Figure S1 Clinical chemistry.
(PDF)

Figure S2 Significantly changed parameters exhibiting absolute fold changes >2. Fold-changes are visualized for all samples and all significantly changed parameters (FDR <0.05) exhibiting absolute fold changes >2. The fold-changes are calculated for each sample with respect to the mean of the matching control group (vehicle treatment). Red (blue) indicate up (down)-regulation and yellow missing measurements. The samples are grouped by class as shown on the top(green: vehicle control of class negative, blue: class negative without vehicle controls, orange: increased in ALT or AST, red: class positive). The parameters are grouped by type as shown on the left(green: transcripts in liver tissue, red: metabolites in serum, gray: metabolites in liver tissue, blue: clinical chemistry in serum).
(PDF)

Figure S3 Clinical Chemistry Markers in Serum as Listed in Table 5. The fold-change of each sample is calculated with respect to the matching control group (vehicle treatment). The mean of each class is shown as horizontal line (black: negative including controls, yellow: increased in ALT or AST, red: positive). The size of the treatment symbols increase with dose. The time points are resolved by aligning the symbols in columns.
(PDF)

Figure S4 Metabolites in Liver Tissue as Listed in Table 6. The fold-change of each sample is calculated with respect to the matching control group (vehicle treatment). The mean of each class is shown as horizontal line (black: negative including controls, yellow: increased in ALT or AST, red: positive). The size of the treatment symbols increase with dose. The time points are resolved by aligning the symbols in columns.
(PDF)

Figure S5 Metabolites in Serum as Listed in Table 7. The fold-change of each sample is calculated with respect to the matching control group (vehicle treatment). The mean of each class is shown as horizontal line (black: negative including controls, yellow: increased in ALT or AST, red: positive). The size of the treatment symbols increase with dose. The time points are resolved by aligning the symbols in columns.
(PDF)

Figure S6 Transcripts in Liver Tissue as Listed in Table 8. The fold-change of each sample is calculated with respect to the matching control group (vehicle treatment). The mean of each class is shown as horizontal line (black: negative including controls, yellow: increased in ALT or AST, red: positive). The size of the treatment symbols increase with dose. The time points are resolved by aligning the symbols in columns.
(PDF)

Table S1 Significantly changed metabolites in liver tissue. All 63 liver metabolites for which a structure ID was identified or proposed and which were significantly changed (FDR <0.05) are listed.
(XLSX)

Table S2 Significantly changed metabolites in serum. All 85 serum metabolites for which a structure ID was identified or proposed and which were significantly changed (FDR <0.05) are listed.
(XLSX)

Table S3 Significantly changed transcripts in liver tissue. All 45 genes exhibiting absolute fold changes >2 and being significantly changed (FDR <0.05) are listed.
(XLSX)
