# Supplementary material for: Identification of Metabolites, Clinical Chemistry Markers and Transcripts Associated with Hepatotoxicity
Source: PLoS One. 2014 May 16;9(5):e97249. doi: 10.1371/journal.pone.0097249 (PMC4023975; doi:10.1371/journal.pone.0097249)
Supplement: Figure S1 — Clinical chemistry. (PDF) [file pone.0097249.s001.pdf]

SERUM AGLOB (calibrated with respect to matching vehicle group)

Samples are separated by treatment group and dose level (dashed grey lines) and ordered by time point

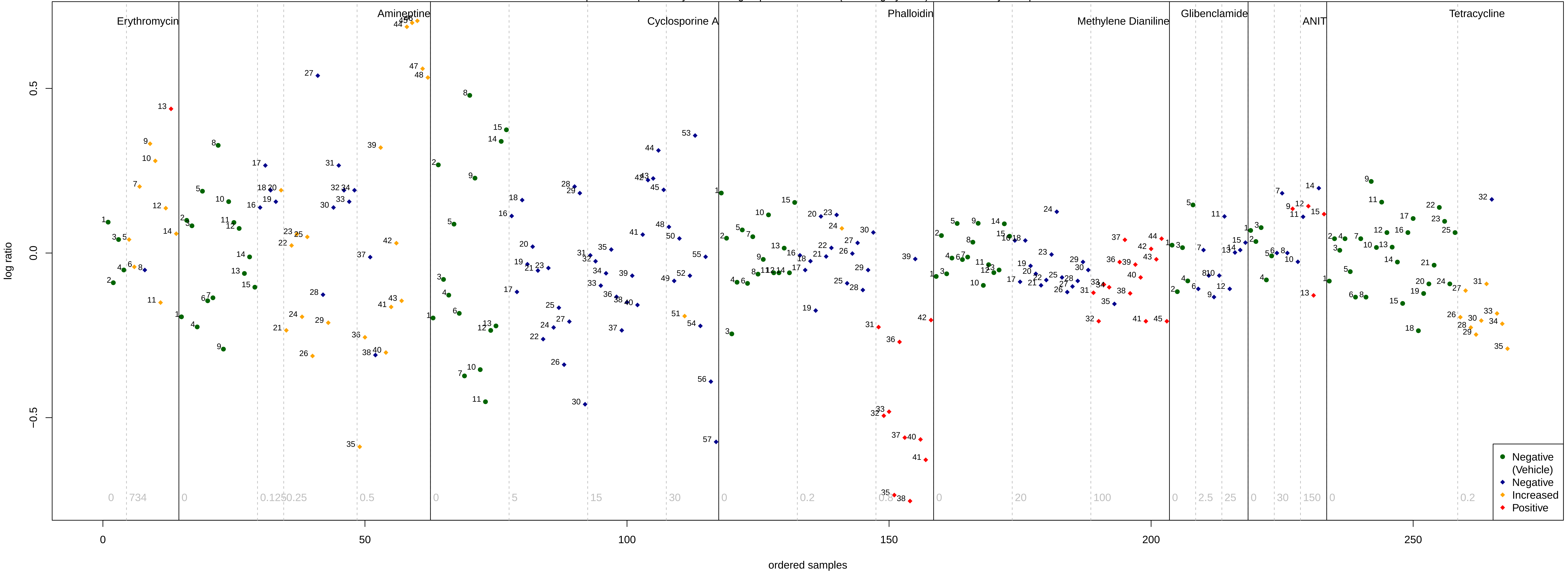

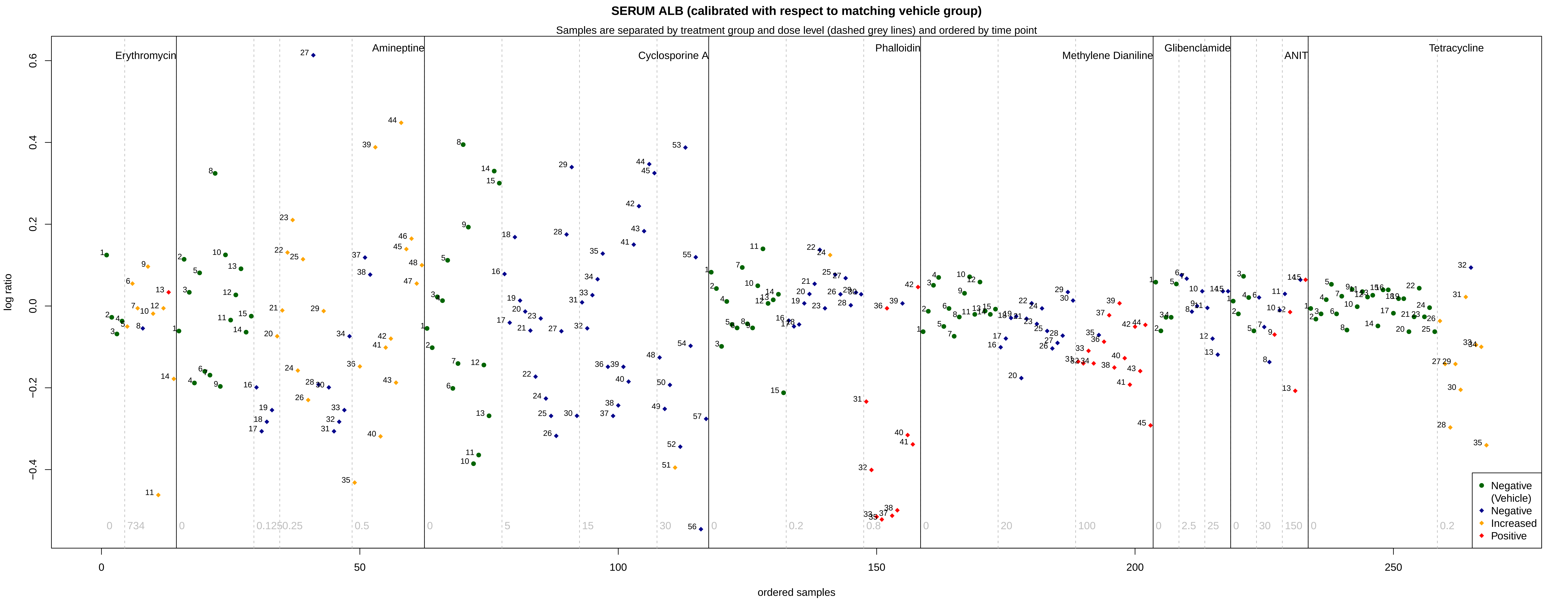

SERUM ALB (calibrated with respect to matching vehicle group)
Samples are separated by treatment group and dose level (dashed grey lines) and ordered by time point
Erythromycin
Amineptine
Cyclosporine A
Phalloidin
Methylene Dianiline
Glibenclamide
ANIT
Tetracycline
log ratio
ordered samples
Negative (Vehicle)
Negative
Increased
Positive

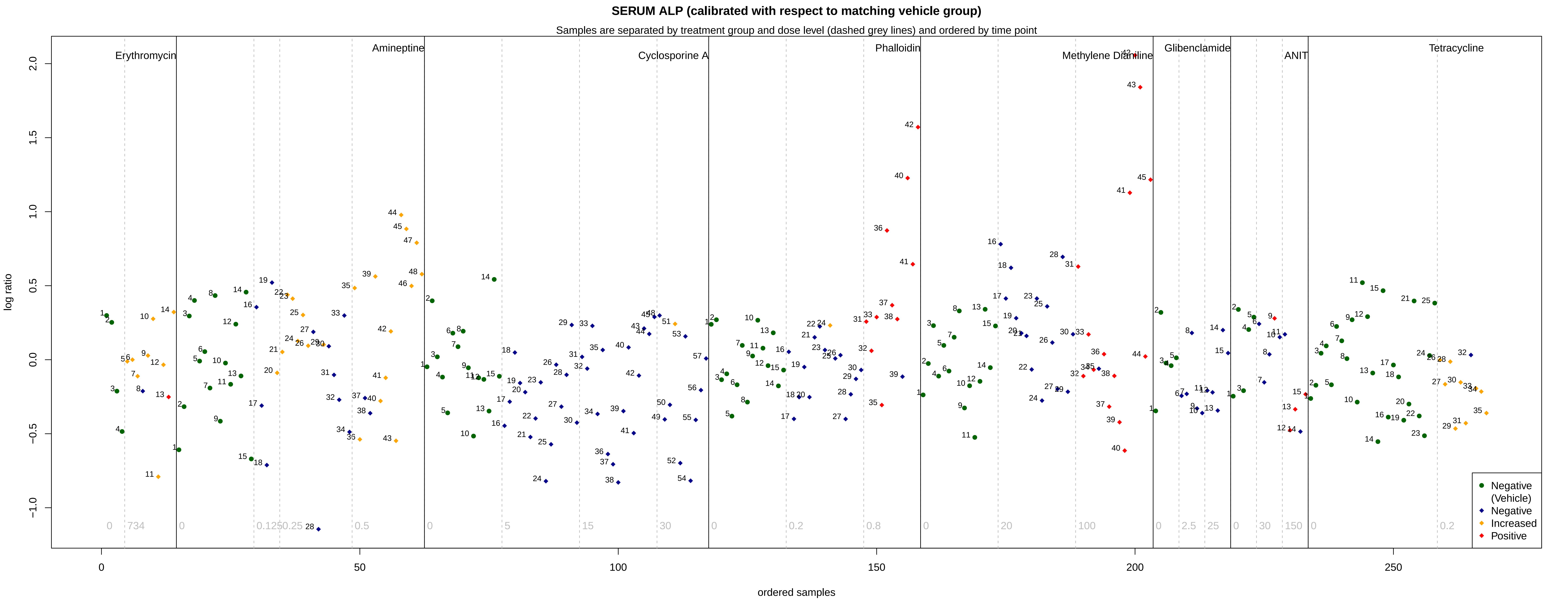
SERUM ALP (calibrated with respect to matching vehicle group)
Samples are separated by treatment group and dose level (dashed grey lines) and ordered by time point
Erythromycin
Amineptine
Cyclosporine A
Phalloidin
Methylene Dianiline
Glibenclamide
ANIT
Tetracycline
log ratio
ordered samples
0
50
100
150
200
250
Negative (Vehicle)
Negative
Increased
Positive

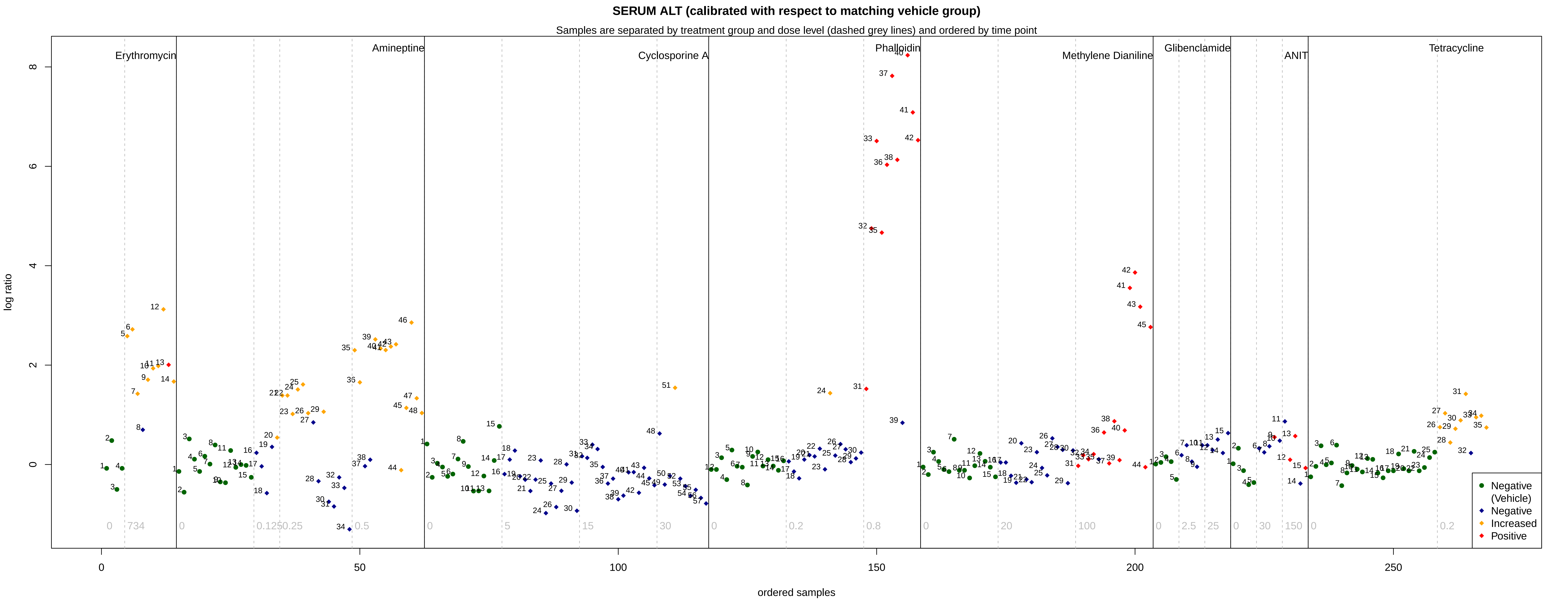
SERUM ALT (calibrated with respect to matching vehicle group)
Samples are separated by treatment group and dose level (dashed grey lines) and ordered by time point
Erythromycin
Amineptine
Cyclosporine A
Phalloidin
Methylene Dianiline
Glibenclamide
ANIT
Tetracycline
log ratio
ordered samples
Negative (Vehicle)
Negative
Increased
Positive

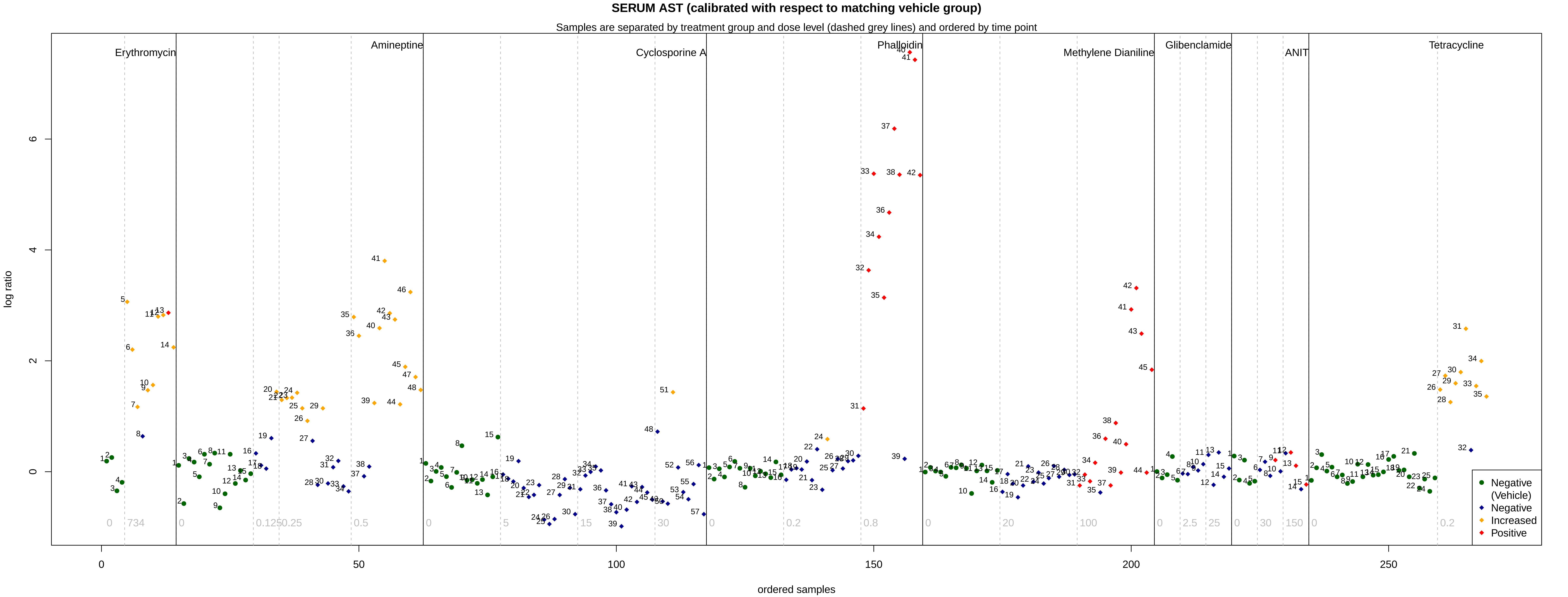
SERUM AST (calibrated with respect to matching vehicle group)
Samples are separated by treatment group and dose level (dashed grey lines) and ordered by time point
Erythromycin
Amineptine
Cyclosporine A
Phalloidin
Methylene Dianiline
Glibenclamide
ANIT
Tetracycline
log ratio
ordered samples
Negative (Vehicle)
Negative
Increased
Positive

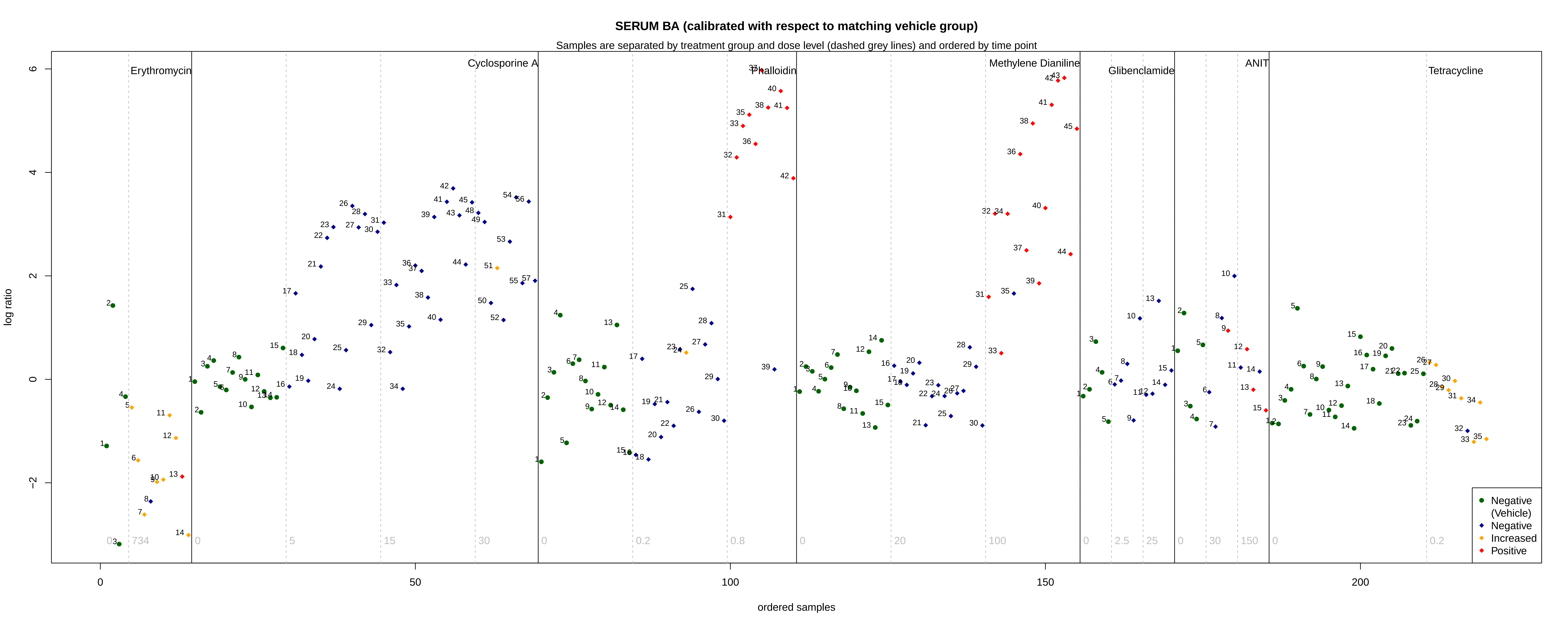
SERUM BA (calibrated with respect to matching vehicle group)
Samples are separated by treatment group and dose level (dashed grey lines) and ordered by time point
Erythromycin
Cyclosporine A
Phalloidin
Methylene Dianiline
Glibenclamide
ANIT
Tetracycline
log ratio
ordered samples
0
50
100
150
200
6
4
2
0
−2
0
5
15
30
0
0.2
0.8
0
20
100
0
2.5
25
0
30
150
0
0.2
Negative (Vehicle)
Negative
Increased
Positive

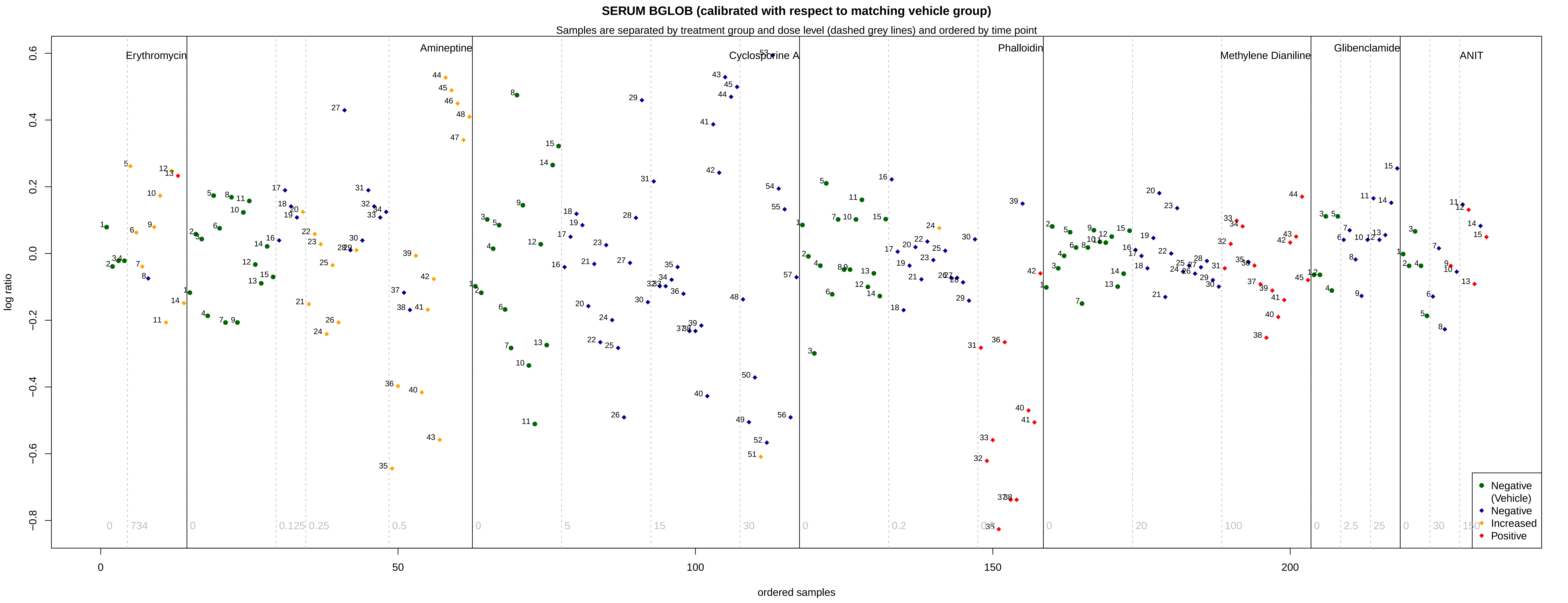
SERUM BGLOB (calibrated with respect to matching vehicle group)
Samples are separated by treatment group and dose level (dashed grey lines) and ordered by time point
Erythromycin
Amineptine
Cyclosporine A
Phalloidin
Methylene Dianiline
Glibenclamide
ANIT
log ratio
ordered samples
Negative (Vehicle)
Negative
Increased
Positive

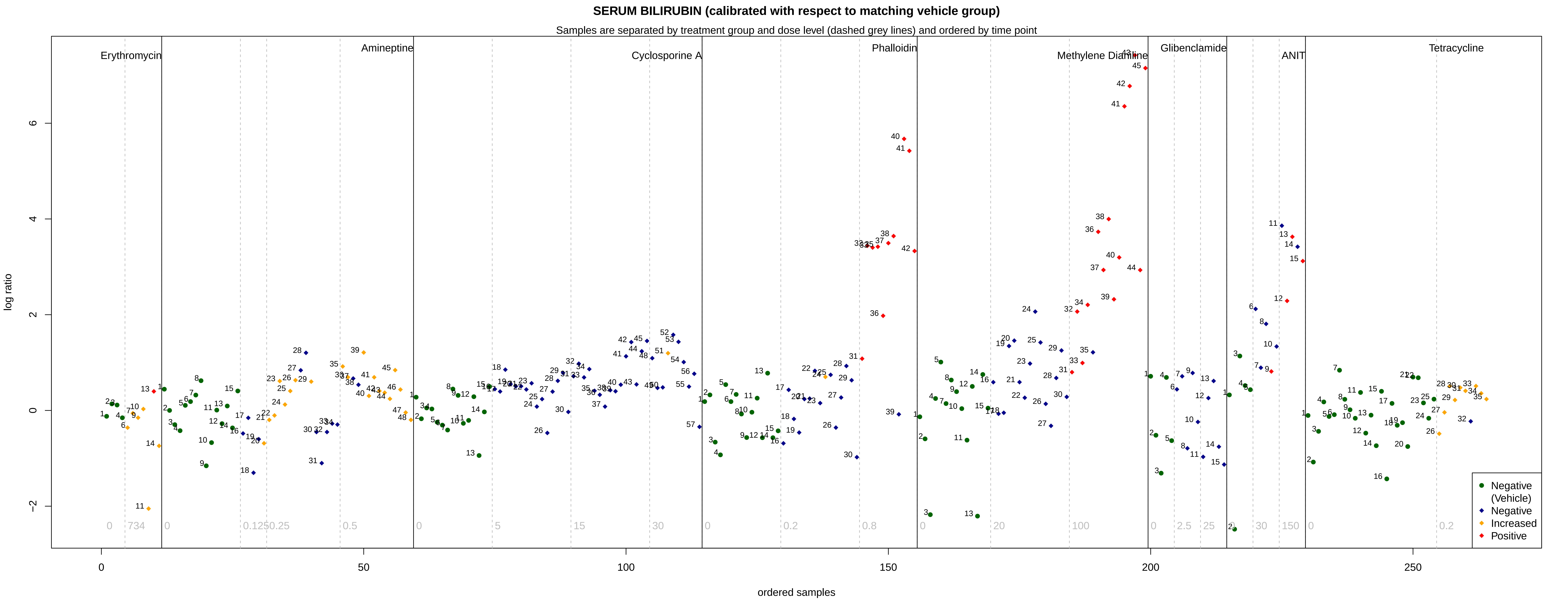

SERUM BILIRUBIN (calibrated with respect to matching vehicle group)
Samples are separated by treatment group and dose level (dashed grey lines) and ordered by time point
Erythromycin
Amineptine
Cyclosporine A
Phalloidin
Methylene Dianiline
Glibenclamide
ANIT
Tetracycline
log ratio
ordered samples
Negative (Vehicle)
Negative
Increased
Positive

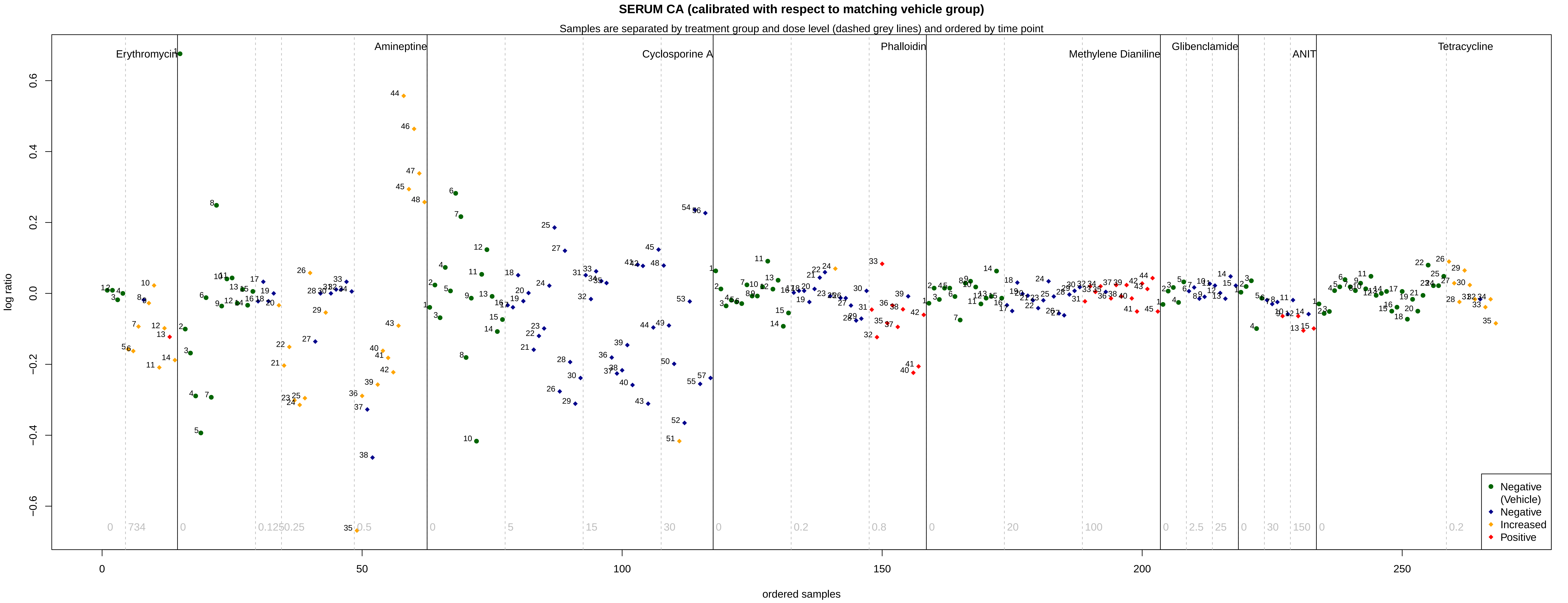
SERUM CA (calibrated with respect to matching vehicle group)
Samples are separated by treatment group and dose level (dashed grey lines) and ordered by time point
Erythromycin
Amineptine
Cyclosporine A
Phalloidin
Methylene Dianiline
Glibenclamide
ANIT
Tetracycline
log ratio
ordered samples
Negative (Vehicle)
Negative
Increased
Positive

SERUM CHOLESTEROL (calibrated with respect to matching vehicle group)

Samples are separated by treatment group and dose level (dashed grey lines) and ordered by time point

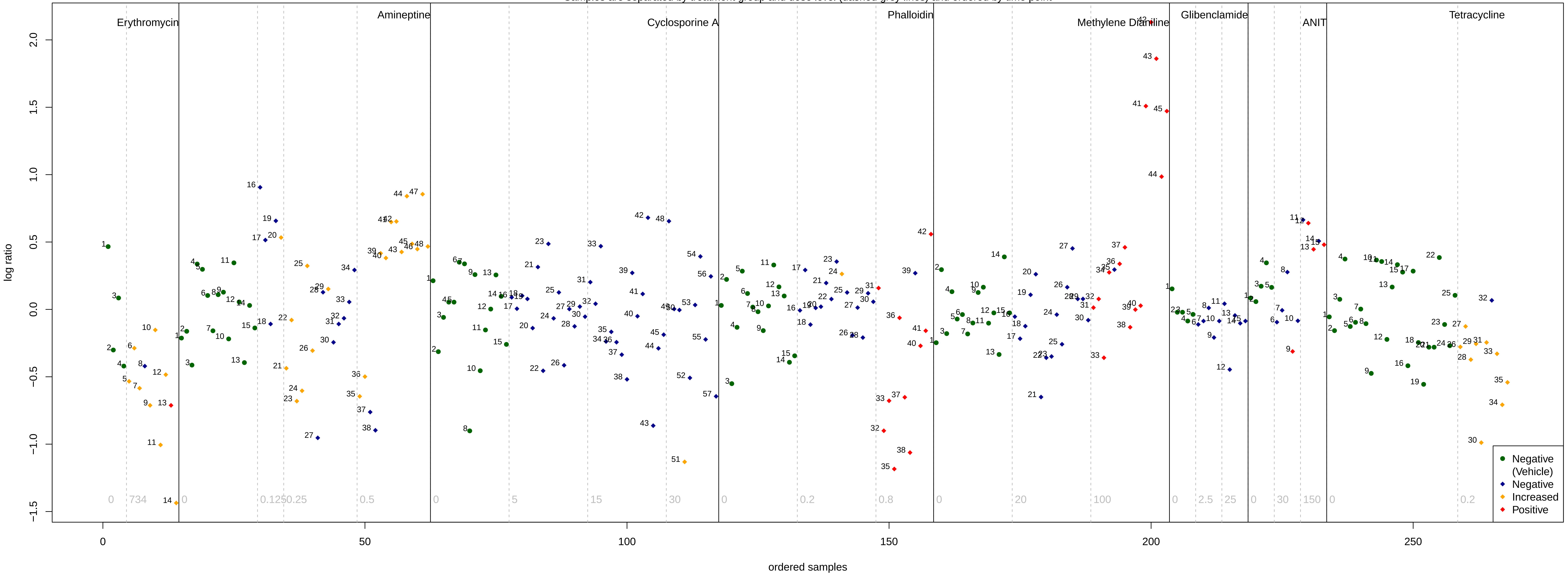

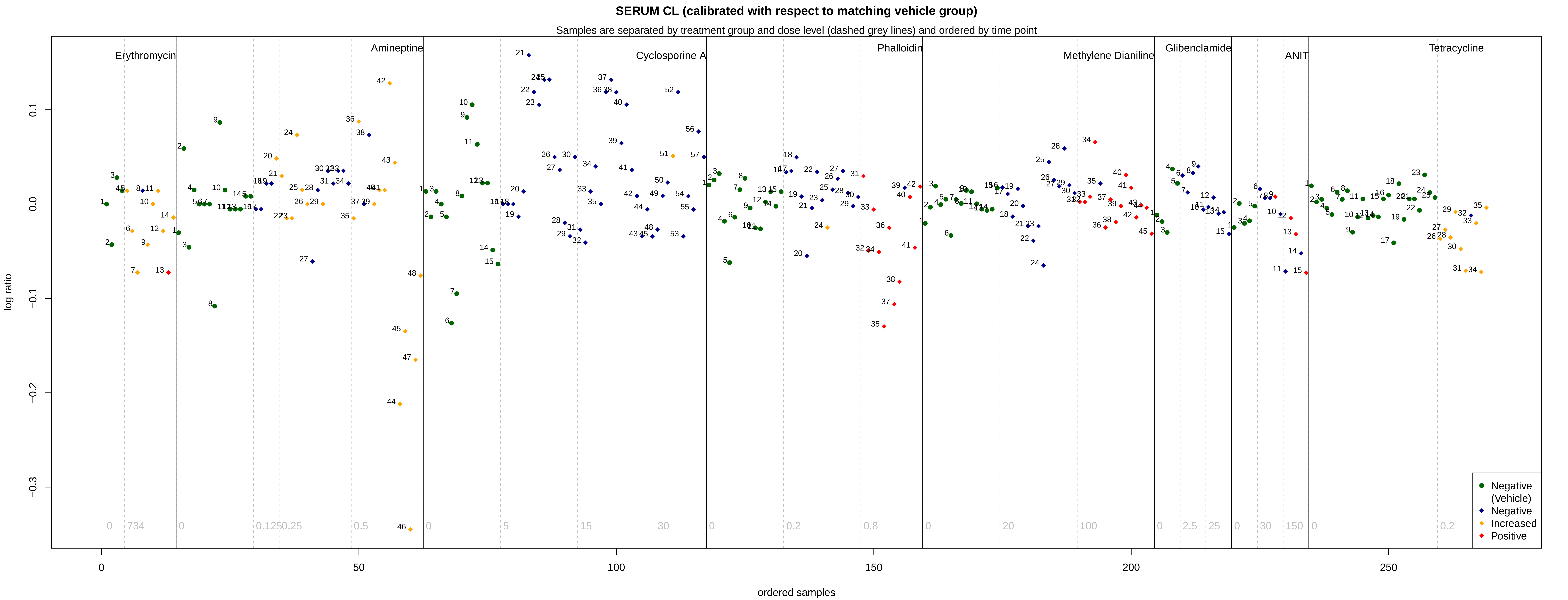
SERUM CL (calibrated with respect to matching vehicle group)
Samples are separated by treatment group and dose level (dashed grey lines) and ordered by time point
Erythromycin
Amineptine
Cyclosporine A
Phalloidin
Methylene Dianiline
Glibenclamide
ANIT
Tetracycline
log ratio
ordered samples
Negative (Vehicle)
Negative
Increased
Positive

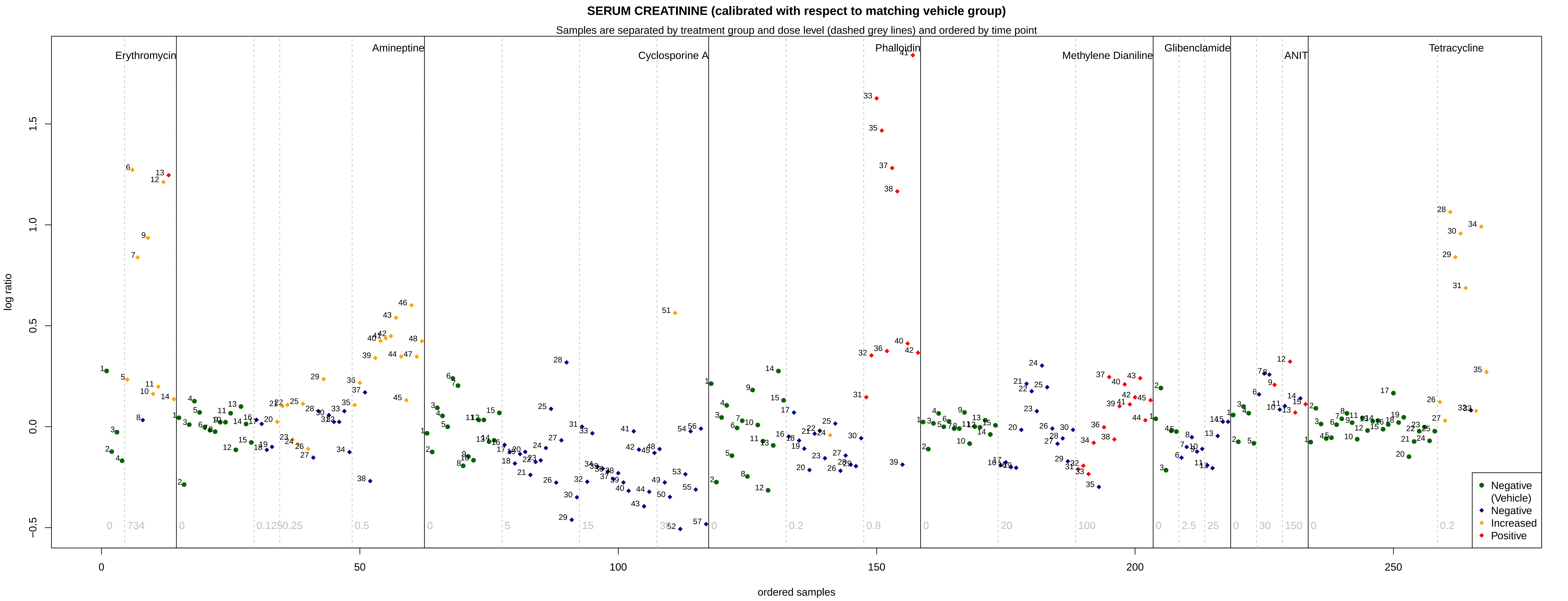

SERUM CREATININE (calibrated with respect to matching vehicle group)
Samples are separated by treatment group and dose level (dashed grey lines) and ordered by time point
Erythromycin
Amineptine
Cyclosporine A
Phalloidin
Methylene Dianiline
Glibenclamide
ANIT
Tetracycline
log ratio
ordered samples
Negative (Vehicle)
Negative
Increased
Positive

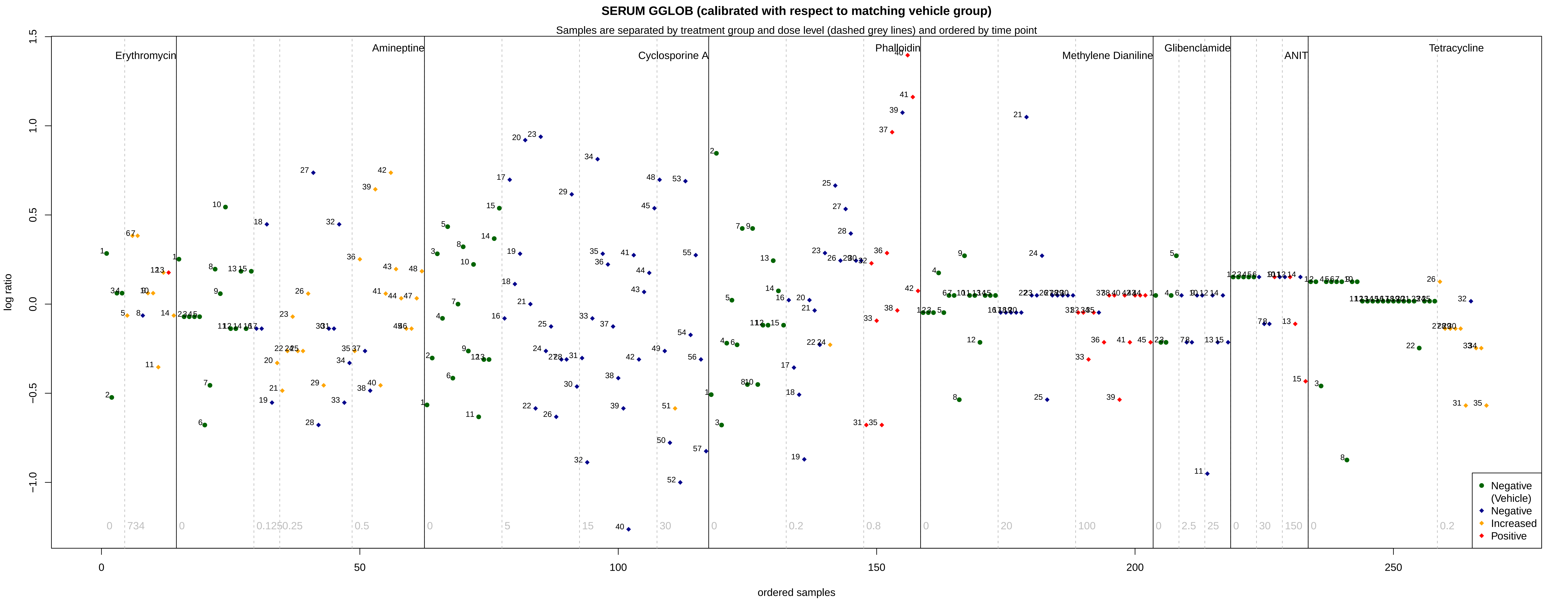

SERUM GGLOB (calibrated with respect to matching vehicle group)
Samples are separated by treatment group and dose level (dashed grey lines) and ordered by time point
Erythromycin
Aminepetine
Cyclosporine A
Phalloidin
Methylene Dianiline
Glibenclamide
ANIT
Tetracycline
log ratio
ordered samples
Negative (Vehicle)
Negative
Increased
Positive

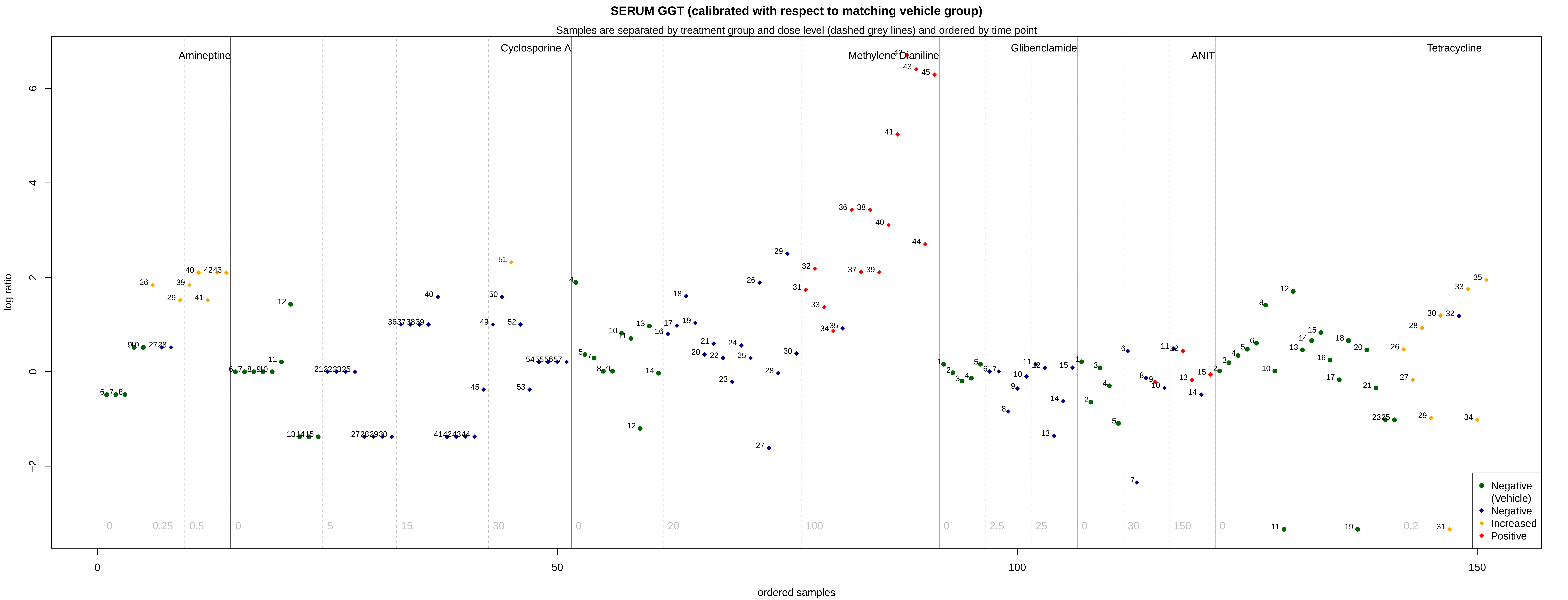
SERUM GGT (calibrated with respect to matching vehicle group)
Samples are separated by treatment group and dose level (dashed grey lines) and ordered by time point
Amineptine
Cyclosporine A
Methylene Dianiline
Glibenclamide
ANIT
Tetracycline
log ratio
ordered samples
Negative (Vehicle)
Negative
Increased
Positive

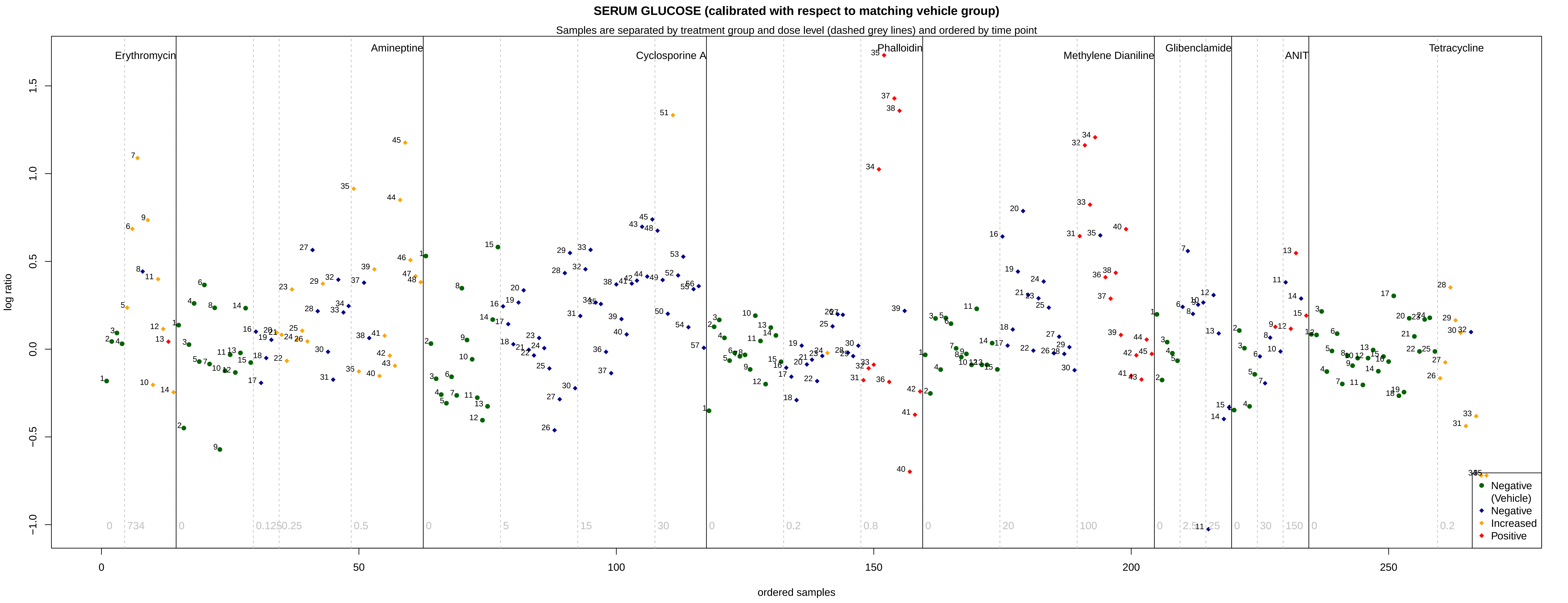
SERUM GLUCOSE (calibrated with respect to matching vehicle group)
Samples are separated by treatment group and dose level (dashed grey lines) and ordered by time point
Erythromycin
Amineptine
Cyclosporine A
Phalloidin
Methylene Dianiline
Glibenclamide
ANIT
Tetracycline
log ratio
ordered samples
Negative (Vehicle)
Negative
Increased
Positive

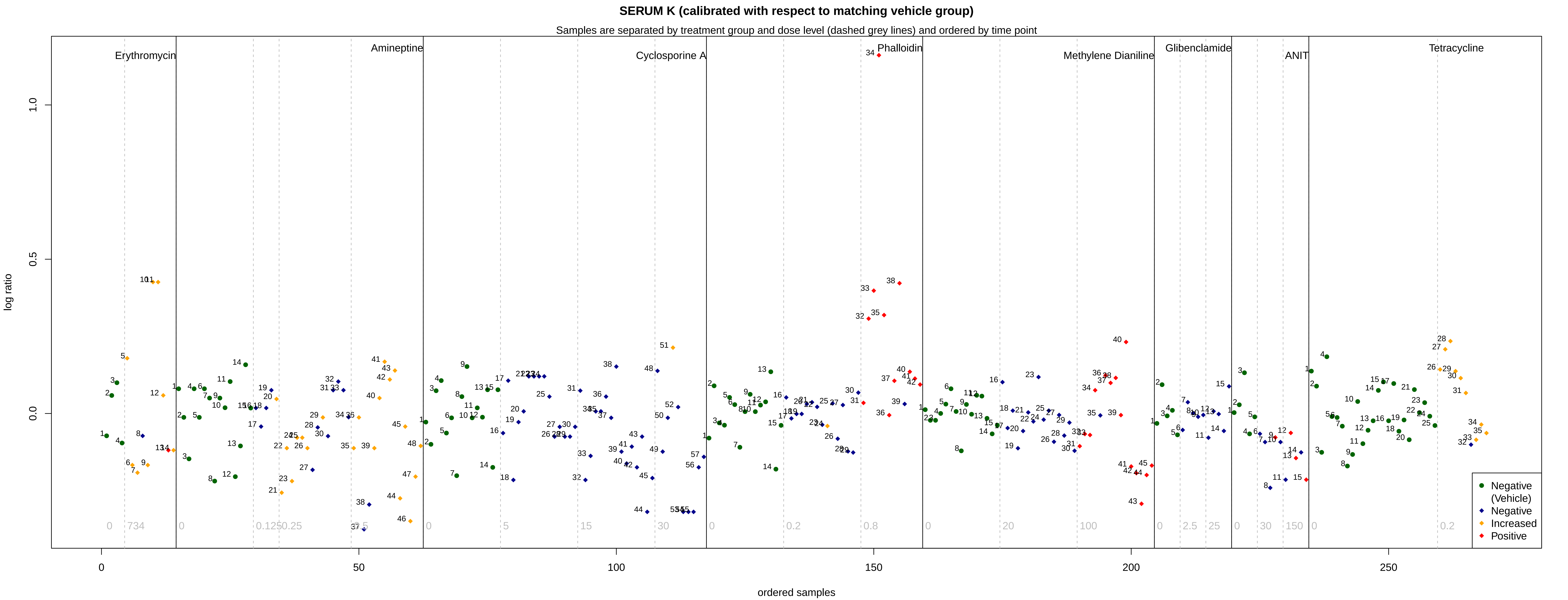
SERUM K (calibrated with respect to matching vehicle group)
Samples are separated by treatment group and dose level (dashed grey lines) and ordered by time point
log ratio
ordered samples
0.0
0.5
1.0
0
50
100
150
200
250
Erythromycin
Amineptine
Cyclosporine A
Phalloidin
Methylene Dianiline
Glibenclamide
ANIT
Tetracycline
Negative (Vehicle)
Negative
Increased
Positive

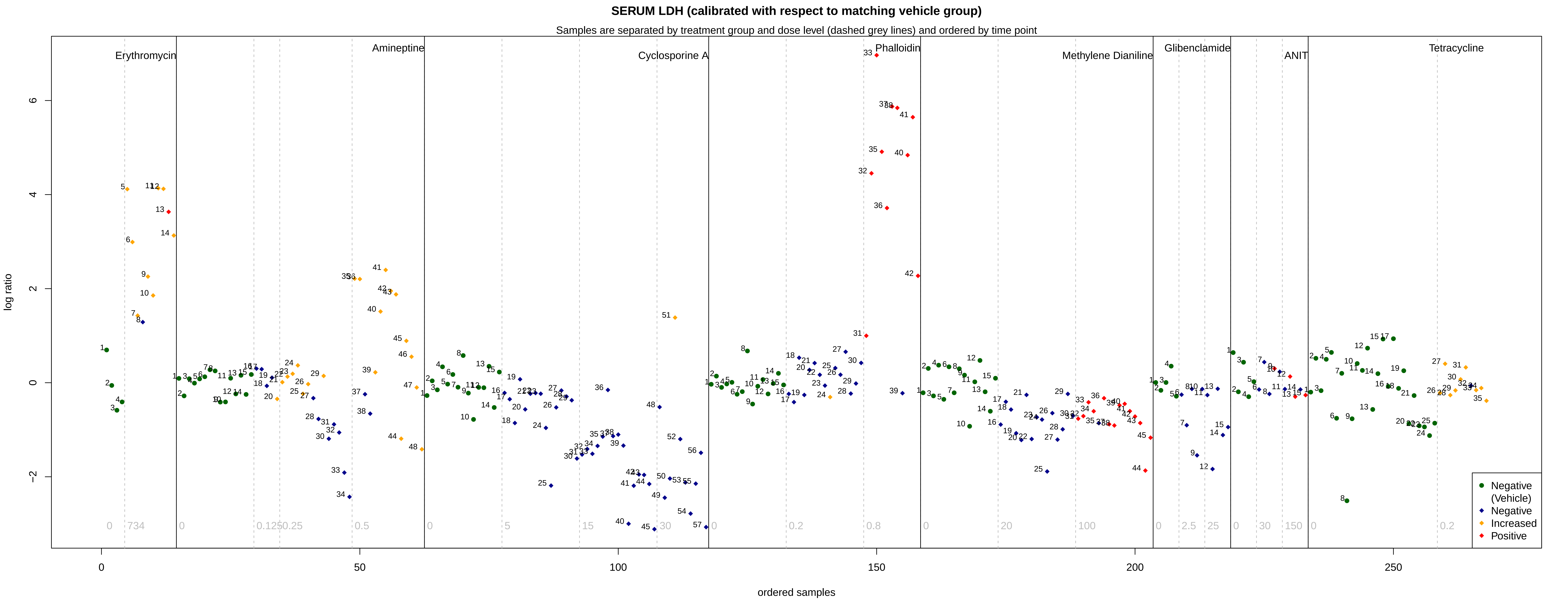
SERUM LDH (calibrated with respect to matching vehicle group)
Samples are separated by treatment group and dose level (dashed grey lines) and ordered by time point
Erythromycin
Amineptine
Cyclosporine A
Phalloidin
Methylene Dianiline
Glibenclamide
ANIT
Tetracycline
log ratio
ordered samples
Negative (Vehicle)
Negative
Increased
Positive

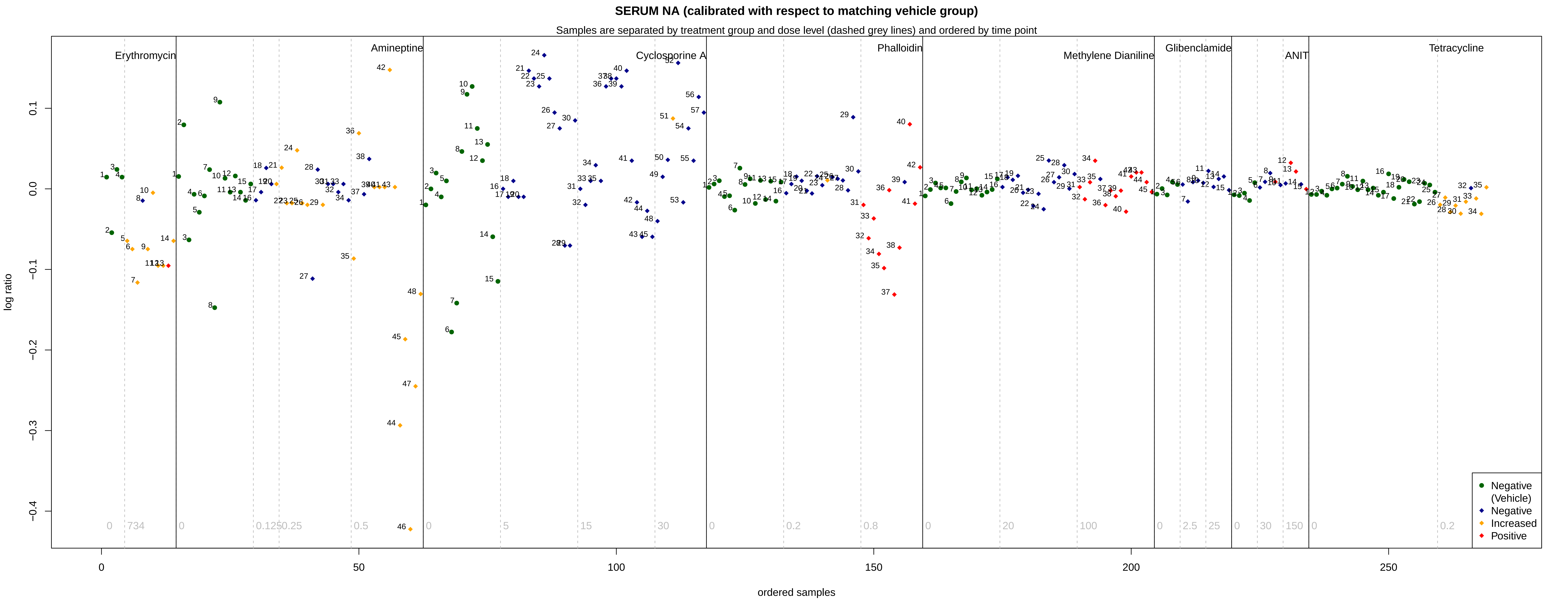
SERUM NA (calibrated with respect to matching vehicle group)
Samples are separated by treatment group and dose level (dashed grey lines) and ordered by time point
Erythromycin
Amineptine
Cyclosporine A
Phalloidin
Methylene Dianiline
Glibenclamide
ANIT
Tetracycline
log ratio
ordered samples
Negative (Vehicle)
Negative
Increased
Positive

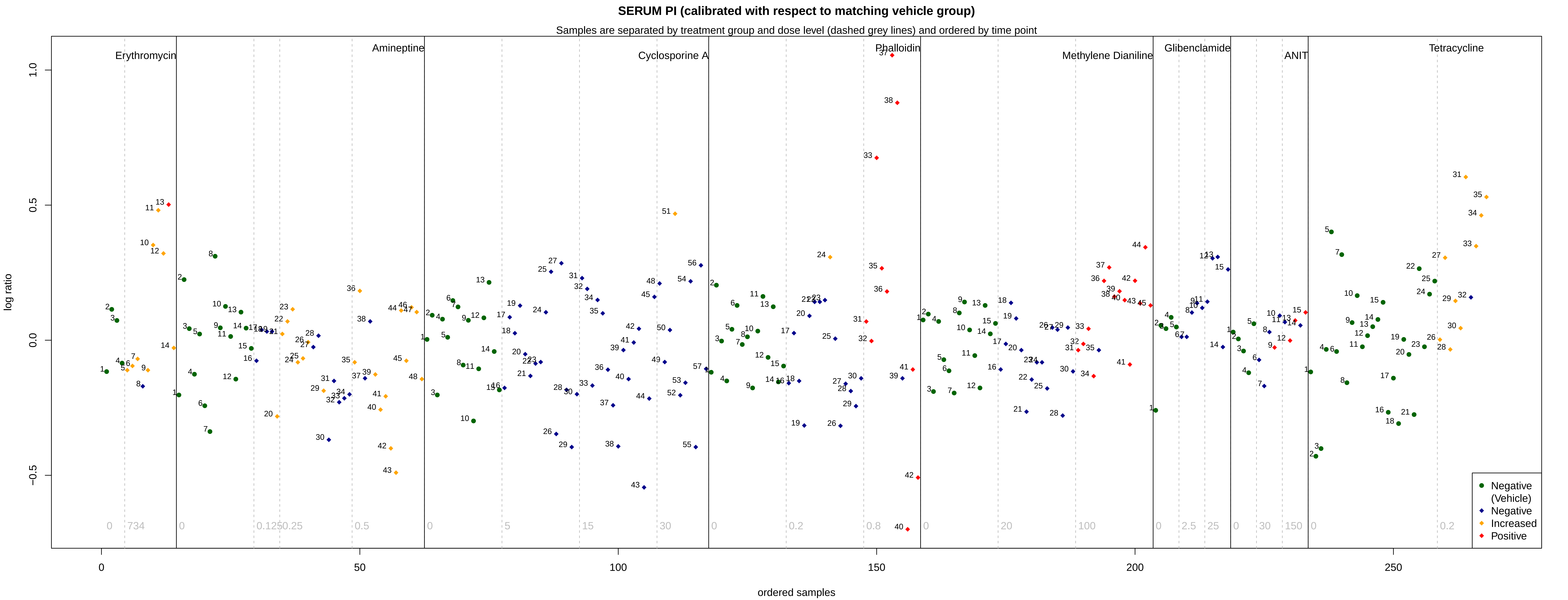
SERUM PI (calibrated with respect to matching vehicle group)
Samples are separated by treatment group and dose level (dashed grey lines) and ordered by time point
Erythromycin
Amineptine
Cyclosporine A
Phalloidin
Methylene Dianiline
Glibenclamide
ANIT
Tetracycline
log ratio
ordered samples
Negative (Vehicle)
Negative
Increased
Positive

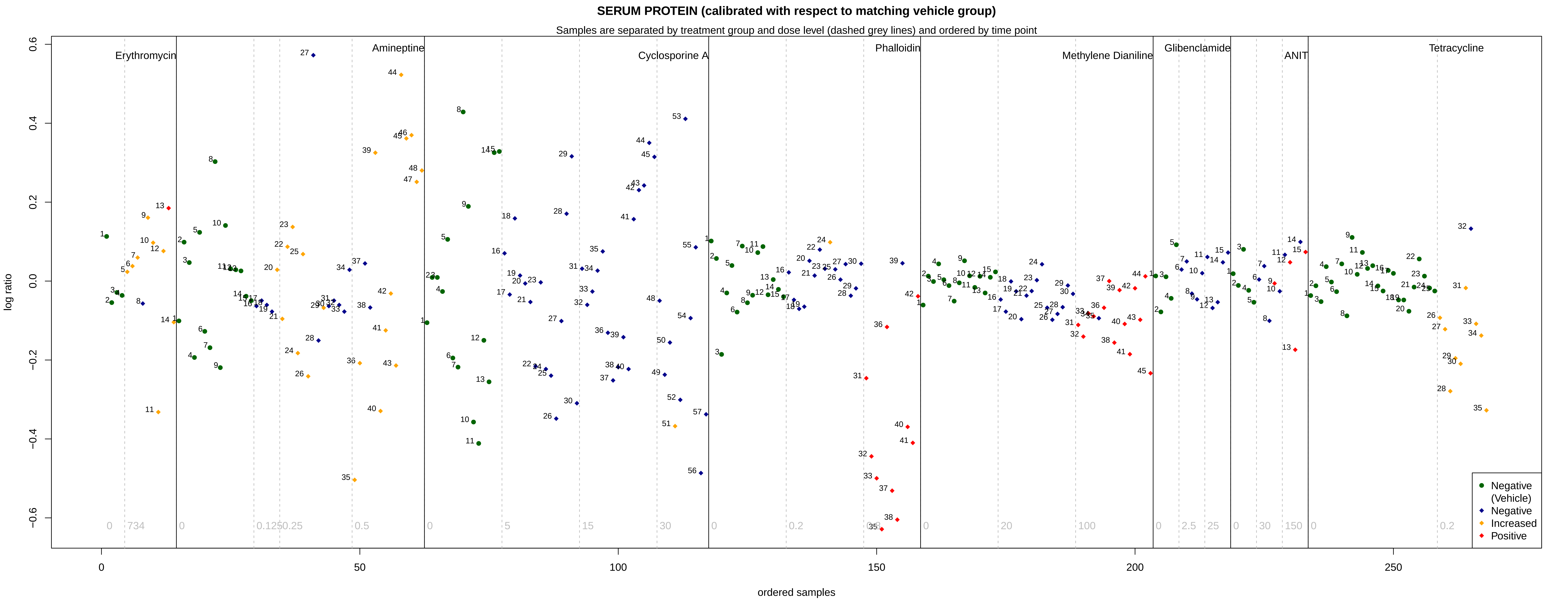
SERUM PROTEIN (calibrated with respect to matching vehicle group)
Samples are separated by treatment group and dose level (dashed grey lines) and ordered by time point
Erythromycin
Amineptine
Cyclosporine A
Phalloidin
Methylene Dianiline
Glibenclamide
ANIT
Tetracycline
log ratio
ordered samples
Negative (Vehicle)
Negative
Increased
Positive

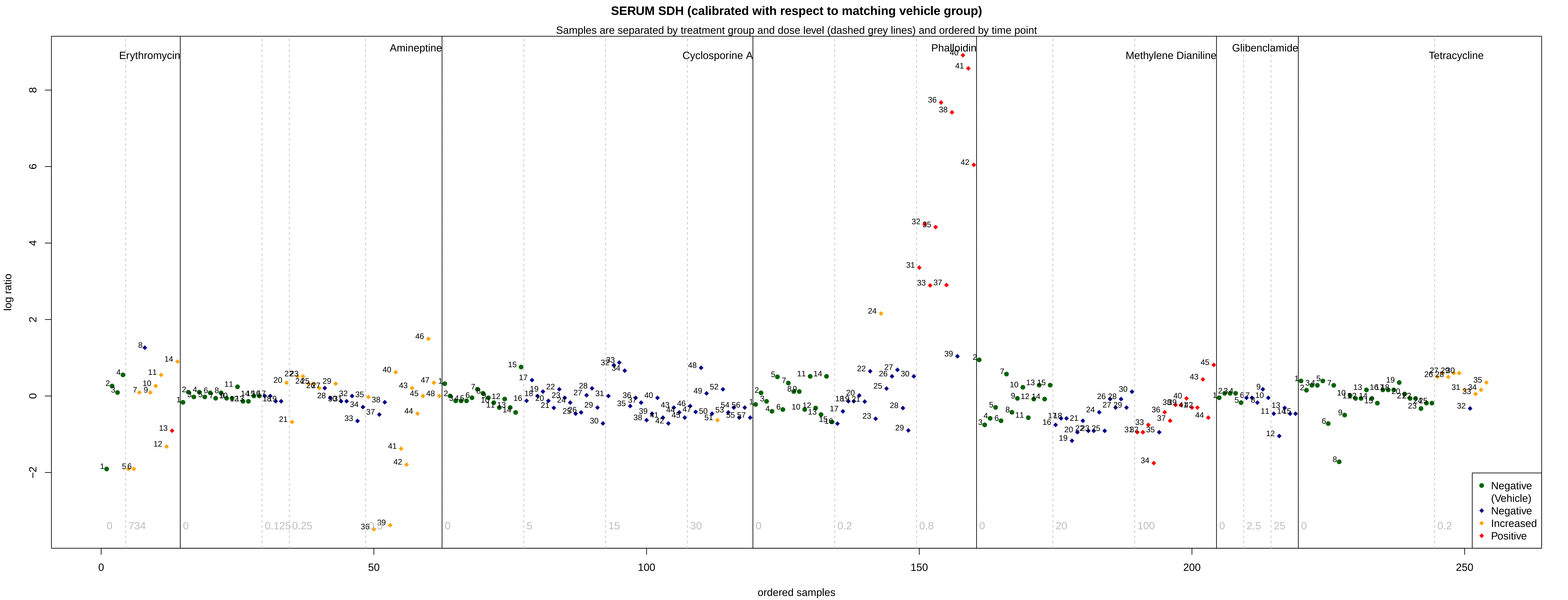

SERUM SDH (calibrated with respect to matching vehicle group)
Samples are separated by treatment group and dose level (dashed grey lines) and ordered by time point
Erythromycin
Amineptine
Cyclosporine A
Phalloidin
Methylene Dianiline
Glibenclamide
Tetracycline
log ratio
ordered samples
Negative (Vehicle)
Negative
Increased
Positive

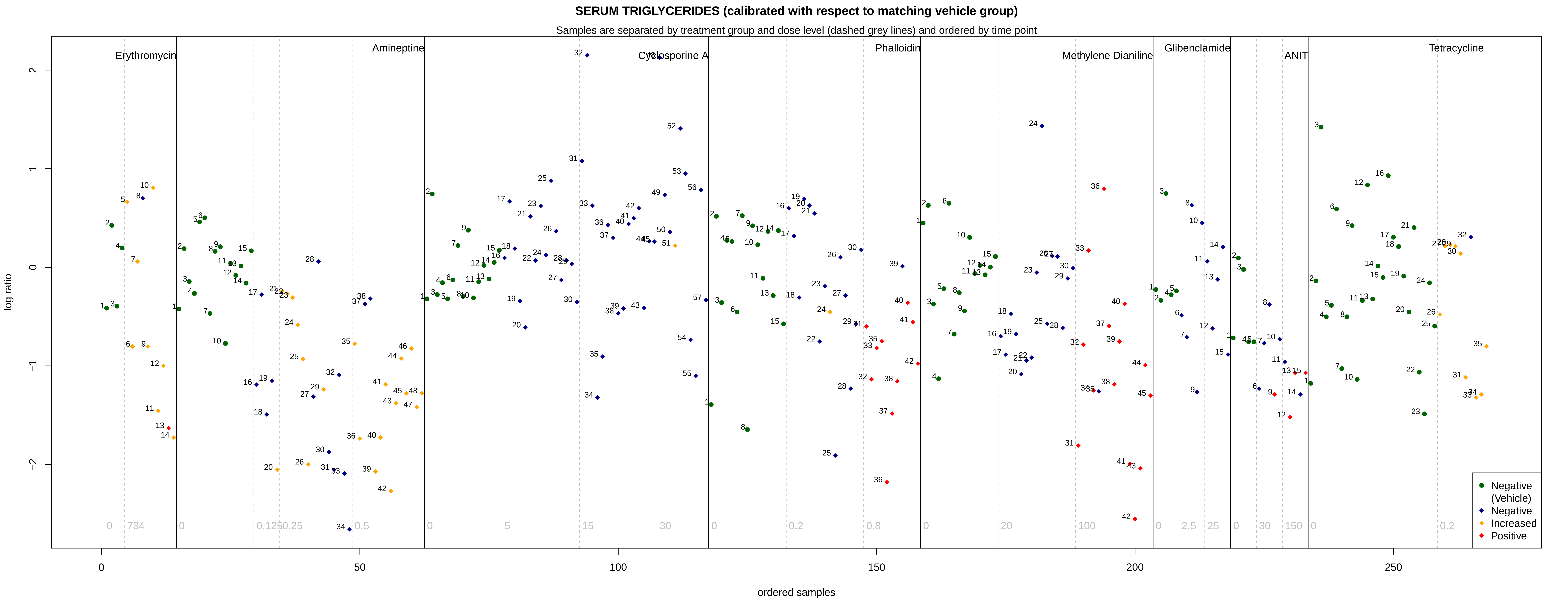
SERUM TRIGLYCERIDES (calibrated with respect to matching vehicle group)
Samples are separated by treatment group and dose level (dashed grey lines) and ordered by time point
Erythromycin
Amineptine
Cyclosporine A
Phalloidin
Methylene Dianiline
Glibenclamide
ANIT
Tetracycline
log ratio
ordered samples
Negative (Vehicle)
Negative
Increased
Positive

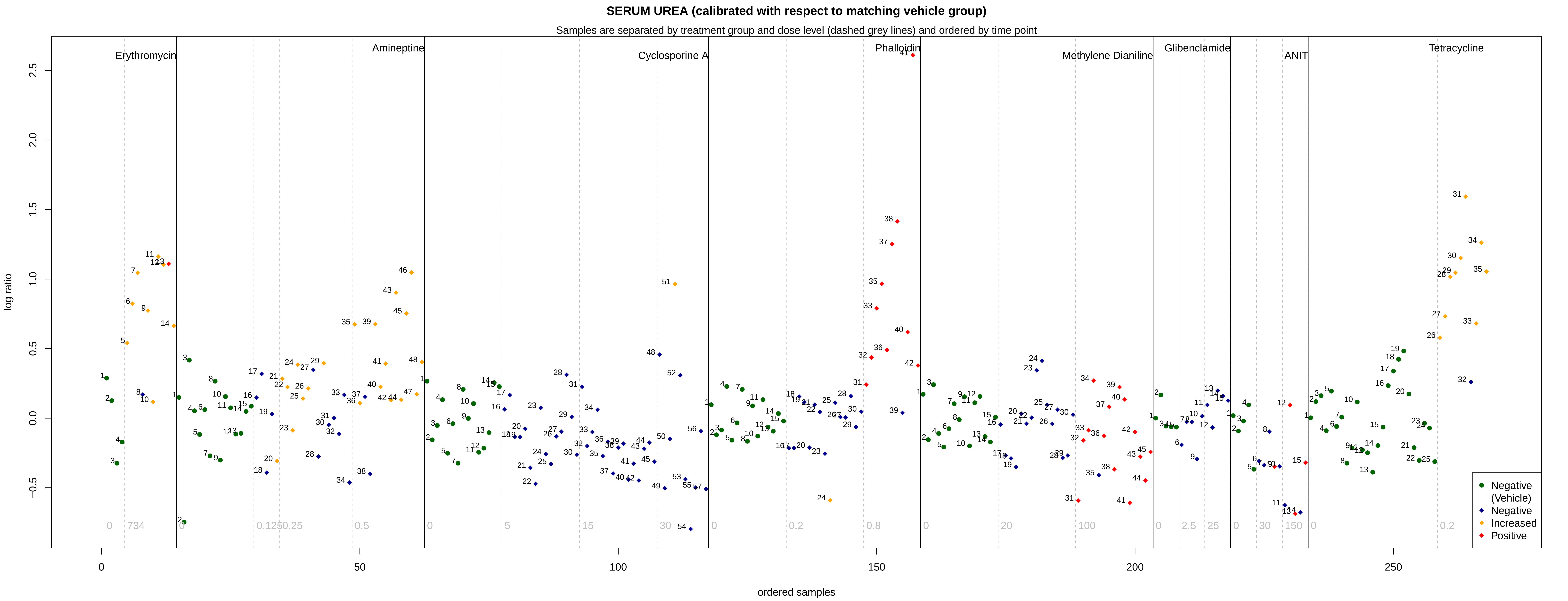
SERUM UREA (calibrated with respect to matching vehicle group)
Samples are separated by treatment group and dose level (dashed grey lines) and ordered by time point
Erythromycin
Amineptine
Cyclosporine A
Phalloidin
Methylene Dianiline
Glibenclamide
ANIT
Tetracycline
log ratio
ordered samples
Negative (Vehicle)
Negative
Increased
Positive
