# Supplementary figures and images for: Identification of Metabolites, Clinical Chemistry Markers and Transcripts Associated with Hepatotoxicity
Source: PLoS One. 2014 May 16;9(5):e97249. doi: 10.1371/journal.pone.0097249 (PMC4023975; doi:10.1371/journal.pone.0097249)

# Significantly Changed Parameters

Parameters (grouped by type)

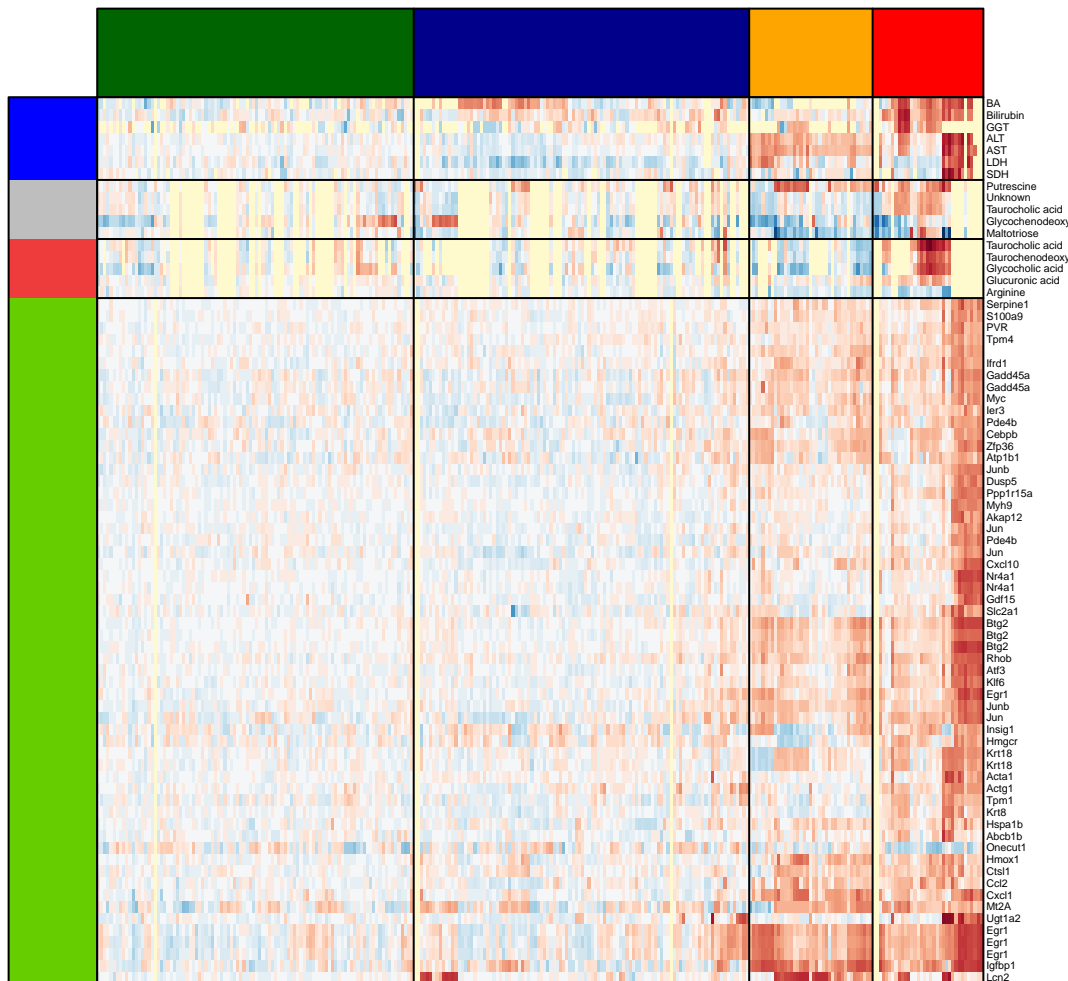

Samples (grouped by class)

Supplement: Figure S2 — Significantly changed parameters exhibiting absolute fold changes >2. Fold-changes are visualized for all samples and all significantly changed parameters (FDR <0.05) exhibiting absolute fold changes >2. The fold-changes are calculated for each sample with respect to the mean of the matching control group (vehicle treatment). Red (blue) indicate up (down)-regulation and yellow missing measurements. The samples are grouped by class as shown on the top(green: vehicle control of class negative, blue: class negative without vehicle controls, orange: increased in ALT or AST, red: class positive). The parameters are grouped by type as shown on the left(green: transcripts in liver tissue, red: metabolites in serum, gray: metabolites in liver tissue, blue: clinical chemistry in serum). (PDF) [file pone.0097249.s002.pdf]
