# Supplementary material for: Identification of Metabolites, Clinical Chemistry Markers and Transcripts Associated with Hepatotoxicity
Source: PLoS One. 2014 May 16;9(5):e97249. doi: 10.1371/journal.pone.0097249 (PMC4023975; doi:10.1371/journal.pone.0097249)
Supplement: Figure S4 — Metabolites in Liver Tissue as Listed in Table 6. The fold-change of each sample is calculated with respect to the matching control group (vehicle treatment). The mean of each class is shown as horizontal line (black: negative including controls, yellow: increased in ALT or AST, red: positive). The size of the treatment symbols increase with dose. The time points are resolved by aligning the symbols in columns. (PDF) [file pone.0097249.s004.pdf]

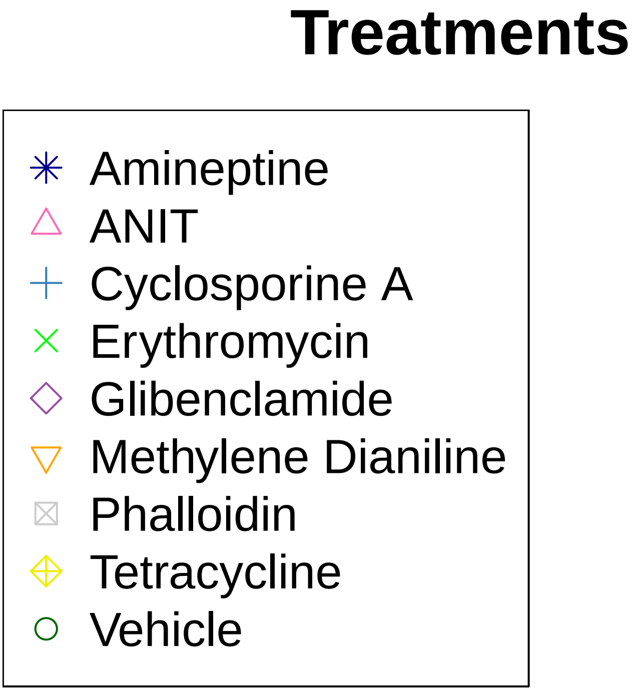
Treatments
Amineptine
ANIT
Cyclosporine A
Erythromycin
Glibenclamide
Methylene Dianiline
Phalloidin
Tetracycline
Vehicle

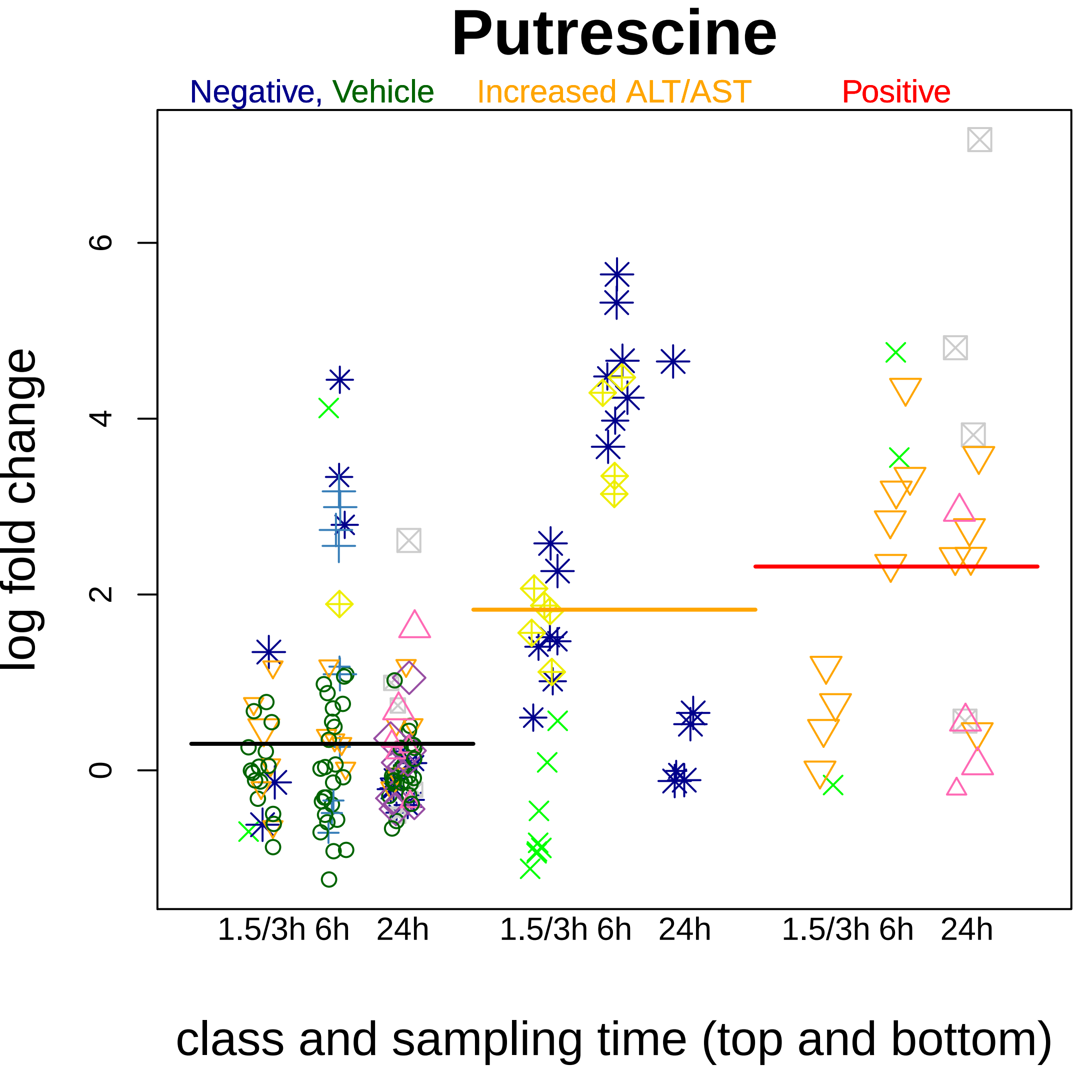

Putrescine
Negative, Vehicle
Increased ALT/AST
Positive
log fold change
0
2
4
6
1.5/3h 6h 24h
1.5/3h 6h 24h
1.5/3h 6h 24h
class and sampling time (top and bottom)

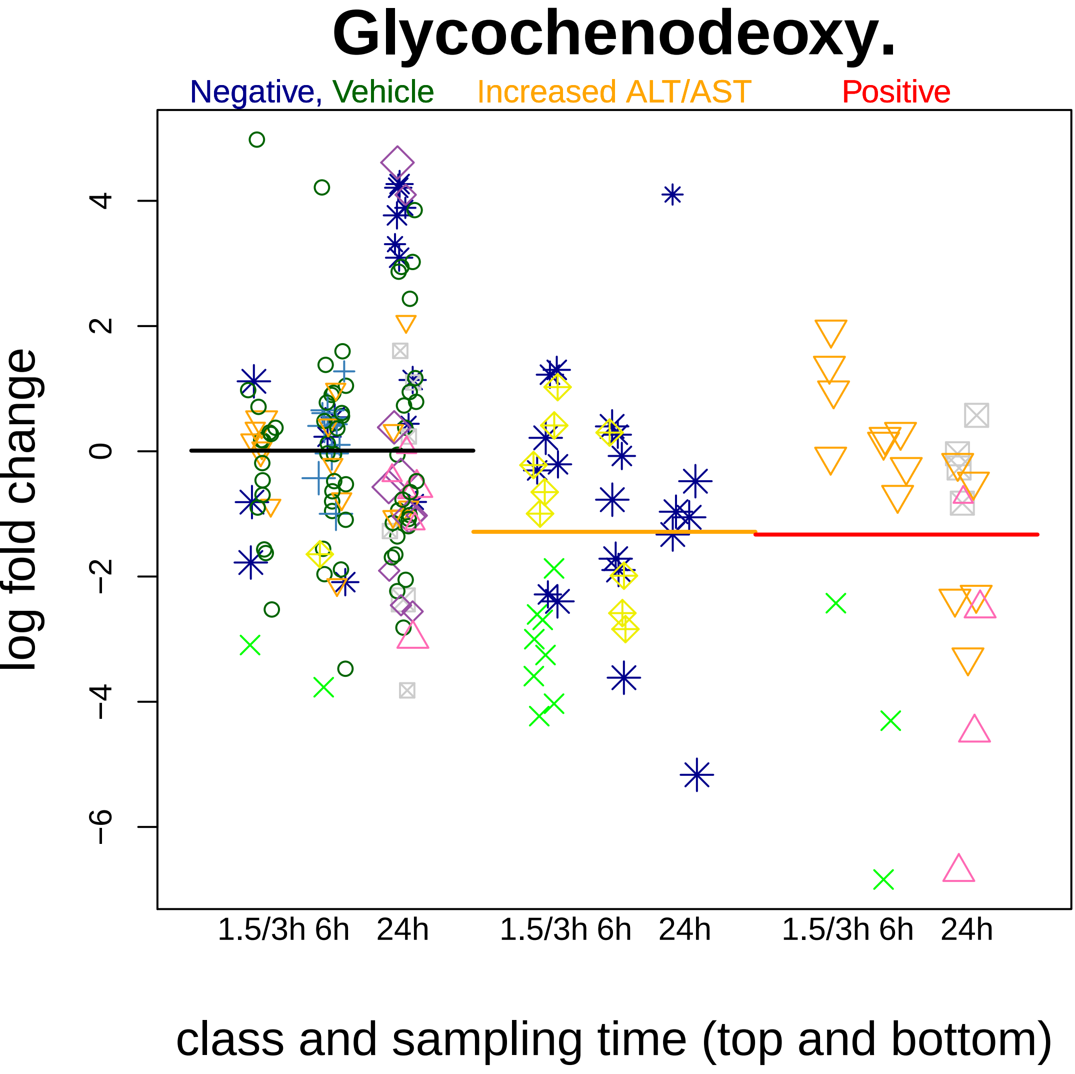
Glycochenodeoxy.
Negative, Vehicle
Increased ALT/AST
Positive
log fold change
4
2
0
−2
−4
−6
1.5/3h 6h 24h
1.5/3h 6h 24h
1.5/3h 6h 24h
class and sampling time (top and bottom)

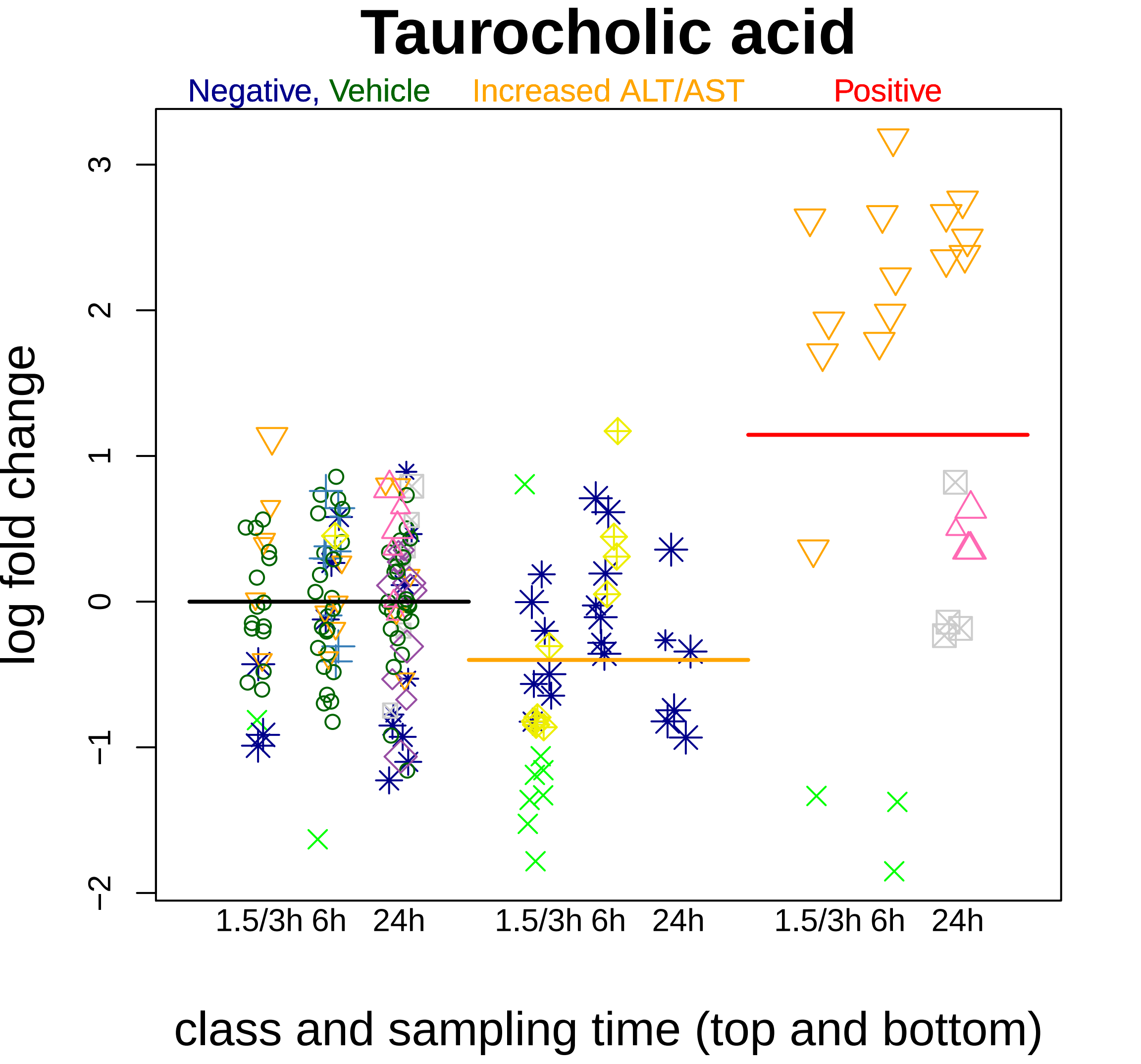
Taurocholic acid
Negative, Vehicle
Increased ALT/AST
Positive
log fold change
3
2
1
0
−1
−2
1.5/3h 6h 24h
1.5/3h 6h 24h
1.5/3h 6h 24h
class and sampling time (top and bottom)

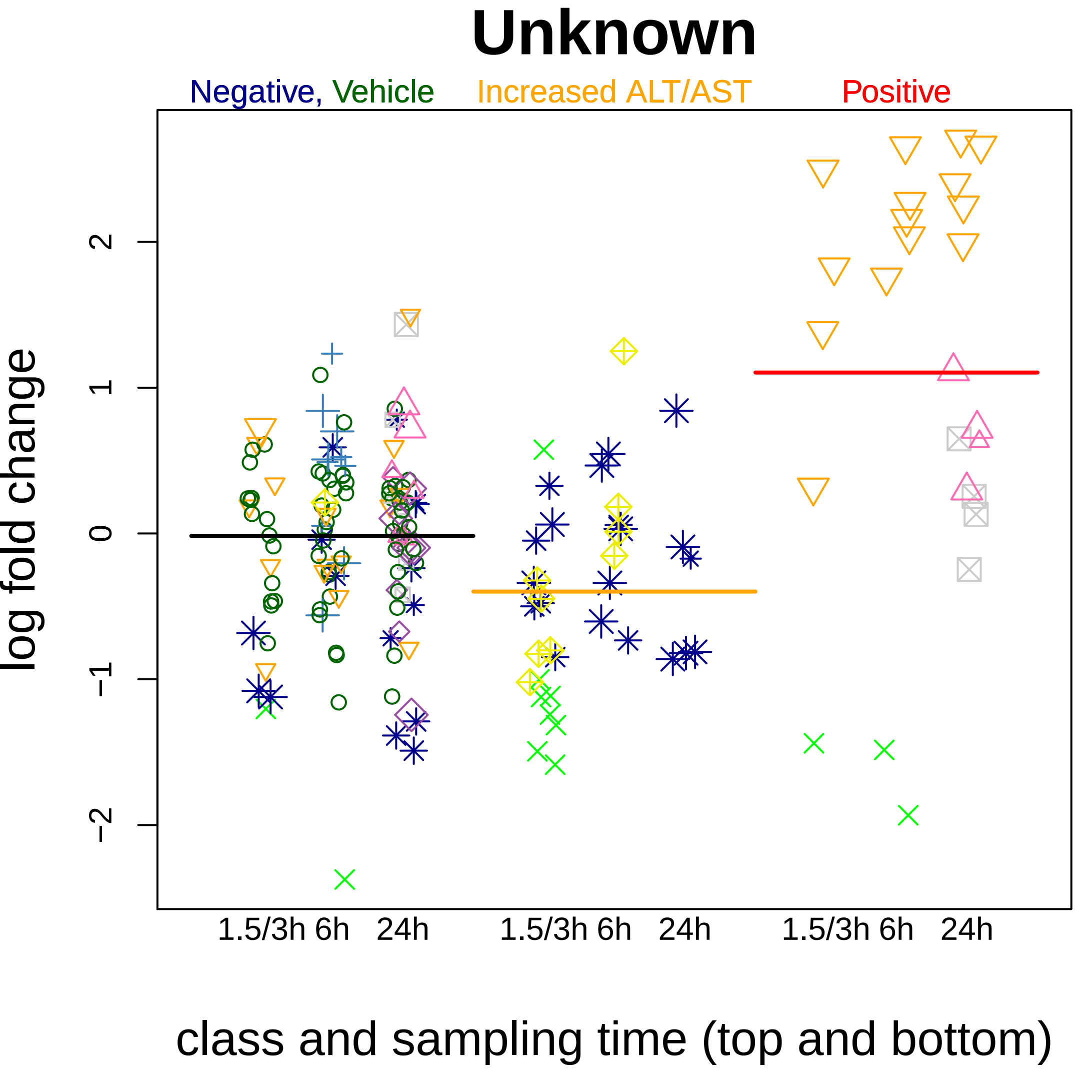
Unknown
Negative, Vehicle
Increased ALT/AST
Positive
log fold change
2
1
0
−1
−2
1.5/3h 6h
24h
1.5/3h 6h
24h
1.5/3h 6h
24h
class and sampling time (top and bottom)

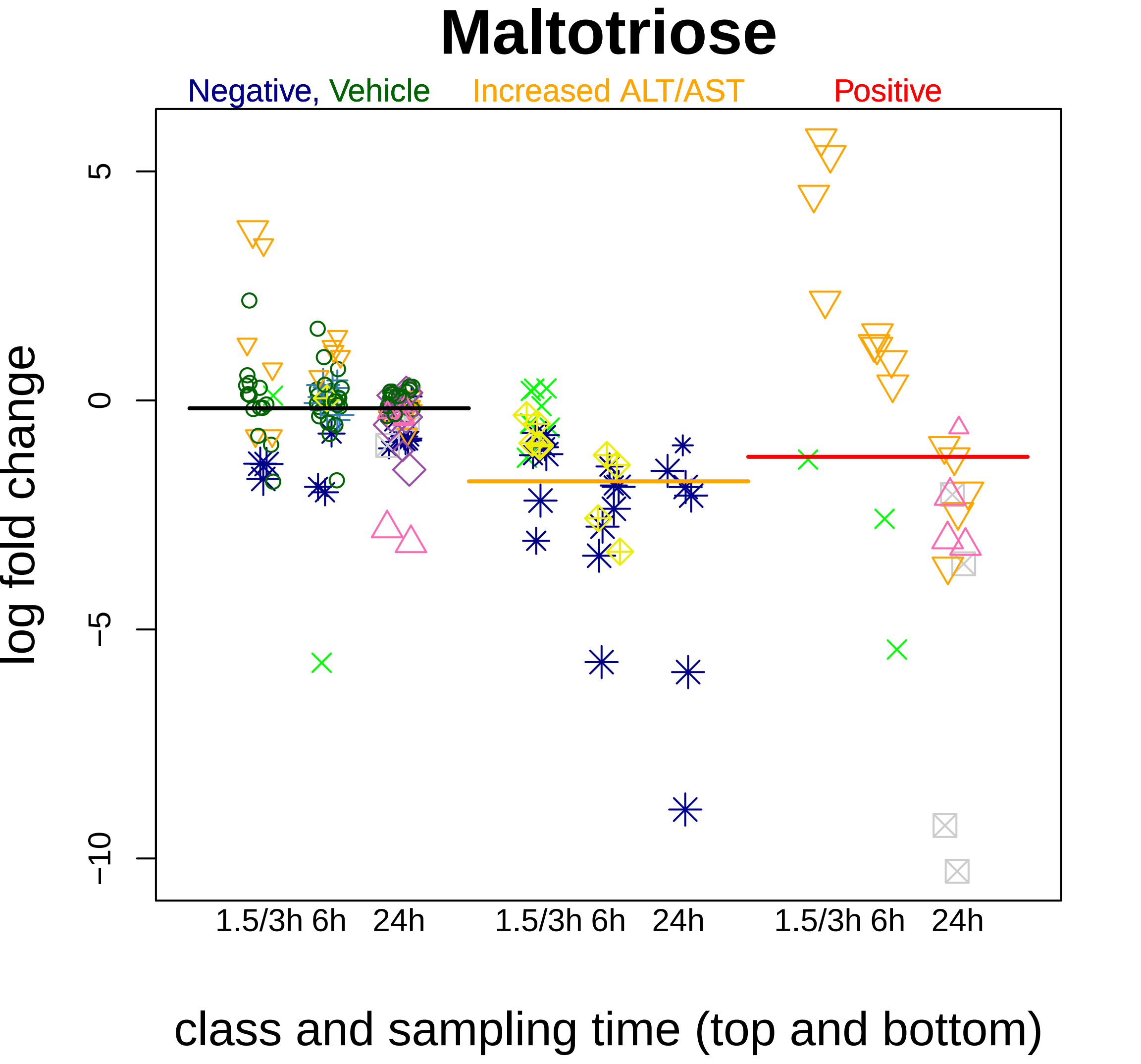
Maltotriose
Negative, Vehicle
Increased ALT/AST
Positive
log fold change
5
0
−5
−10
1.5/3h 6h 24h
1.5/3h 6h 24h
1.5/3h 6h 24h
class and sampling time (top and bottom)
