# Supplementary material for: Identification of Metabolites, Clinical Chemistry Markers and Transcripts Associated with Hepatotoxicity
Source: PLoS One. 2014 May 16;9(5):e97249. doi: 10.1371/journal.pone.0097249 (PMC4023975; doi:10.1371/journal.pone.0097249)
Supplement: Figure S6 — Transcripts in Liver Tissue as Listed in Table 8. The fold-change of each sample is calculated with respect to the matching control group (vehicle treatment). The mean of each class is shown as horizontal line (black: negative including controls, yellow: increased in ALT or AST, red: positive). The size of the treatment symbols increase with dose. The time points are resolved by aligning the symbols in columns. (PDF) [file pone.0097249.s006.pdf]

Treatments

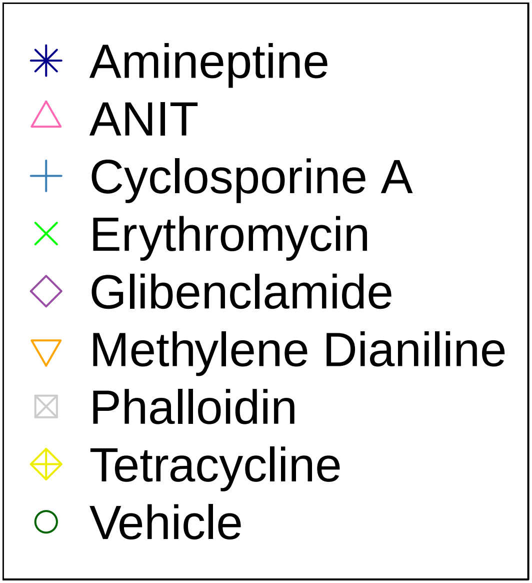
Amineptine
ANIT
Cyclosporine A
Erythromycin
Glibenclamide
Methylene Dianiline
Phalloidin
Tetracycline
Vehicle

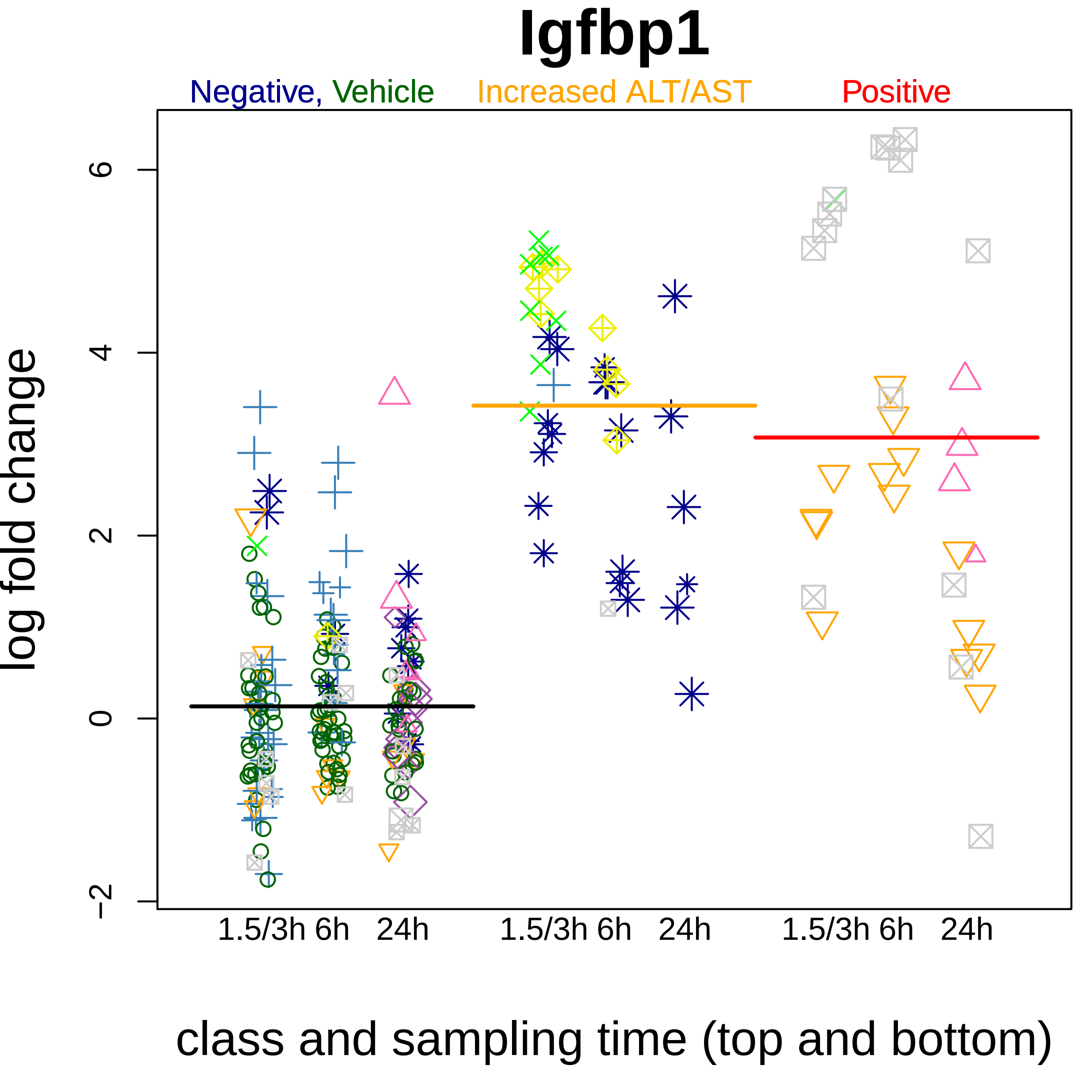
Igfbp1
Negative, Vehicle
Increased ALT/AST
Positive
log fold change
−2
0
2
4
6
1.5/3h 6h 24h
1.5/3h 6h 24h
1.5/3h 6h 24h
class and sampling time (top and bottom)

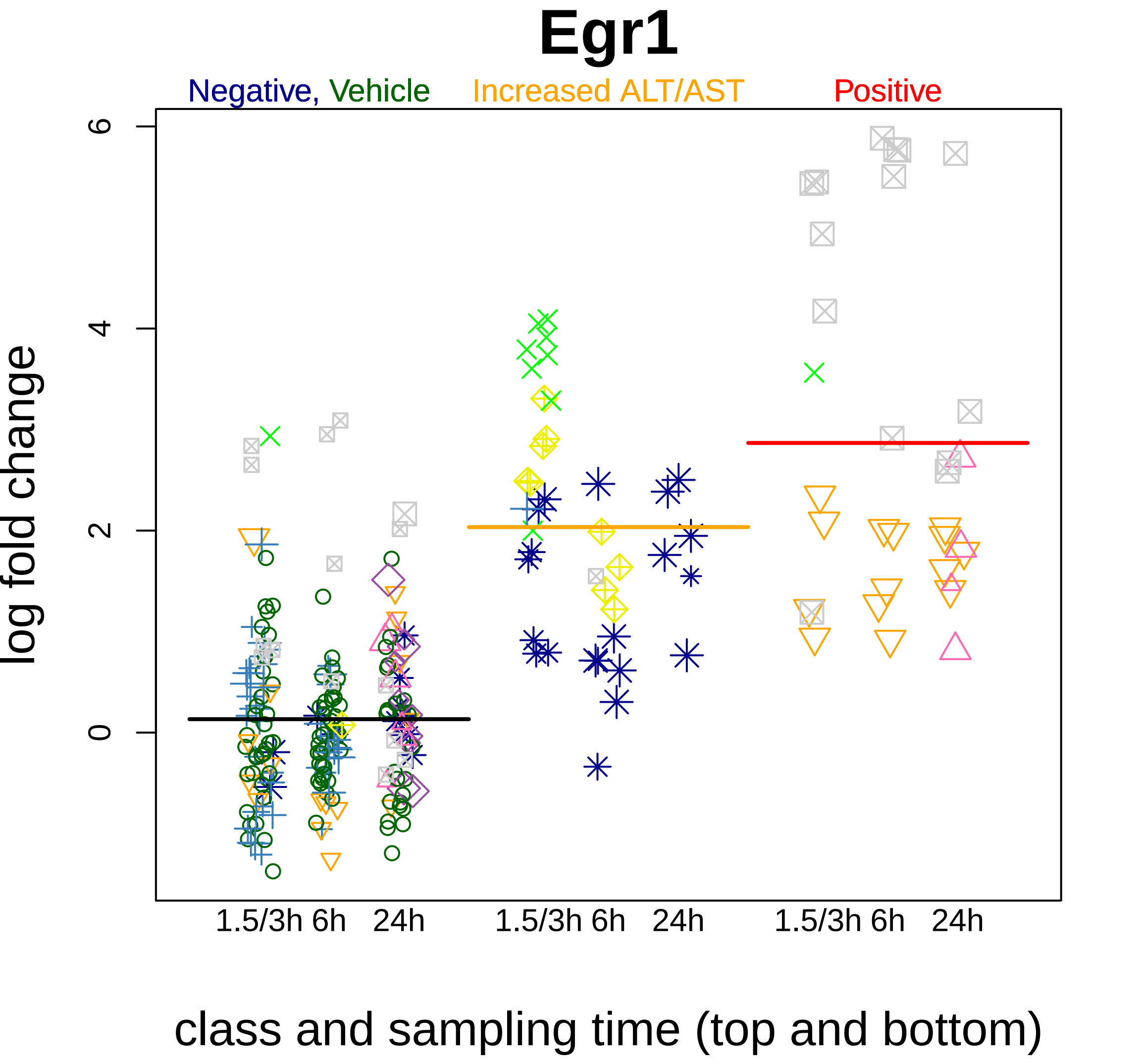

Egr1
Negative, Vehicle
Increased ALT/AST
Positive
6
4
2
0
log fold change
1.5/3h 6h 24h
1.5/3h 6h 24h
1.5/3h 6h 24h
class and sampling time (top and bottom)

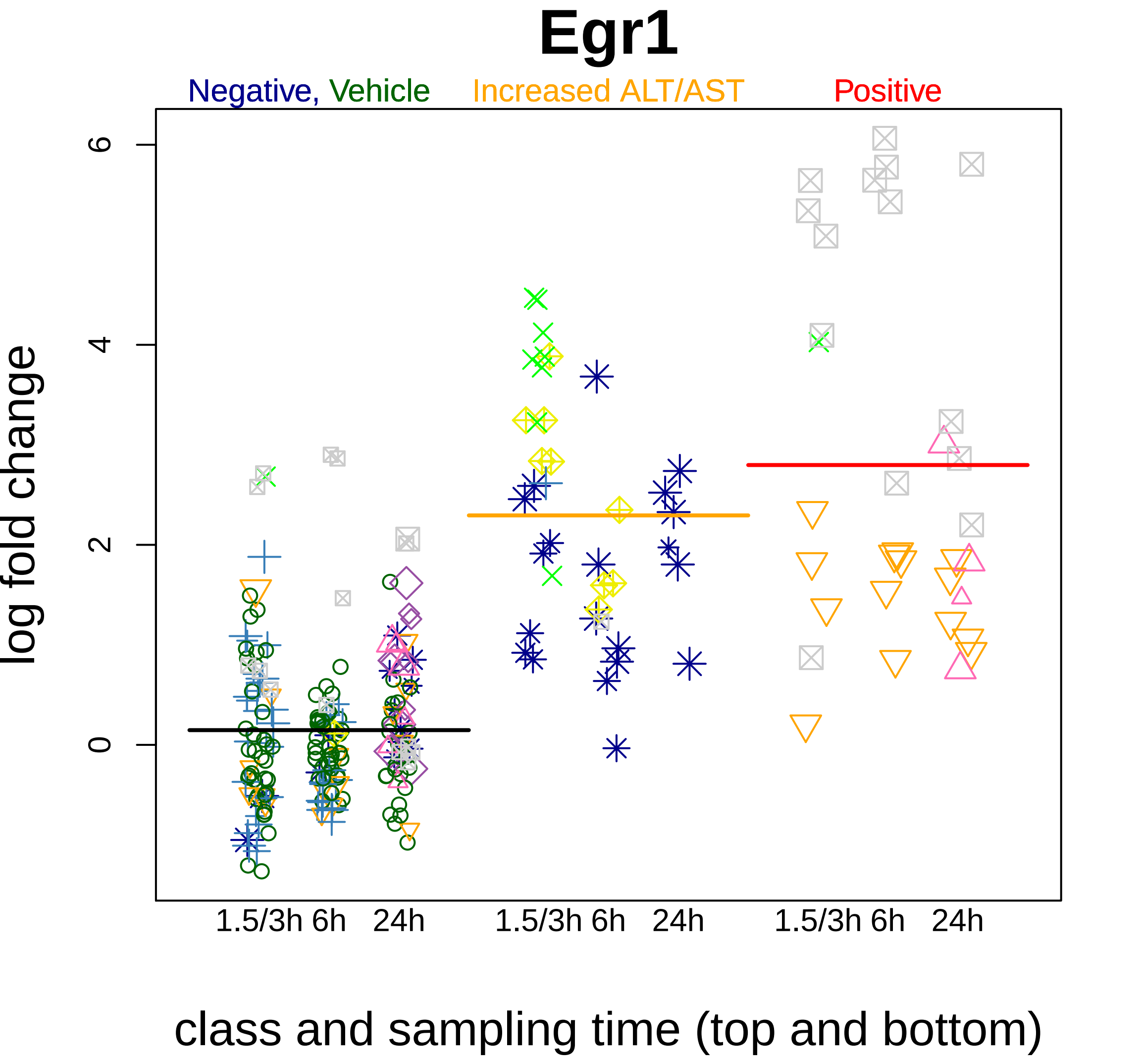

Egr1
Negative, Vehicle
Increased ALT/AST
Positive
log fold change
6
4
2
0
1.5/3h 6h 24h
1.5/3h 6h 24h
1.5/3h 6h 24h
class and sampling time (top and bottom)

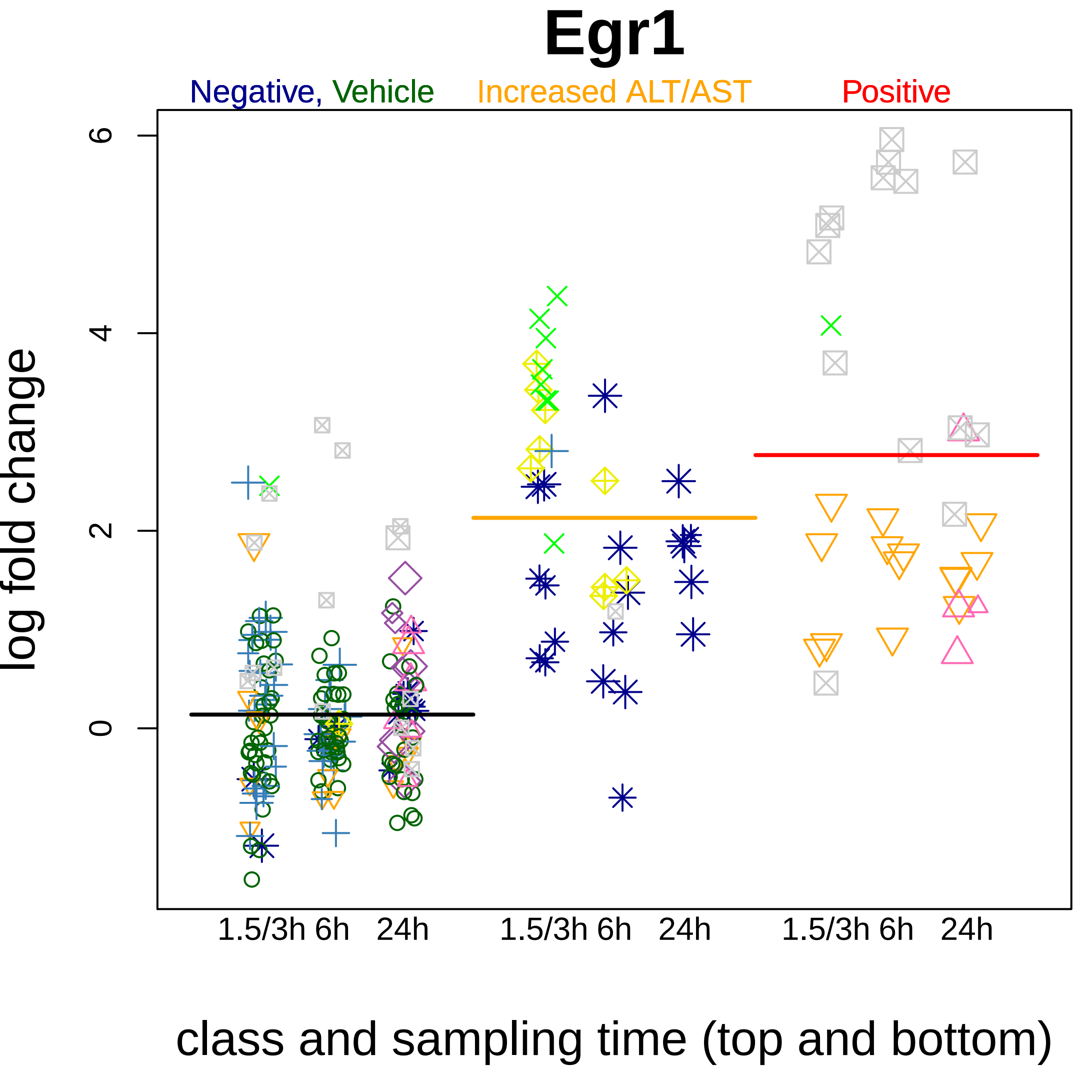
Egr1
Negative, Vehicle
Increased ALT/AST
Positive
log fold change
0
2
4
6
1.5/3h 6h 24h
1.5/3h 6h 24h
1.5/3h 6h 24h
class and sampling time (top and bottom)

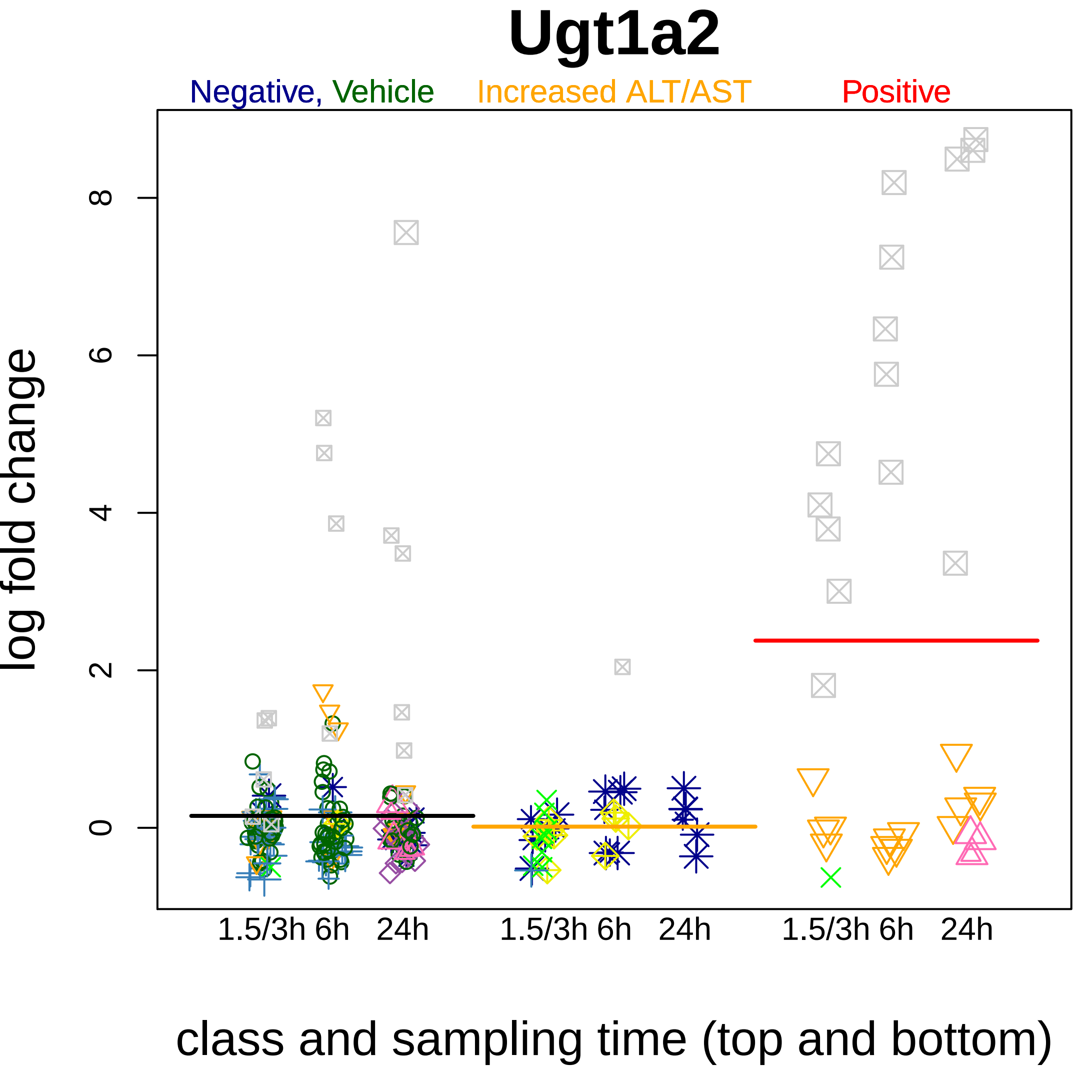

Ugt1a2
Negative, Vehicle
Increased ALT/AST
Positive
log fold change
0
2
4
6
8
1.5/3h 6h 24h
1.5/3h 6h 24h
1.5/3h 6h 24h
class and sampling time (top and bottom)

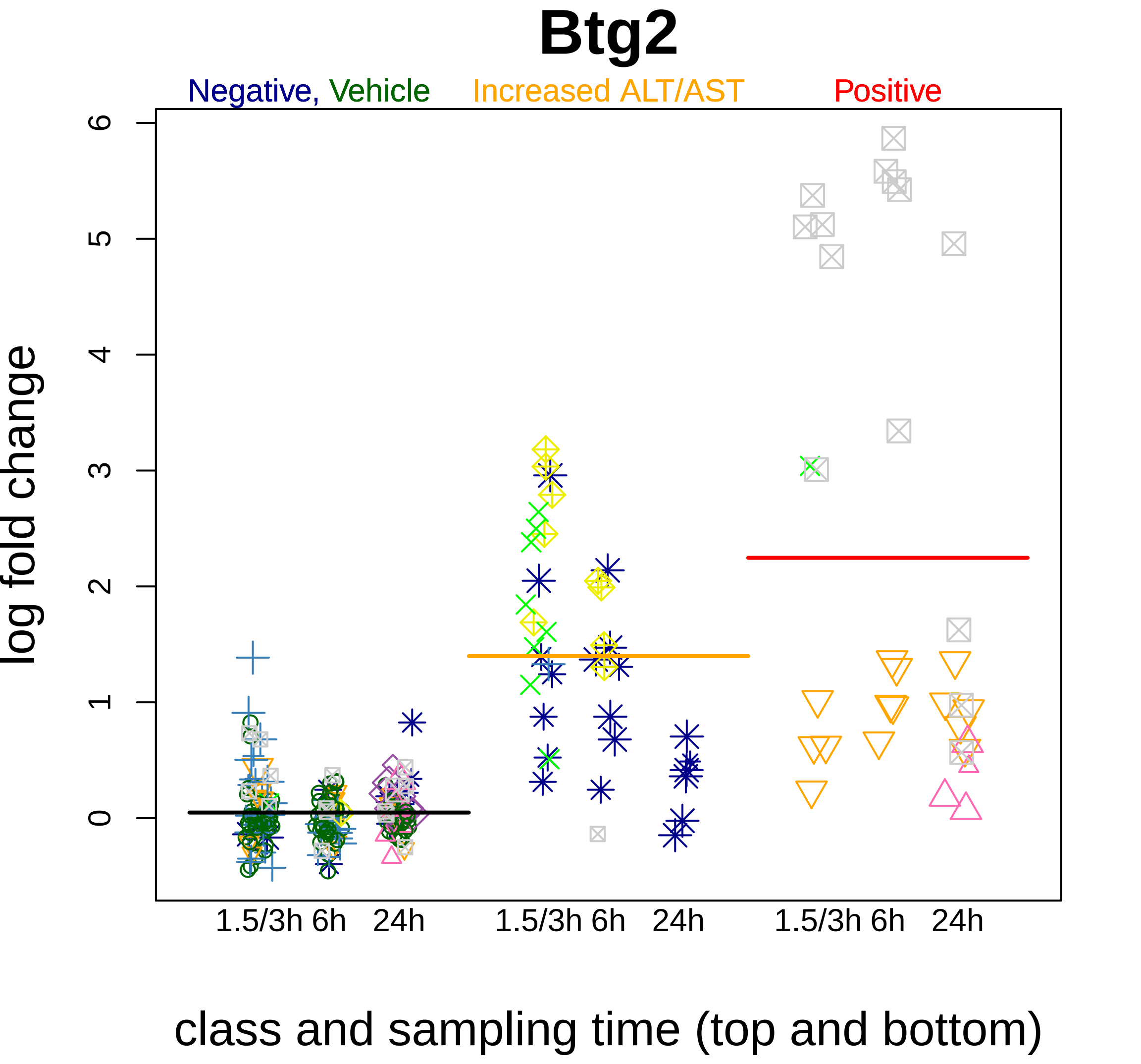
Btg2
Negative, Vehicle
Increased ALT/AST
Positive
log fold change
0
1
2
3
4
5
6
1.5/3h 6h 24h
1.5/3h 6h 24h
1.5/3h 6h 24h
class and sampling time (top and bottom)

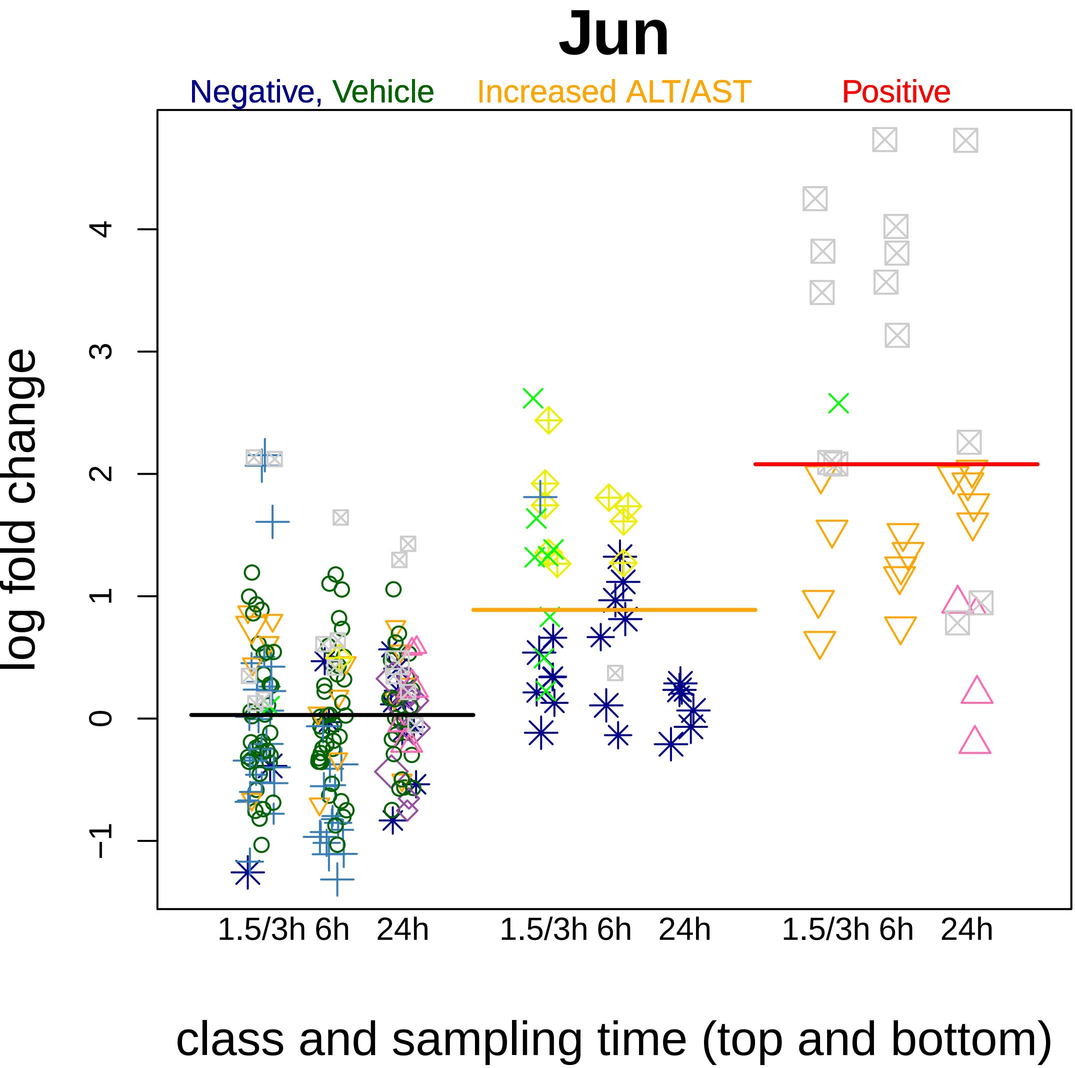
Jun
Negative, Vehicle
Increased ALT/AST
Positive
log fold change
−1
0
1
2
3
4
1.5/3h 6h 24h
1.5/3h 6h 24h
1.5/3h 6h 24h
class and sampling time (top and bottom)

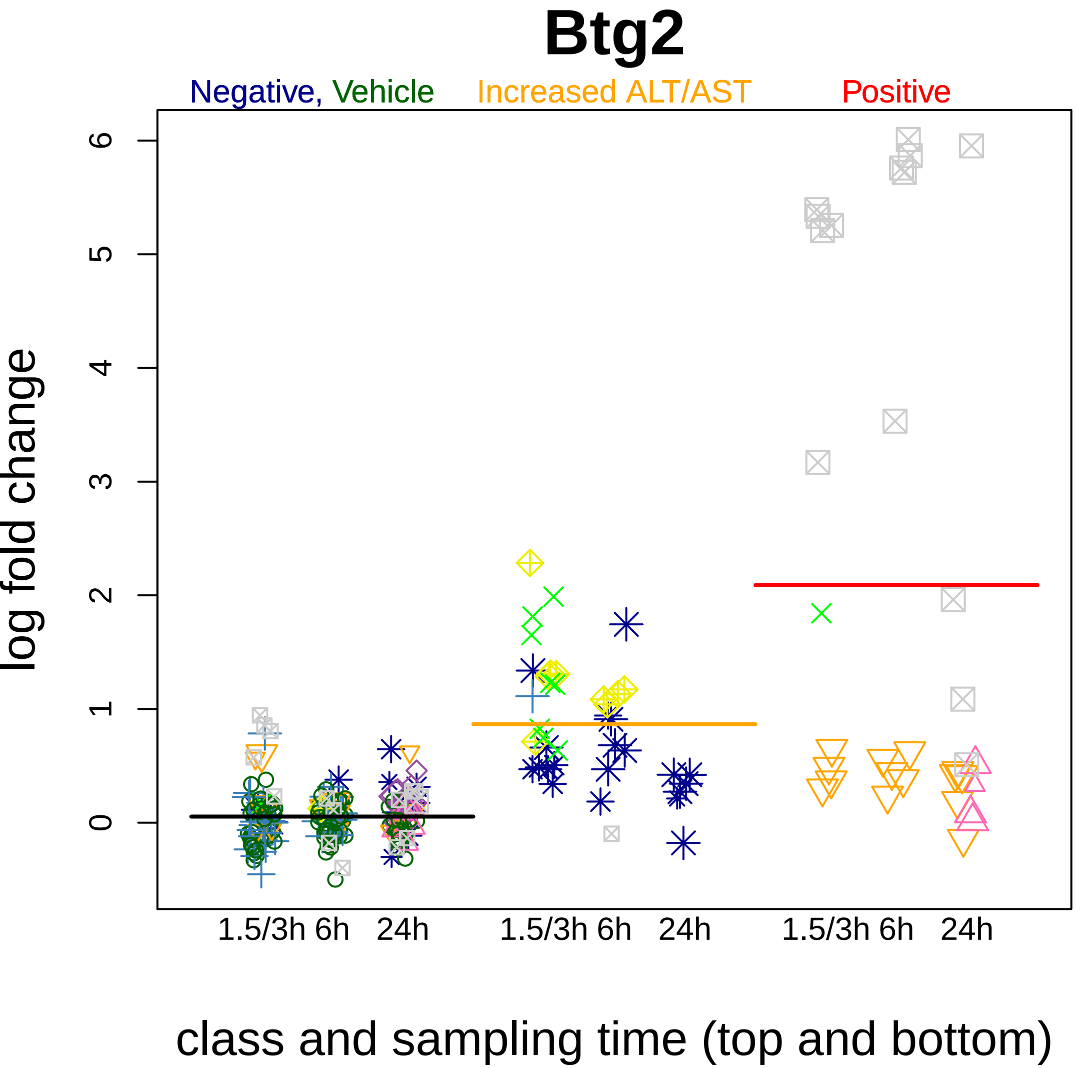

Btg2
Negative, Vehicle
Increased ALT/AST
Positive
log fold change
0
1
2
3
4
5
6
1.5/3h 6h 24h
1.5/3h 6h 24h
1.5/3h 6h 24h
class and sampling time (top and bottom)

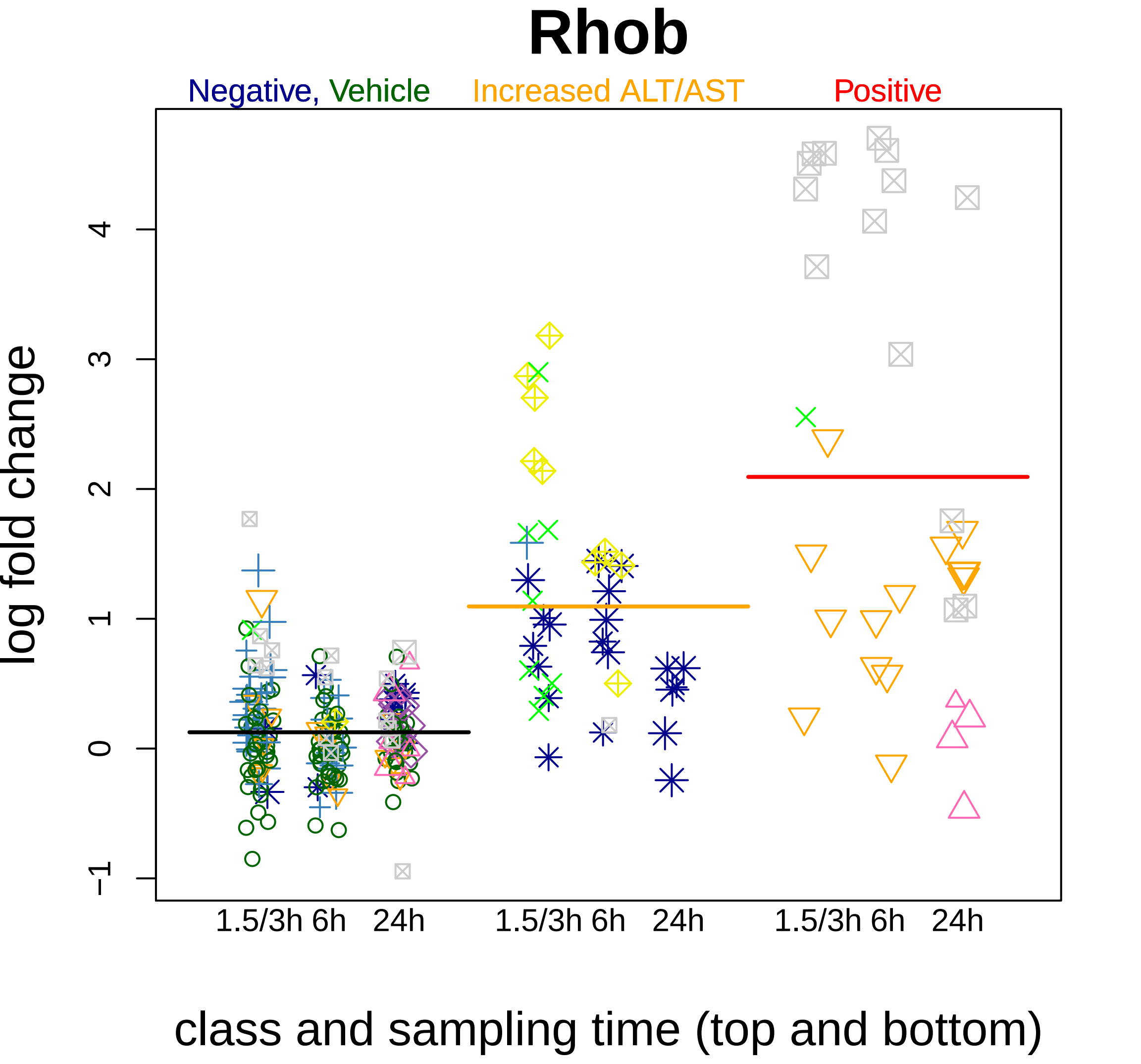
Rhob
Negative, Vehicle
Increased ALT/AST
Positive
log fold change
−1
0
1
2
3
4
1.5/3h 6h 24h
1.5/3h 6h 24h
1.5/3h 6h 24h
class and sampling time (top and bottom)

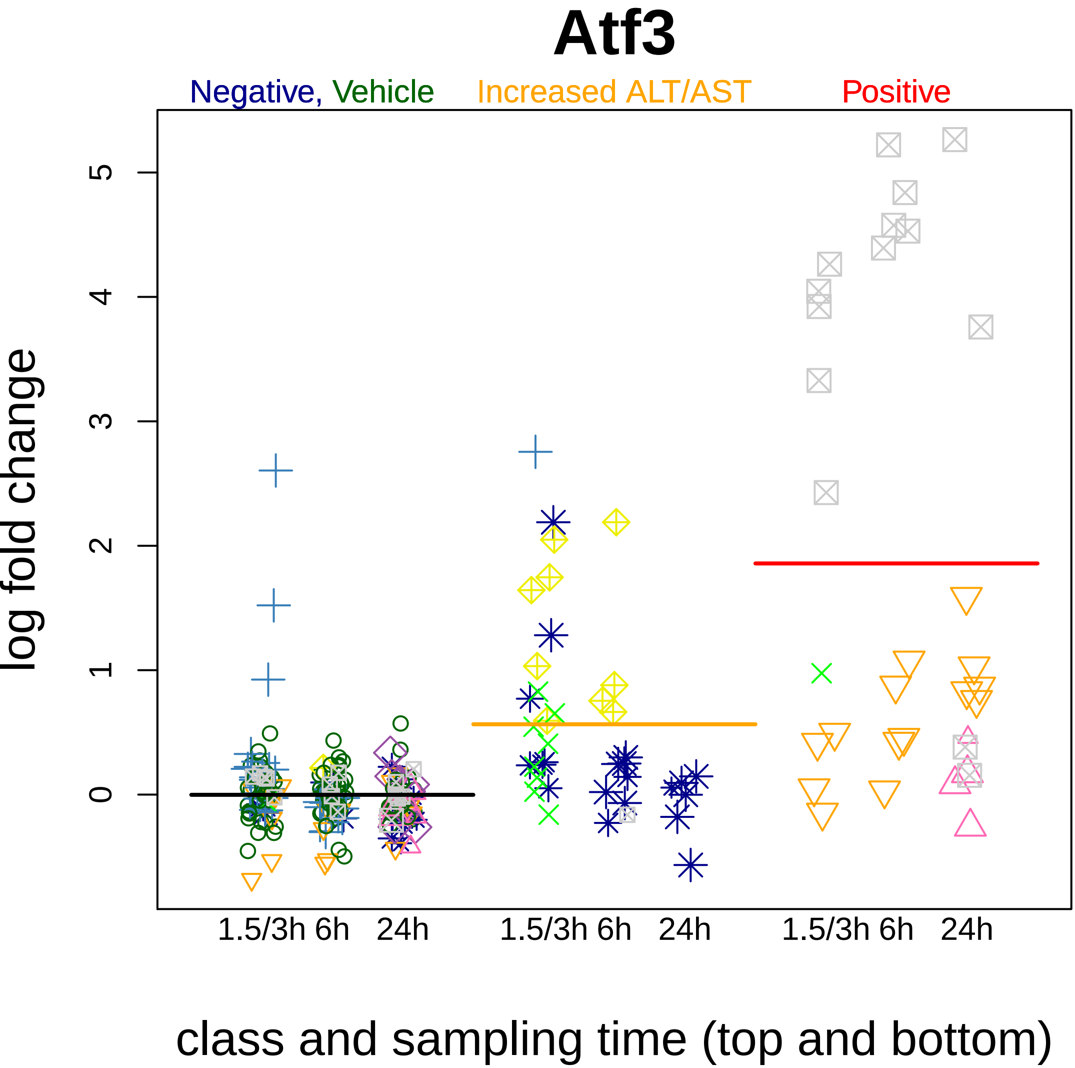

Atf3
Negative, Vehicle
Increased ALT/AST
Positive
log fold change
0
1
2
3
4
5
1.5/3h 6h 24h
1.5/3h 6h 24h
1.5/3h 6h 24h
class and sampling time (top and bottom)

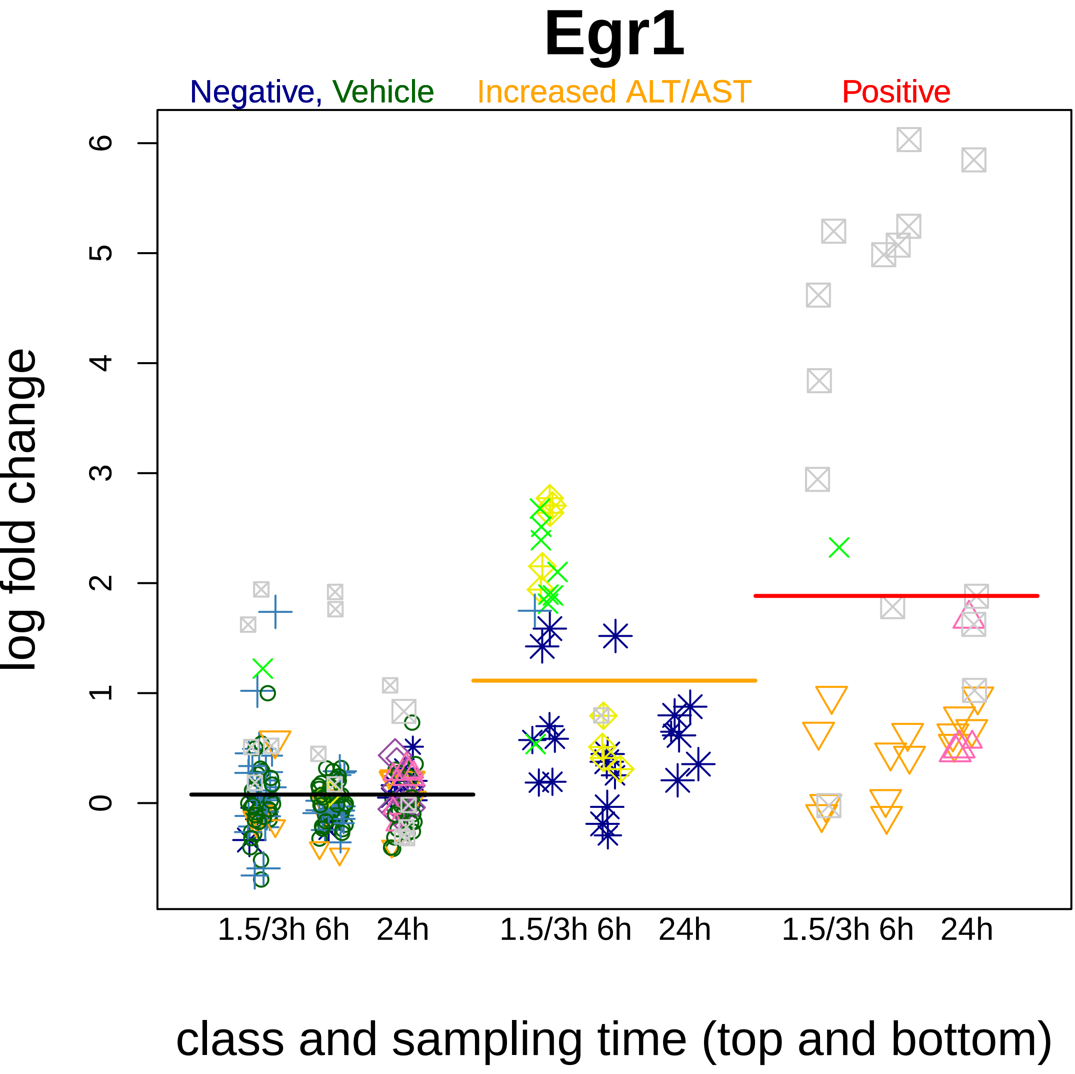

Egr1
Negative, Vehicle
Increased ALT/AST
Positive
log fold change
0
1
2
3
4
5
6
1.5/3h 6h 24h
1.5/3h 6h 24h
1.5/3h 6h 24h
class and sampling time (top and bottom)

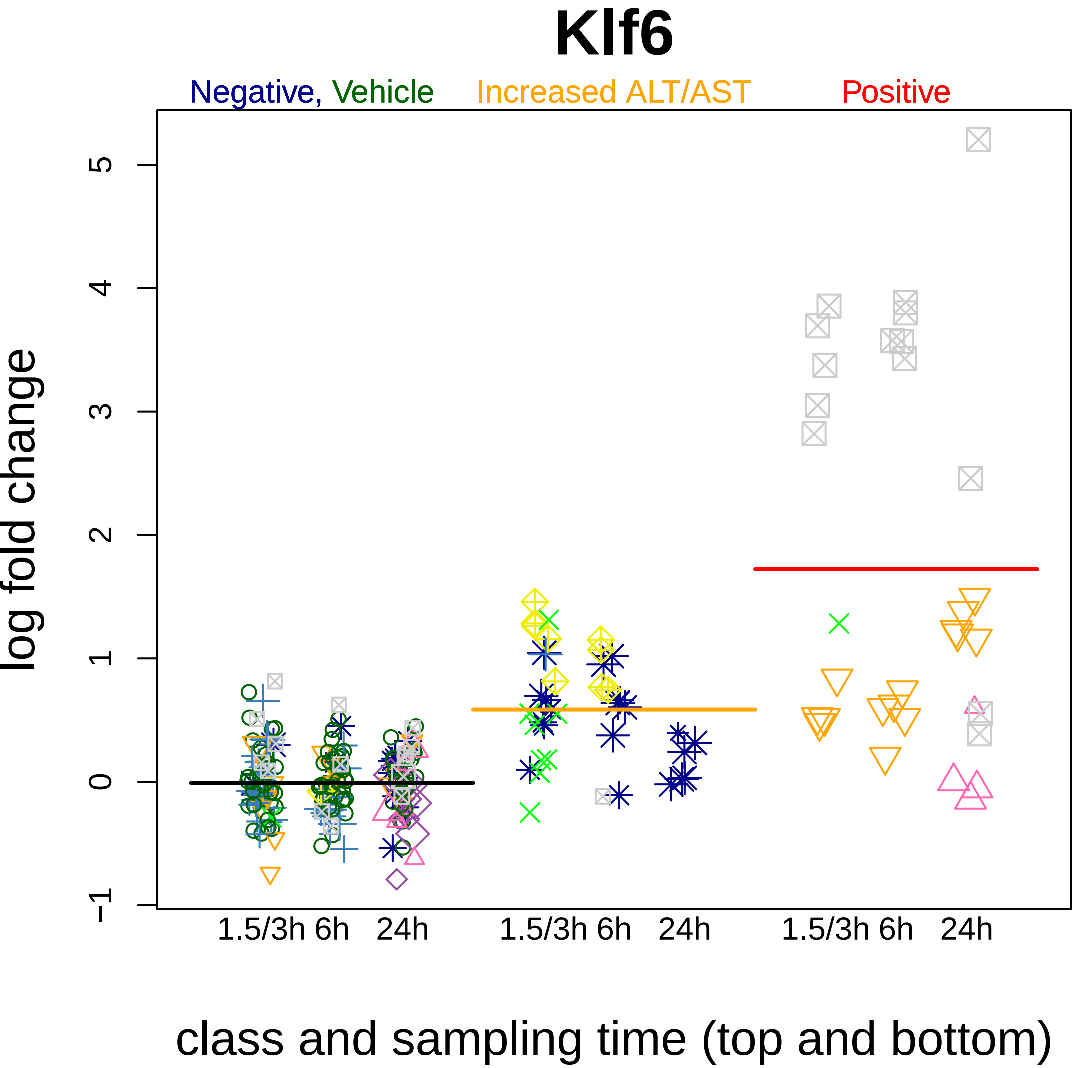
Klf6
Negative, Vehicle
Increased ALT/AST
Positive
log fold change
5
4
3
2
1
0
−1
1.5/3h 6h 24h
1.5/3h 6h 24h
1.5/3h 6h 24h
class and sampling time (top and bottom)

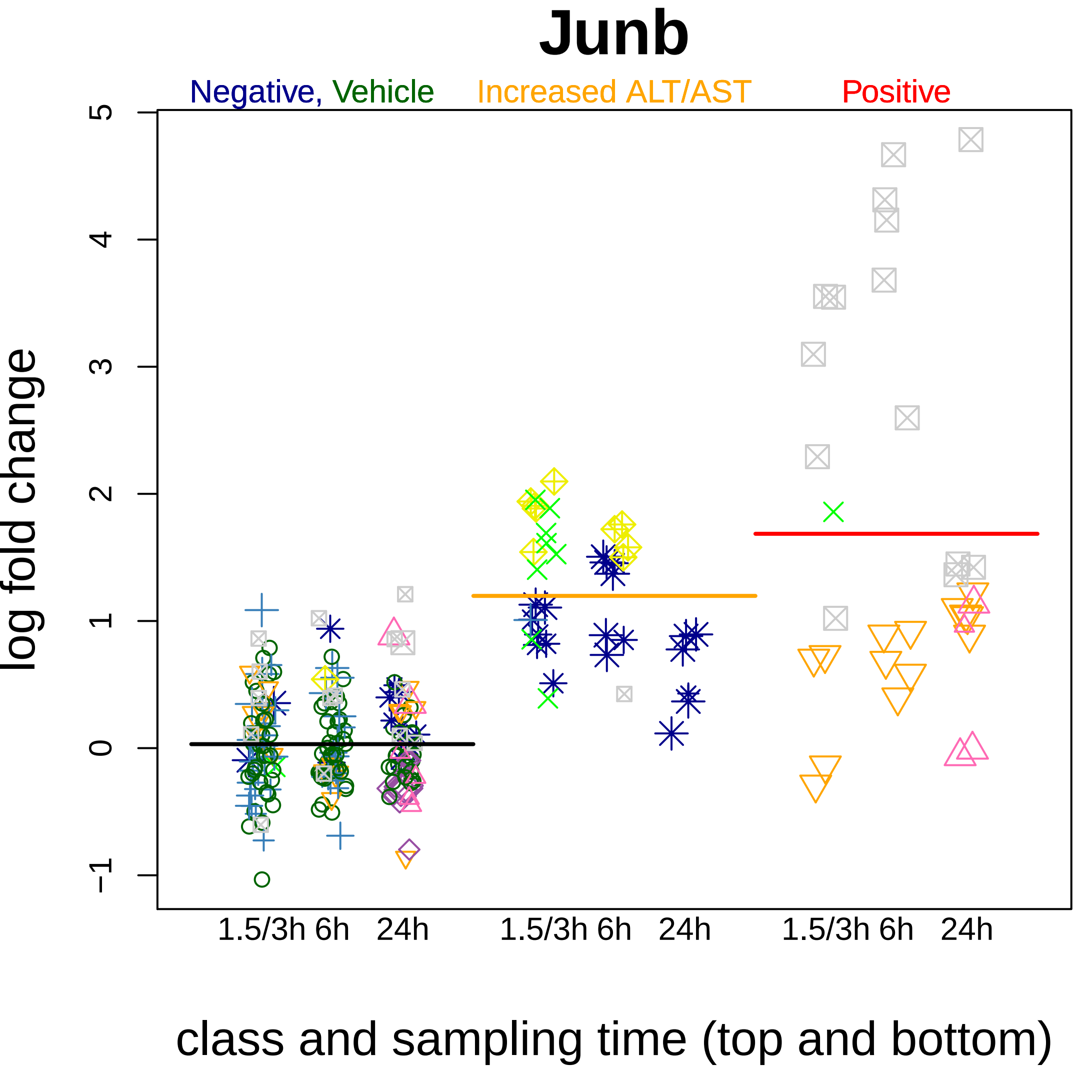
Junb
Negative, Vehicle
Increased ALT/AST
Positive
log fold change
−1
0
1
2
3
4
5
1.5/3h 6h 24h
1.5/3h 6h 24h
1.5/3h 6h 24h
class and sampling time (top and bottom)

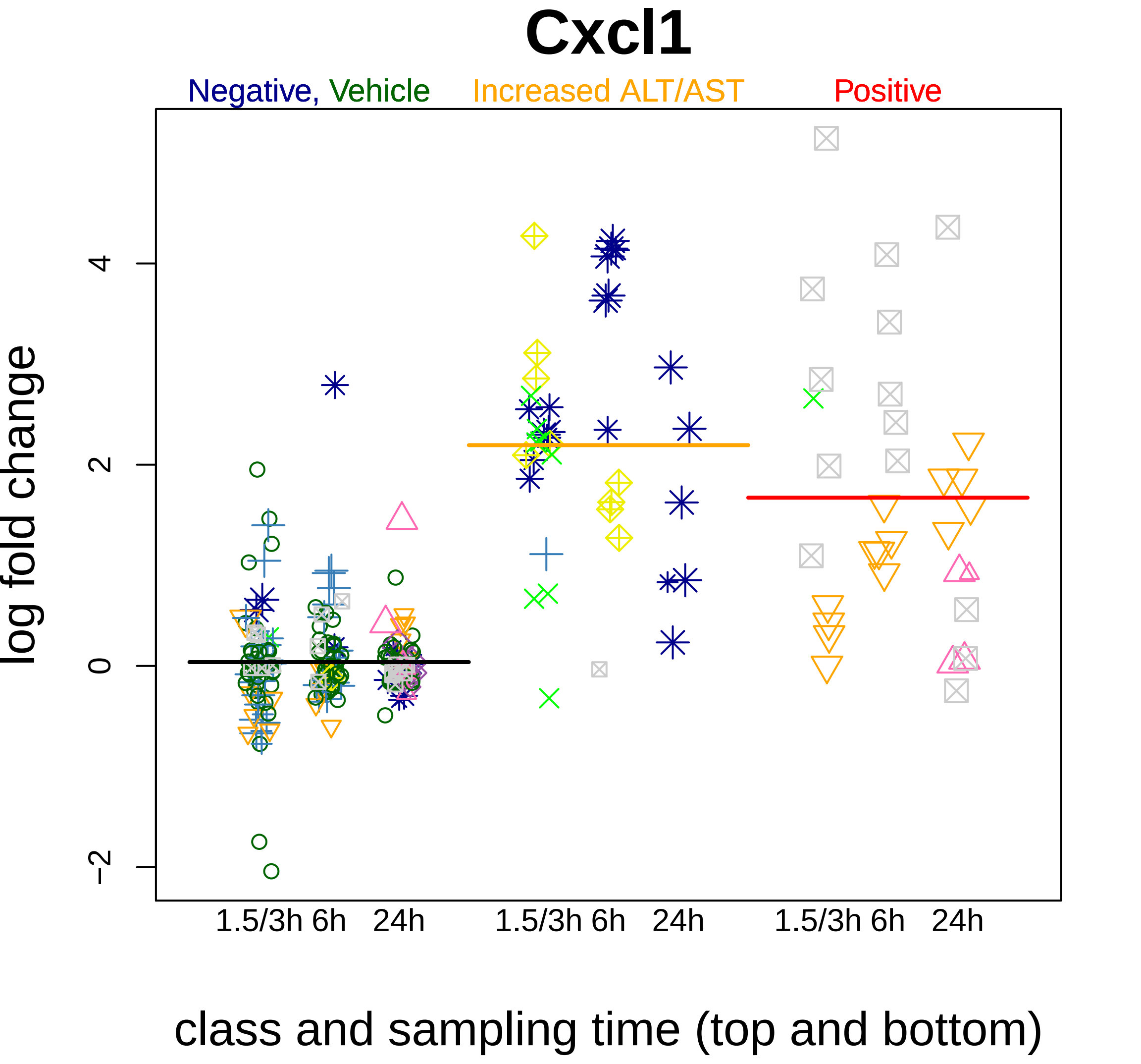
Cxcl1
Negative, Vehicle
Increased ALT/AST
Positive
log fold change
−2
0
2
4
1.5/3h 6h 24h
1.5/3h 6h 24h
1.5/3h 6h 24h
class and sampling time (top and bottom)

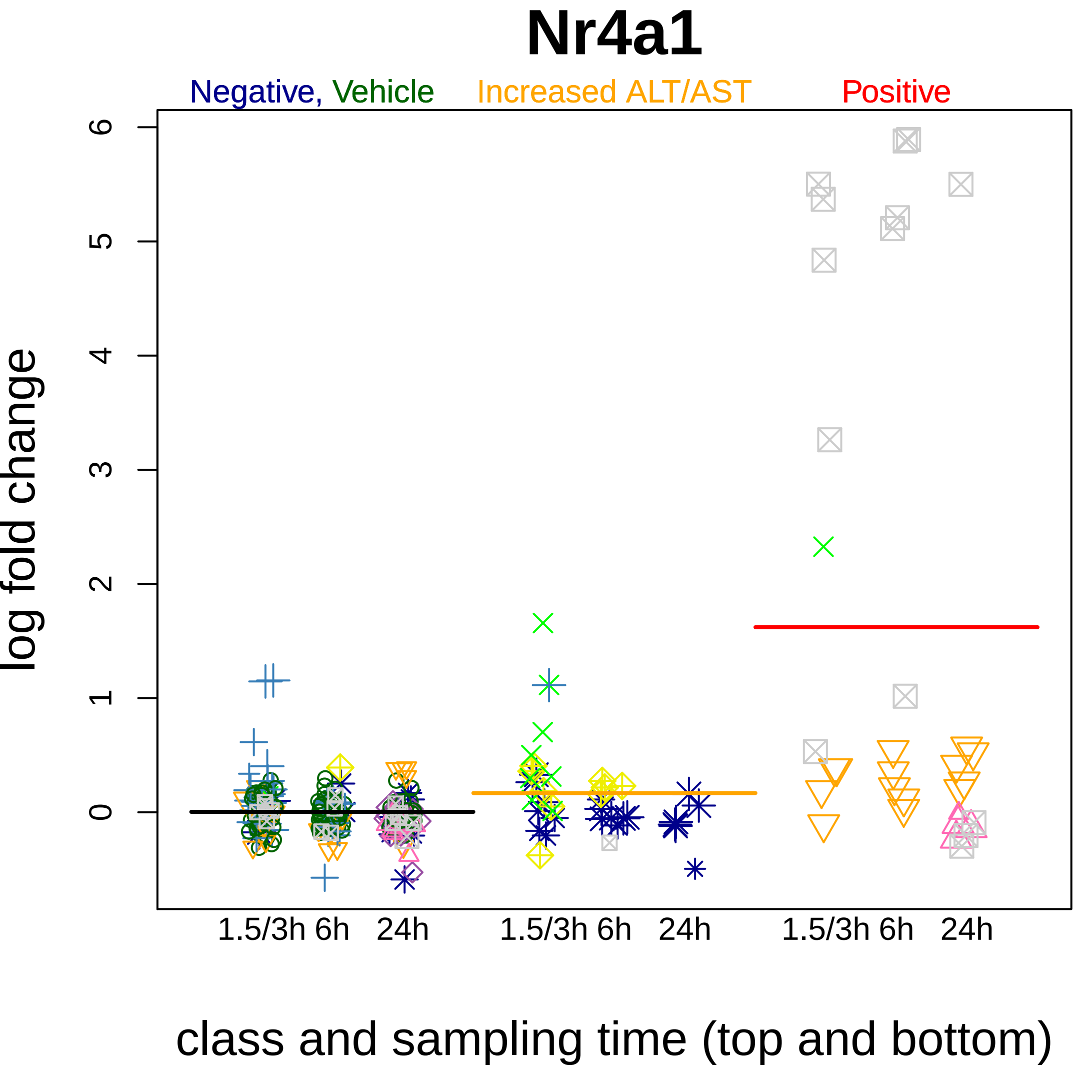

Nr4a1
Negative, Vehicle
Increased ALT/AST
Positive
log fold change
0
1
2
3
4
5
6
1.5/3h 6h 24h
1.5/3h 6h 24h
1.5/3h 6h 24h
class and sampling time (top and bottom)

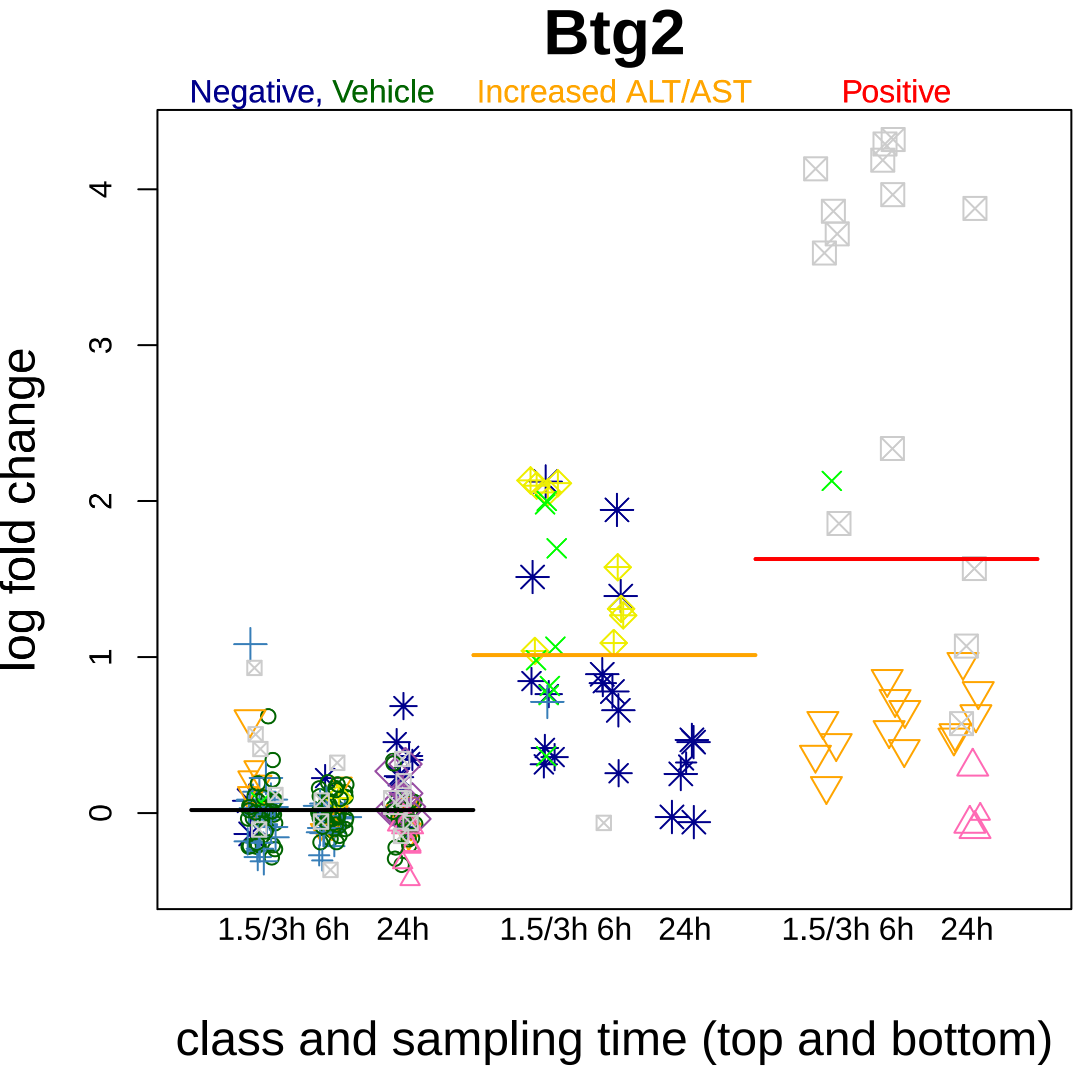

Btg2
Negative, Vehicle
Increased ALT/AST
Positive
log fold change
0
1
2
3
4
1.5/3h 6h 24h
1.5/3h 6h 24h
1.5/3h 6h 24h
class and sampling time (top and bottom)

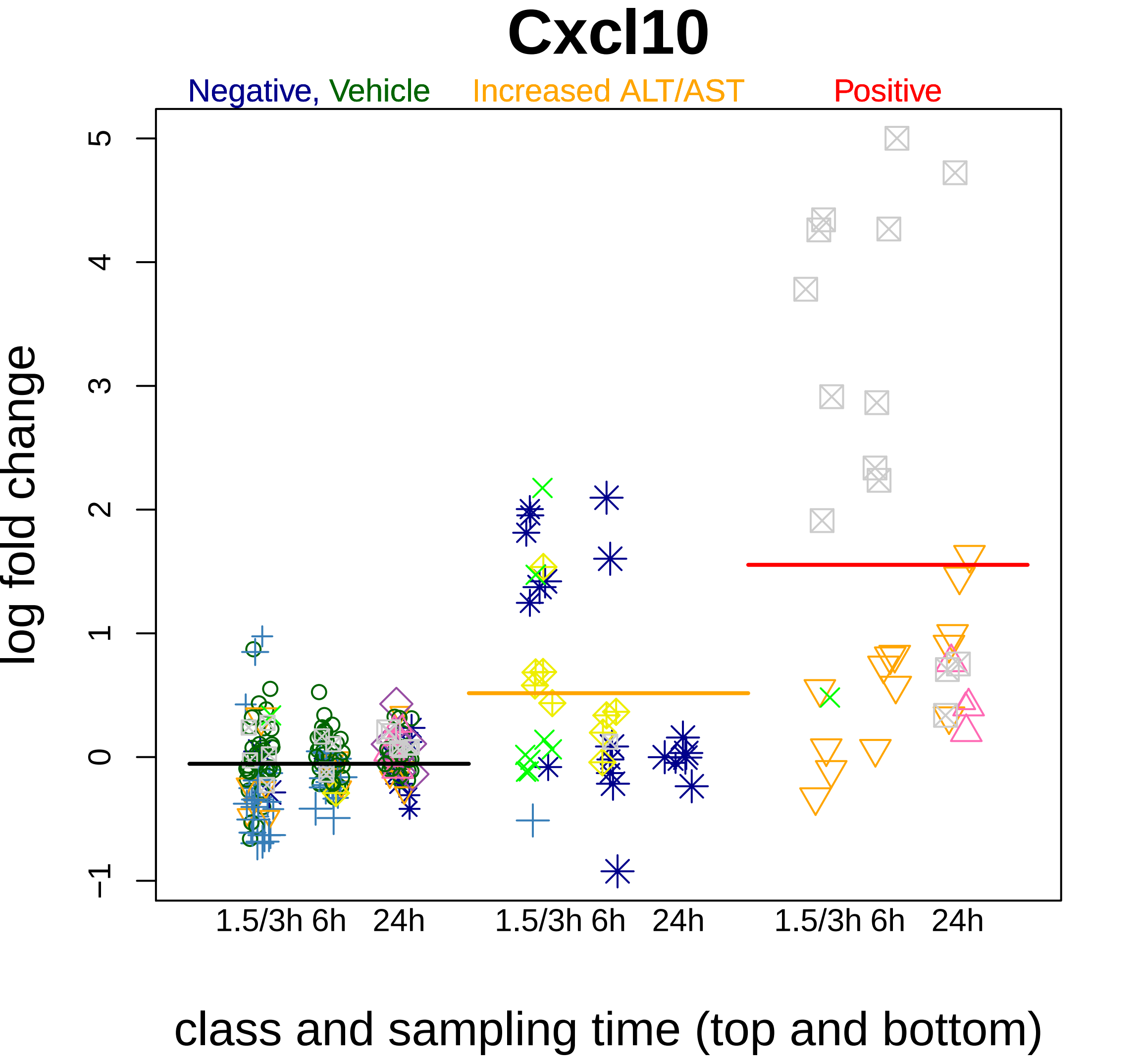

Cxcl10
Negative, Vehicle
Increased ALT/AST
Positive
log fold change
5
4
3
2
1
0
−1
1.5/3h 6h 24h
1.5/3h 6h 24h
1.5/3h 6h 24h
class and sampling time (top and bottom)

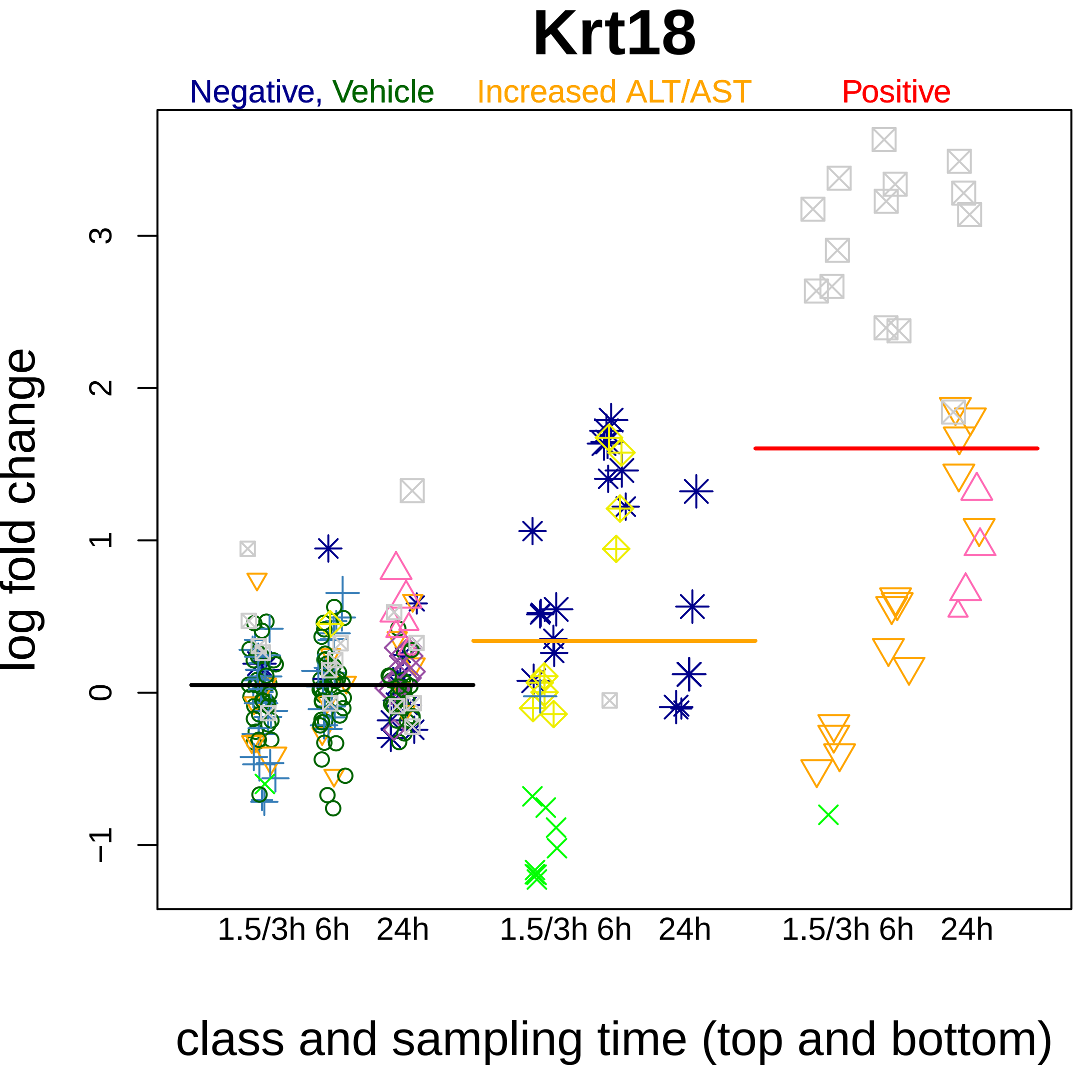
Krt18
Negative, Vehicle
Increased ALT/AST
Positive
log fold change
-1
0
1
2
3
1.5/3h 6h 24h
1.5/3h 6h 24h
1.5/3h 6h 24h
class and sampling time (top and bottom)

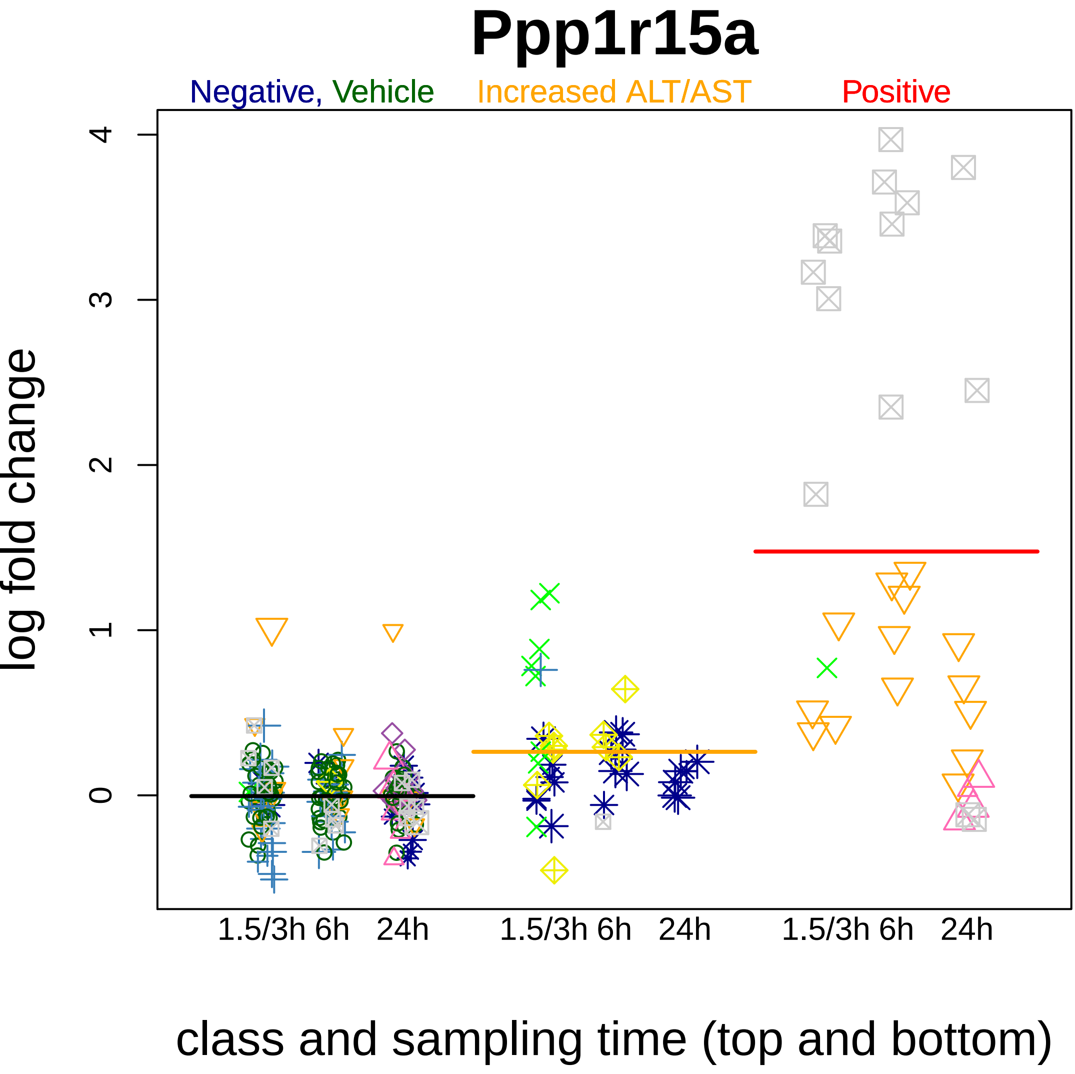
Ppp1r15a
Negative, Vehicle
Increased ALT/AST
Positive
log fold change
0
1
2
3
4
1.5/3h 6h 24h
1.5/3h 6h 24h
1.5/3h 6h 24h
class and sampling time (top and bottom)

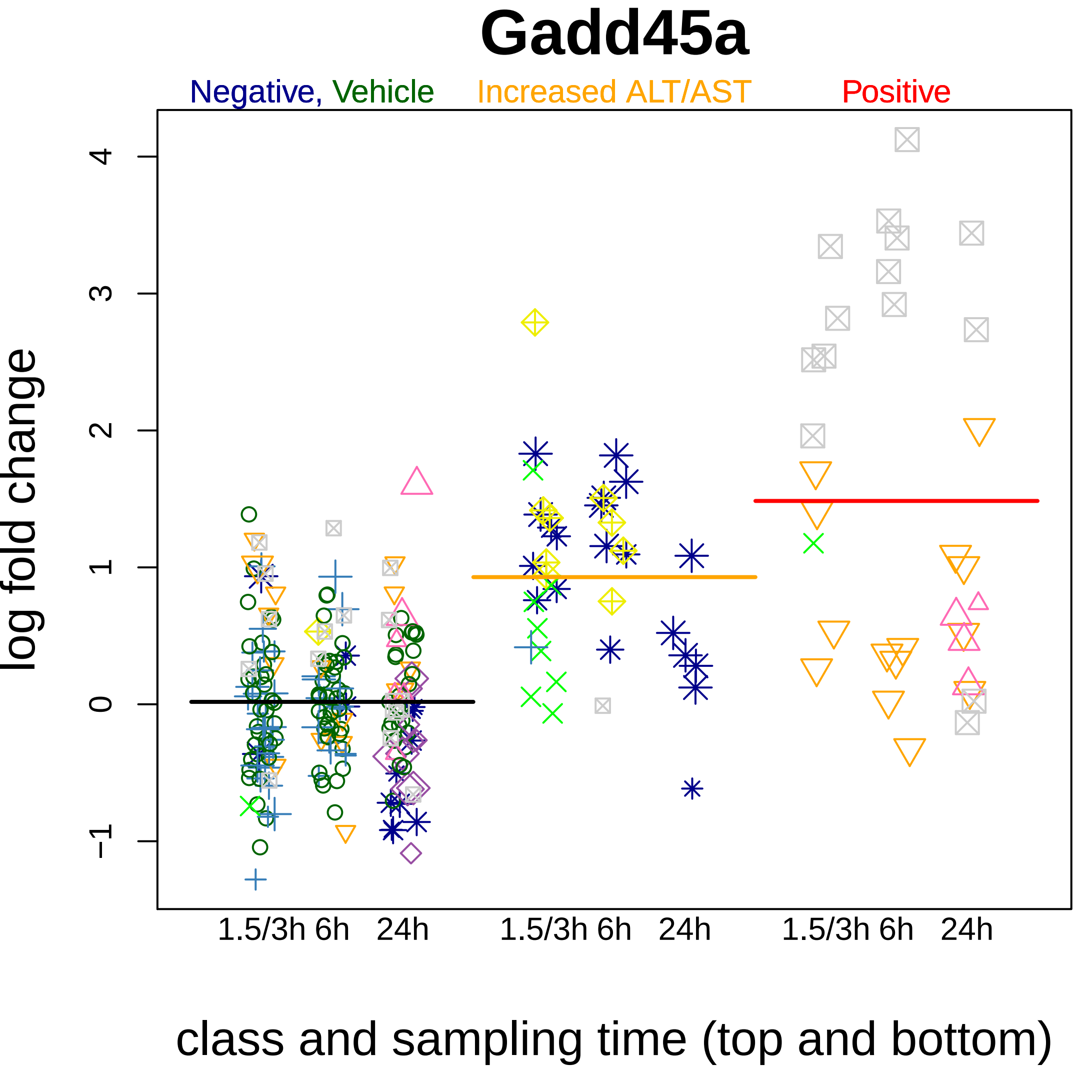

Gadd45a
Negative, Vehicle
Increased ALT/AST
Positive
log fold change
4
3
2
1
0
−1
1.5/3h 6h 24h
1.5/3h 6h 24h
1.5/3h 6h 24h
class and sampling time (top and bottom)

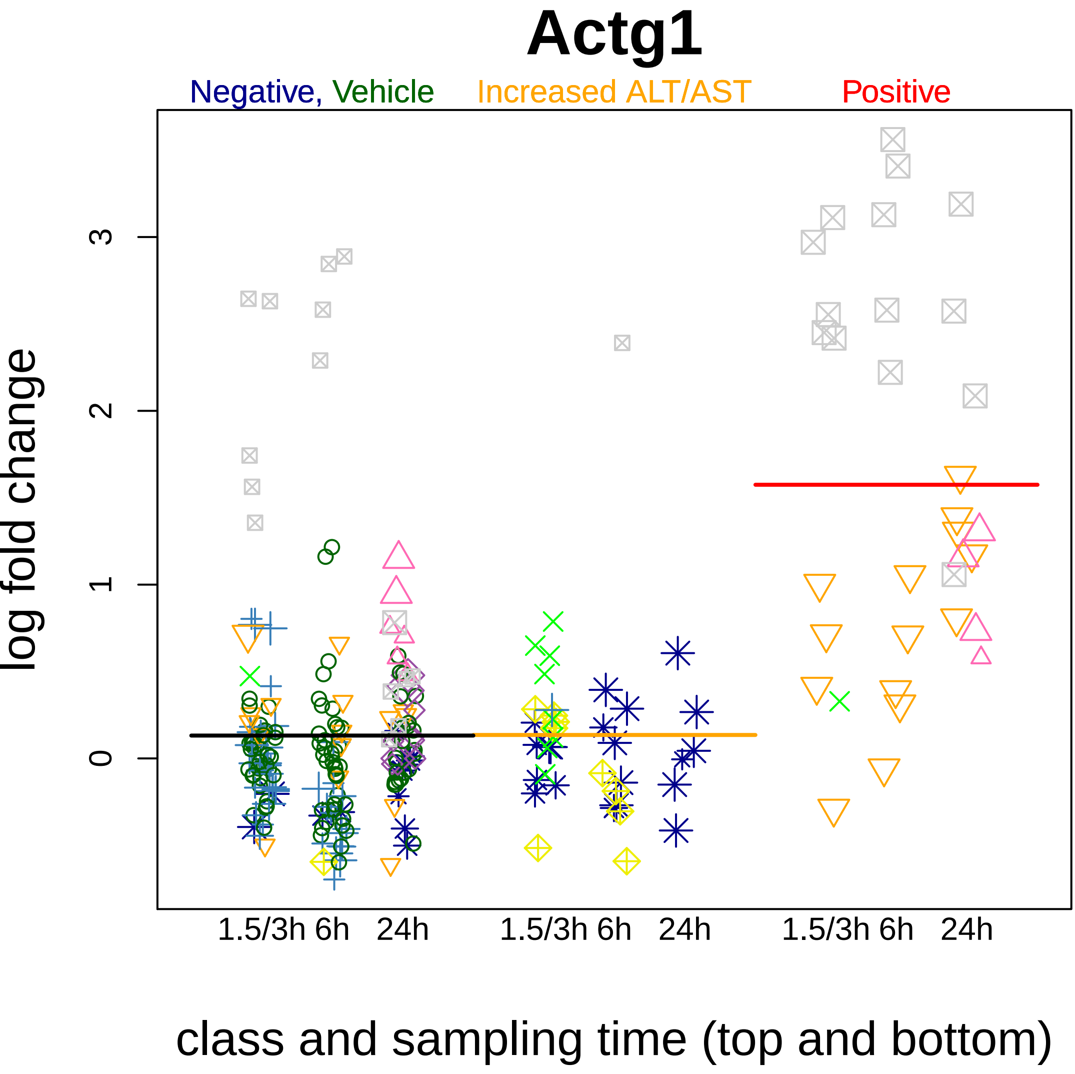

Actg1
Negative, Vehicle
Increased ALT/AST
Positive
log fold change
0
1
2
3
1.5/3h 6h 24h
1.5/3h 6h 24h
1.5/3h 6h 24h
class and sampling time (top and bottom)

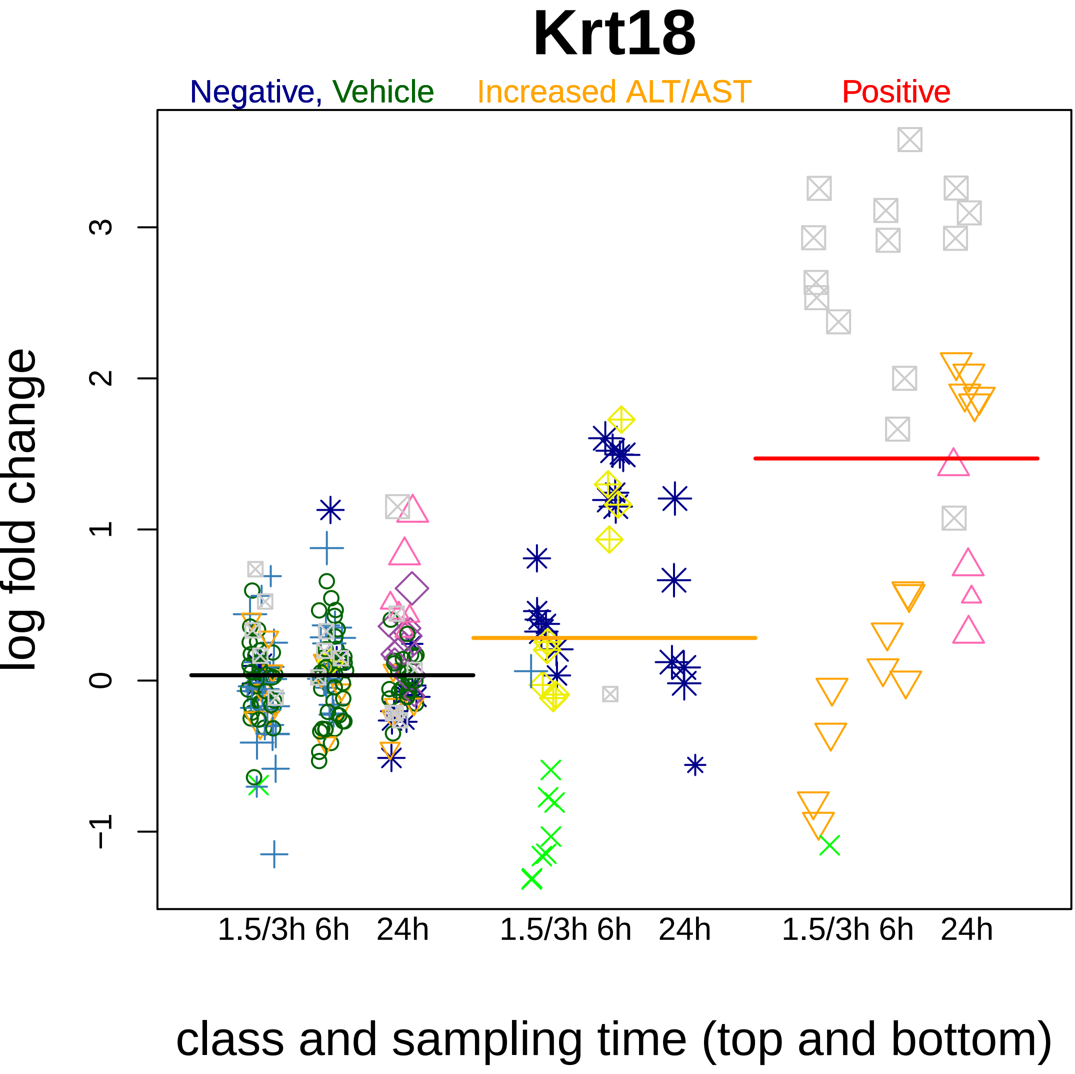
Krt18
Negative, Vehicle
Increased ALT/AST
Positive
log fold change
−1
0
1
2
3
1.5/3h 6h 24h
1.5/3h 6h 24h
1.5/3h 6h 24h
class and sampling time (top and bottom)

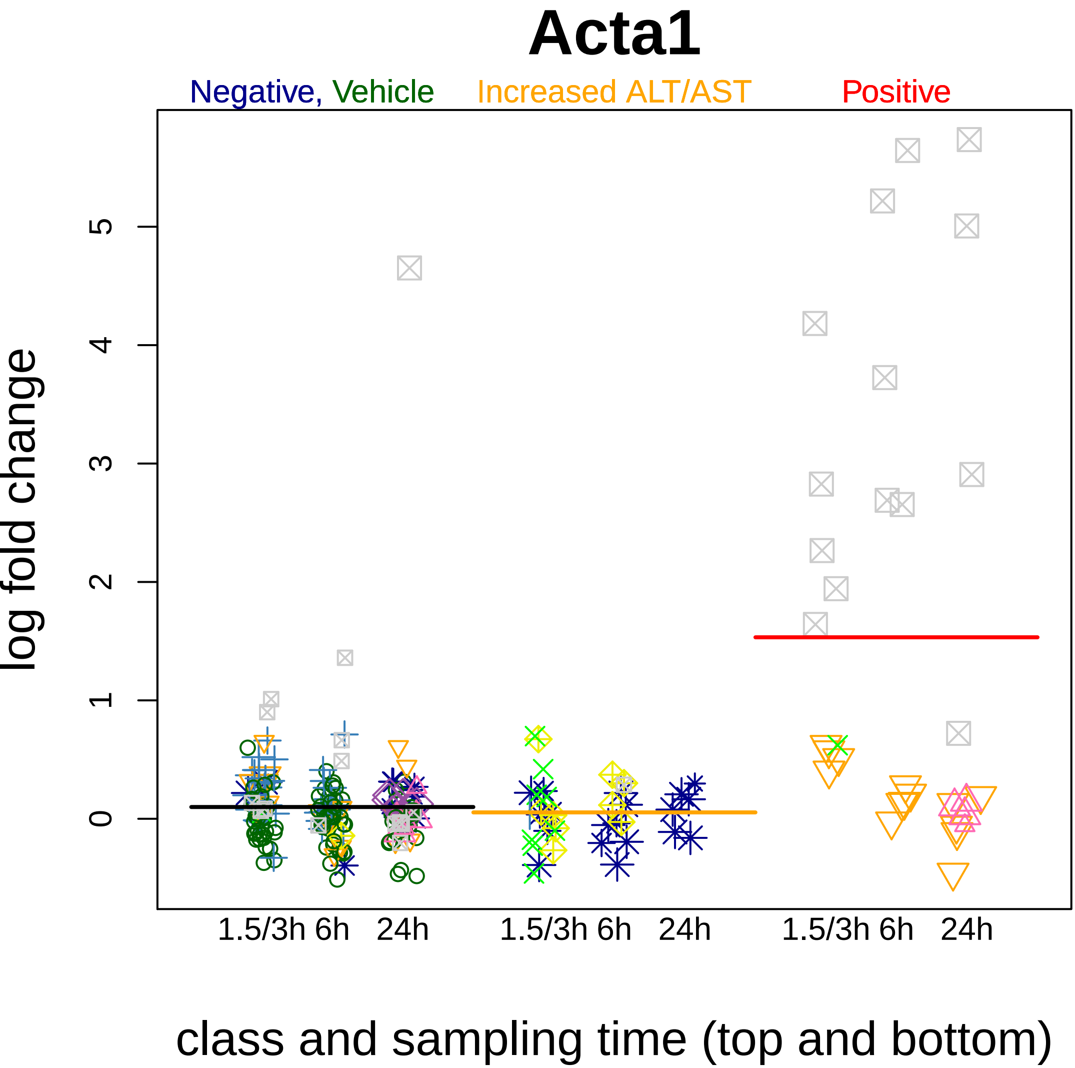

Acta1
Negative, Vehicle
Increased ALT/AST
Positive
log fold change
0
1
2
3
4
5
1.5/3h 6h 24h
1.5/3h 6h 24h
1.5/3h 6h 24h
class and sampling time (top and bottom)

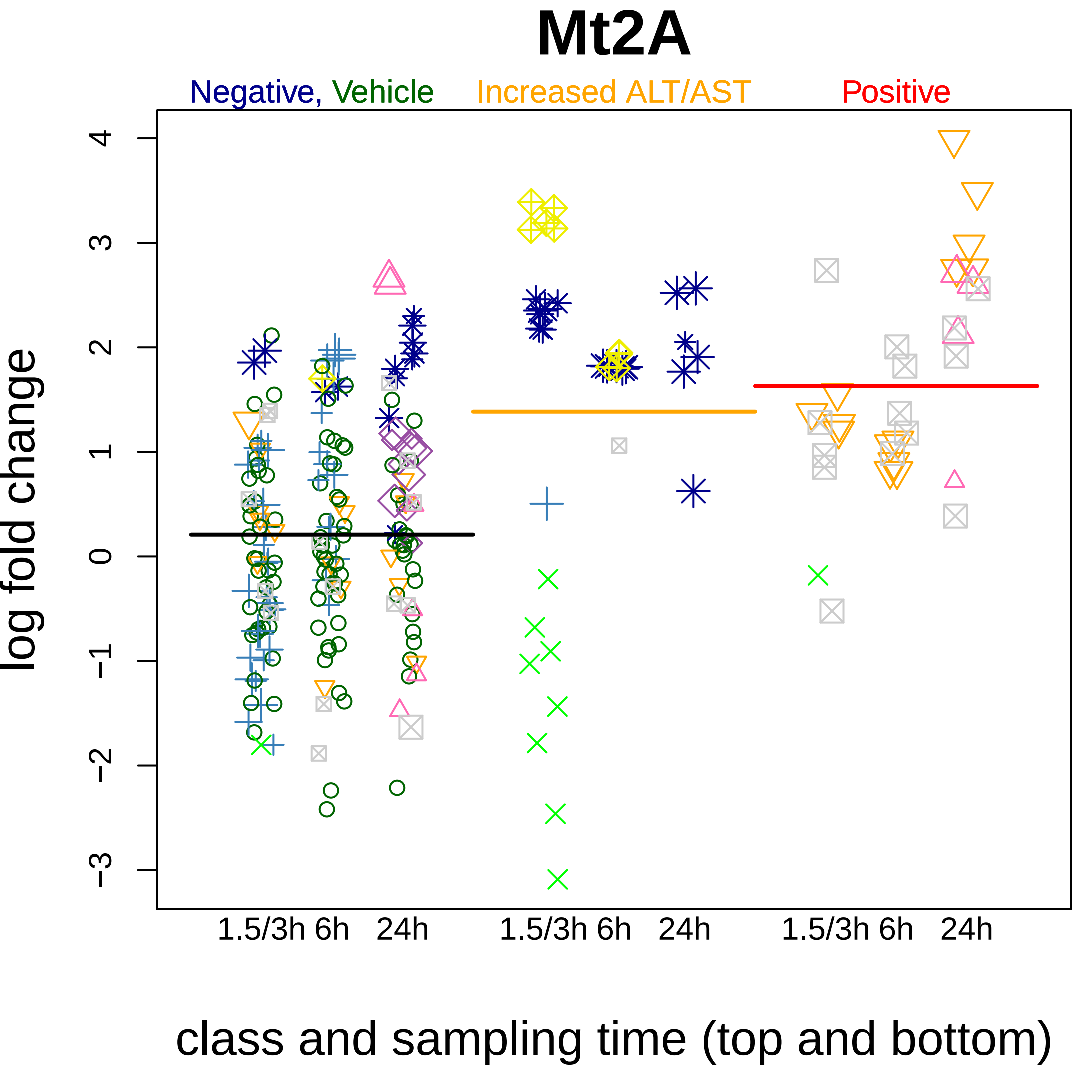

Mt2A
Negative, Vehicle
Increased ALT/AST
Positive
log fold change
4
3
2
1
0
−1
−2
−3
1.5/3h 6h 24h
1.5/3h 6h 24h
1.5/3h 6h 24h
class and sampling time (top and bottom)

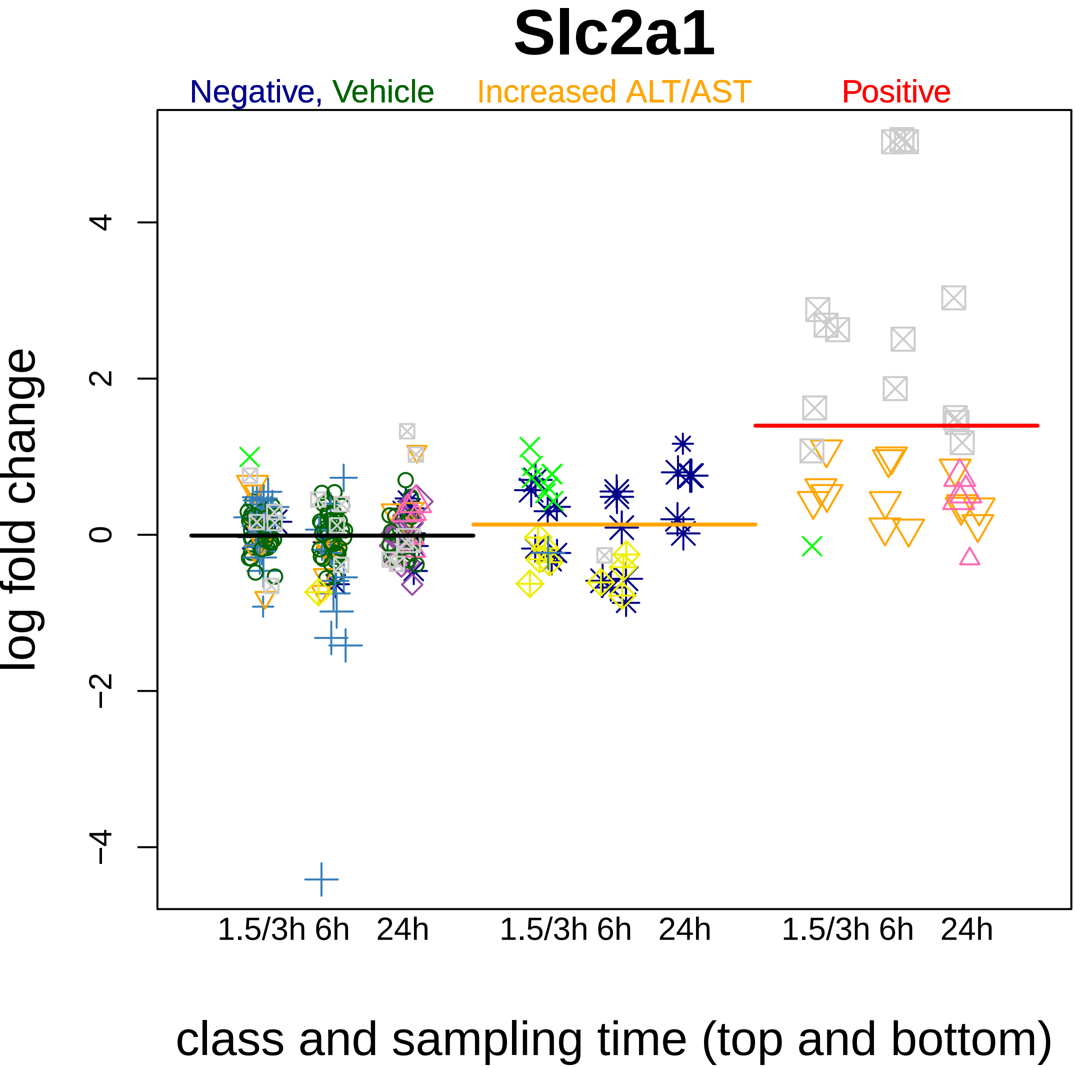

Slc2a1
Negative, Vehicle
Increased ALT/AST
Positive
log fold change
4
2
0
−2
−4
1.5/3h 6h 24h
1.5/3h 6h 24h
1.5/3h 6h 24h
class and sampling time (top and bottom)

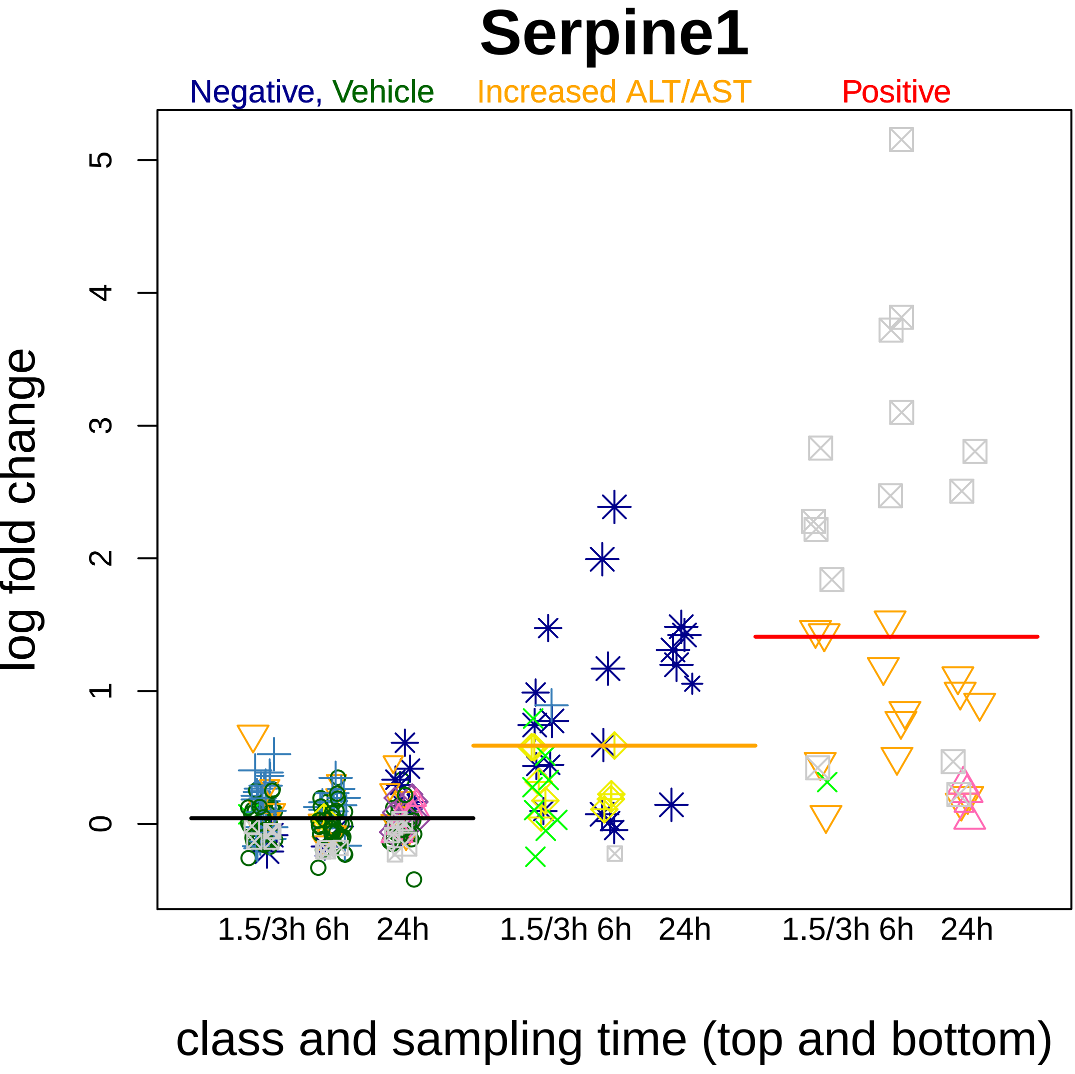
Serpine1
Negative, Vehicle
Increased ALT/AST
Positive
log fold change
0
1
2
3
4
5
1.5/3h 6h 24h
1.5/3h 6h 24h
1.5/3h 6h 24h
class and sampling time (top and bottom)

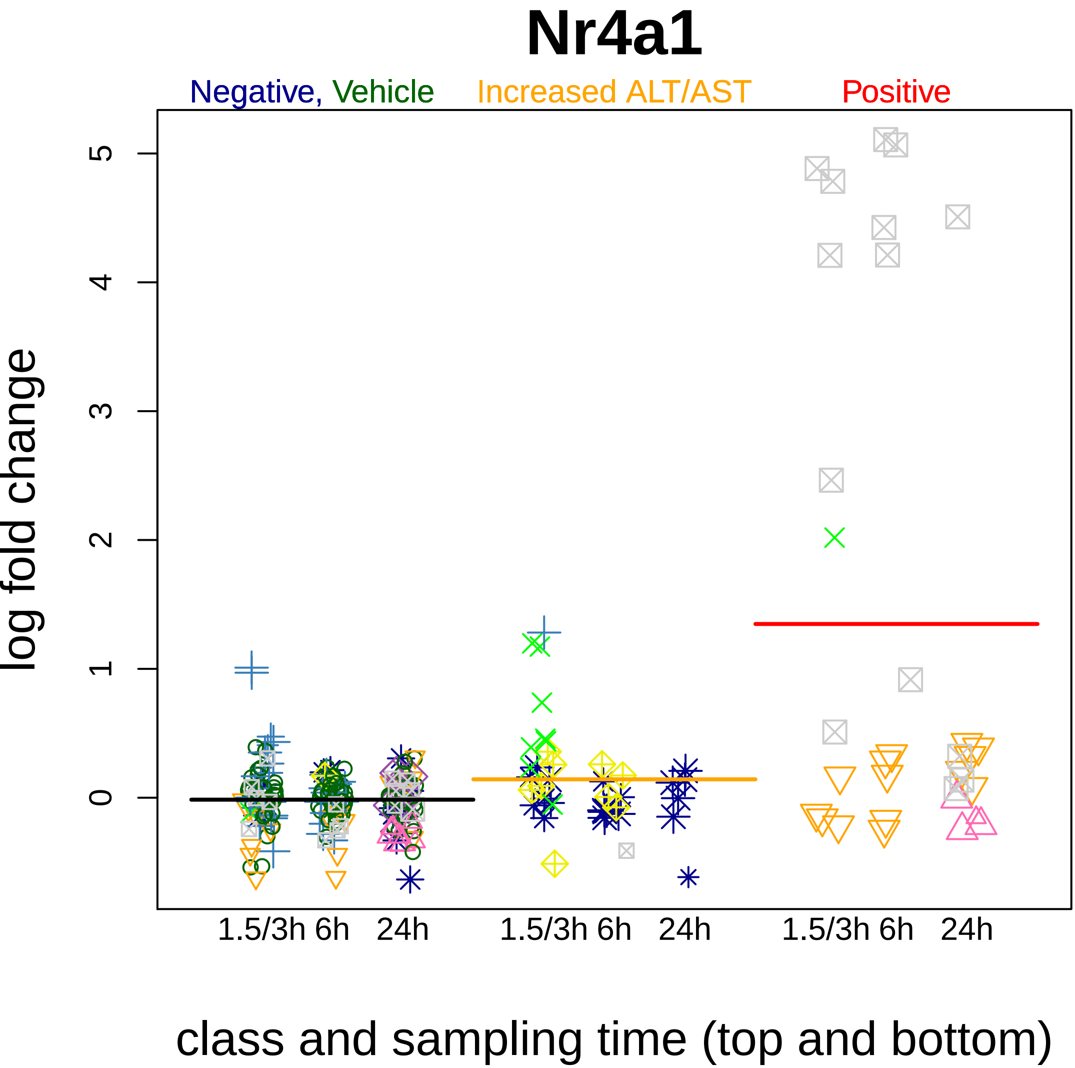

Nr4a1
Negative, Vehicle
Increased ALT/AST
Positive
log fold change
5
4
3
2
1
0
1.5/3h 6h 24h
1.5/3h 6h 24h
1.5/3h 6h 24h
class and sampling time (top and bottom)

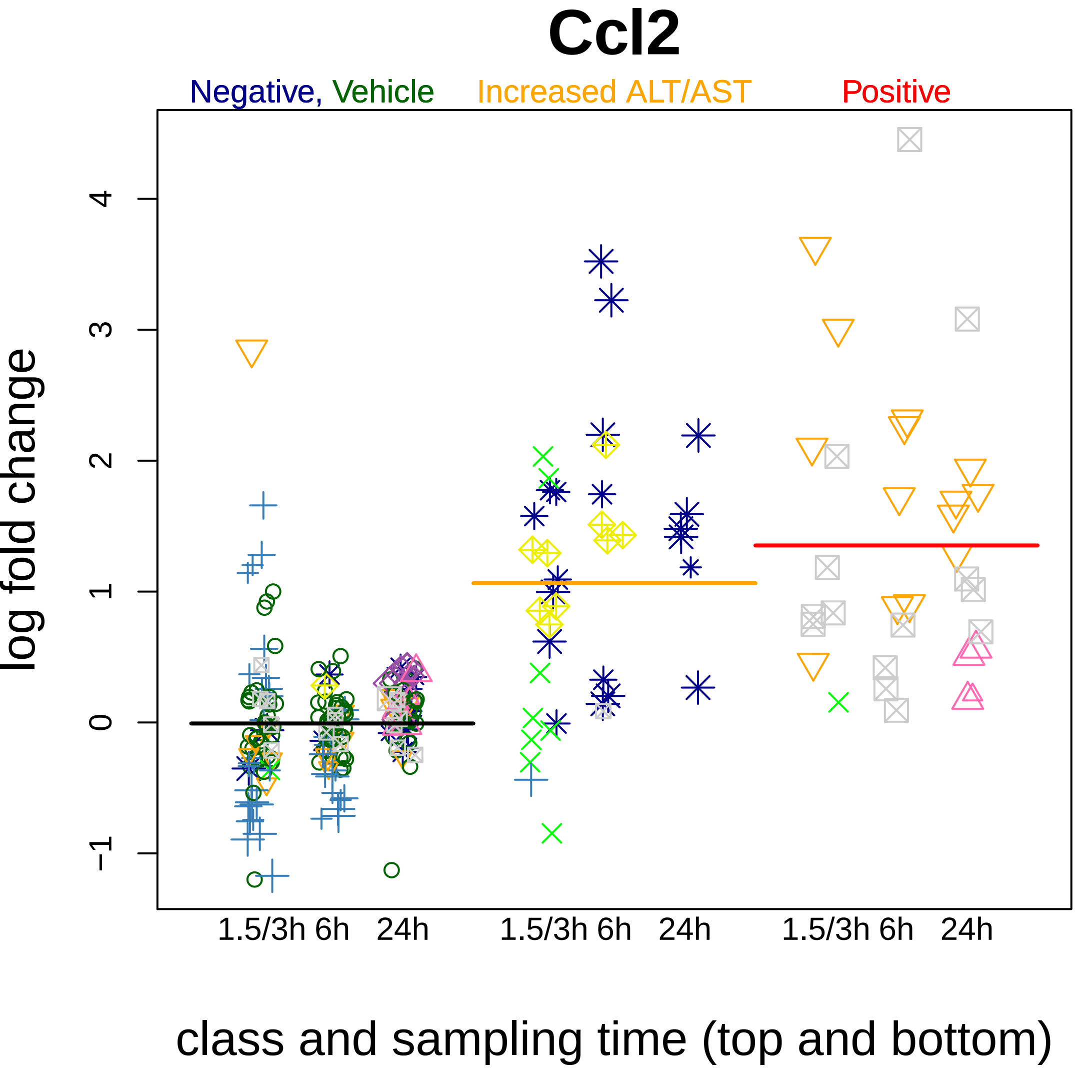

Ccl2
Negative, Vehicle
Increased ALT/AST
Positive
log fold change
4
3
2
1
0
−1
1.5/3h 6h 24h
1.5/3h 6h 24h
1.5/3h 6h 24h
class and sampling time (top and bottom)

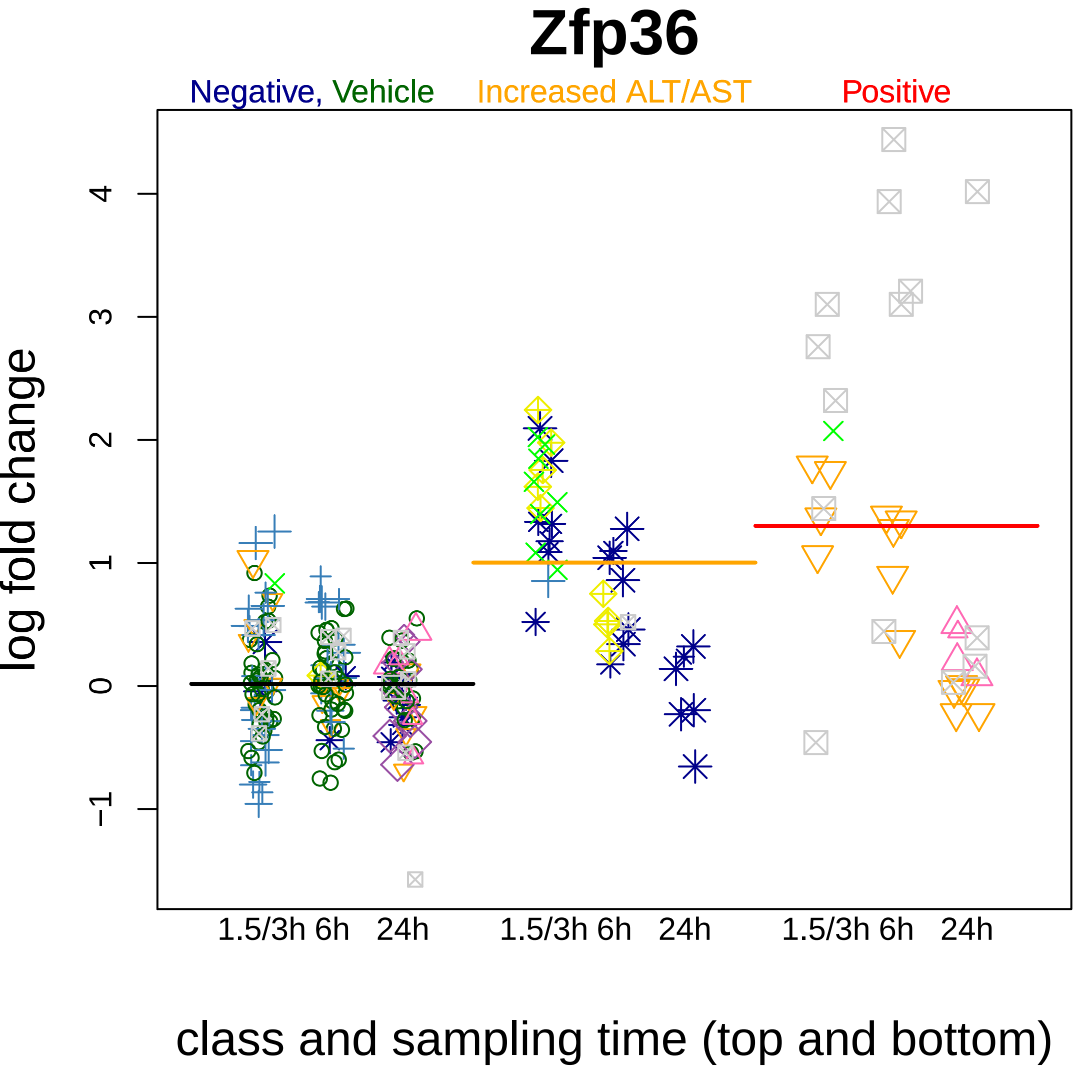
Zfp36
Negative, Vehicle
Increased ALT/AST
Positive
log fold change
−1
0
1
2
3
4
1.5/3h 6h
24h
1.5/3h 6h
24h
1.5/3h 6h
24h
class and sampling time (top and bottom)

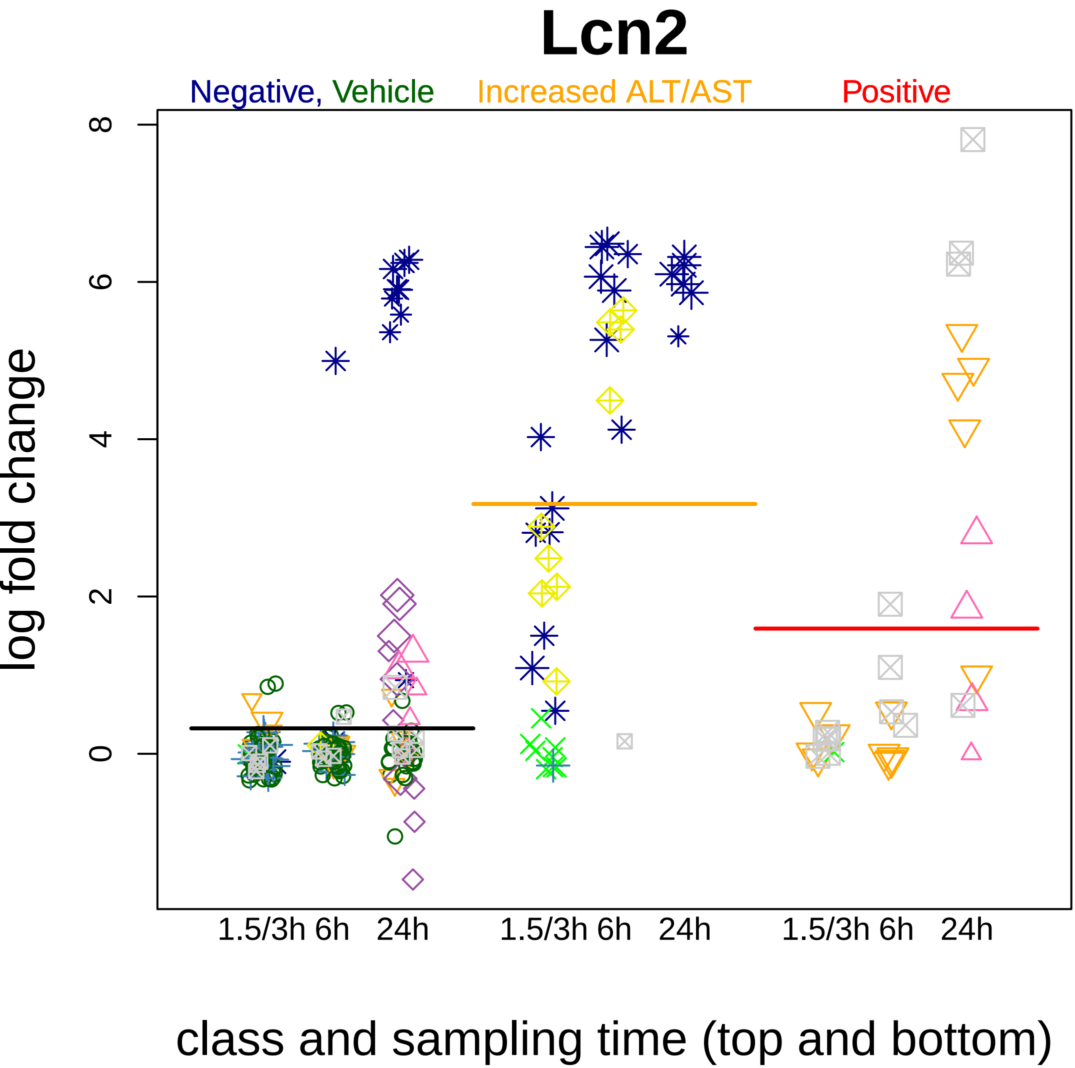
Lcn2
Negative, Vehicle
Increased ALT/AST
Positive
log fold change
0
2
4
6
8
1.5/3h 6h 24h
1.5/3h 6h 24h
1.5/3h 6h 24h
class and sampling time (top and bottom)

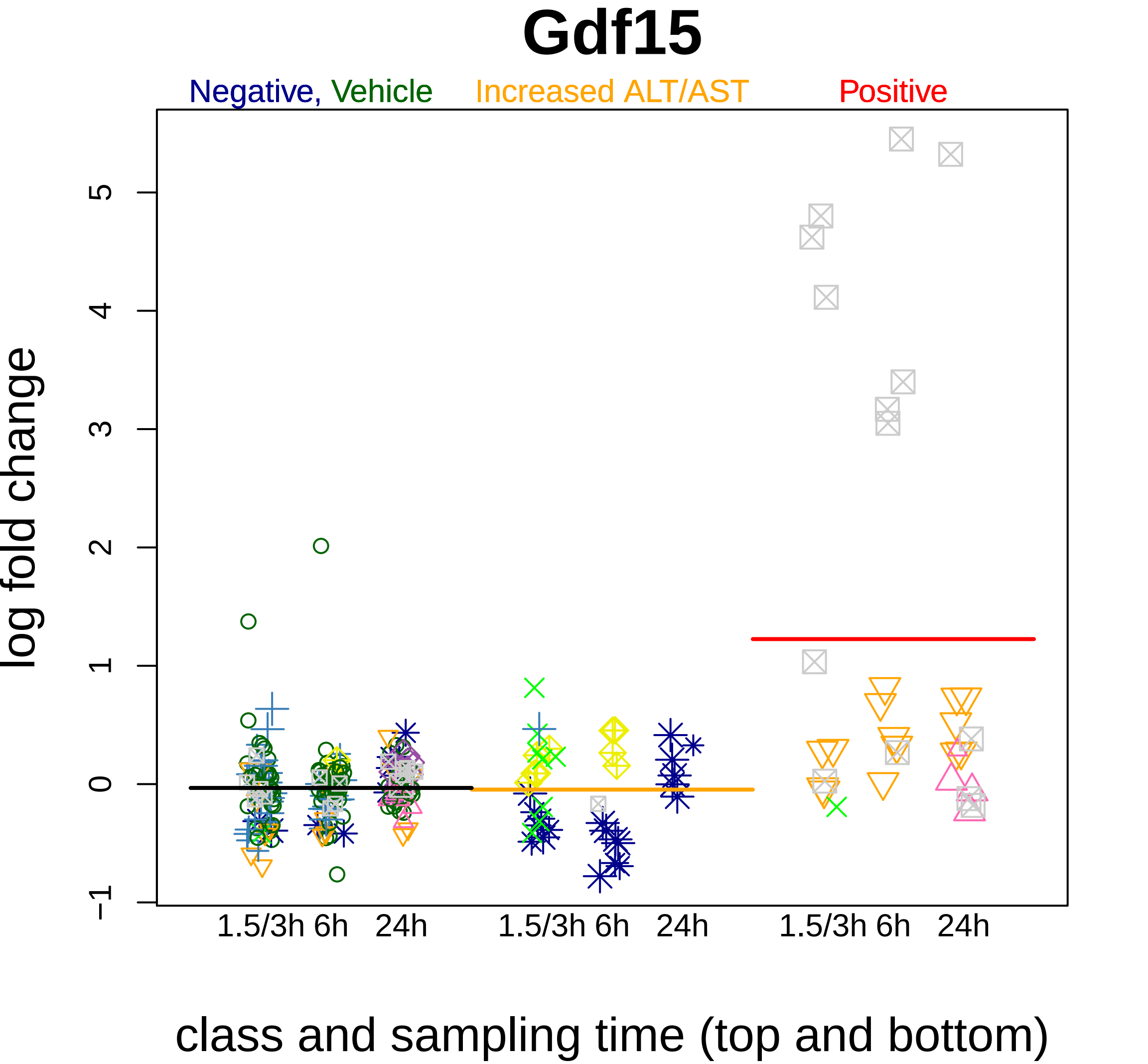

Gdf15
Negative, Vehicle
Increased ALT/AST
Positive
log fold change
−1
0
1
2
3
4
5
1.5/3h 6h 24h
1.5/3h 6h 24h
1.5/3h 6h 24h
class and sampling time (top and bottom)

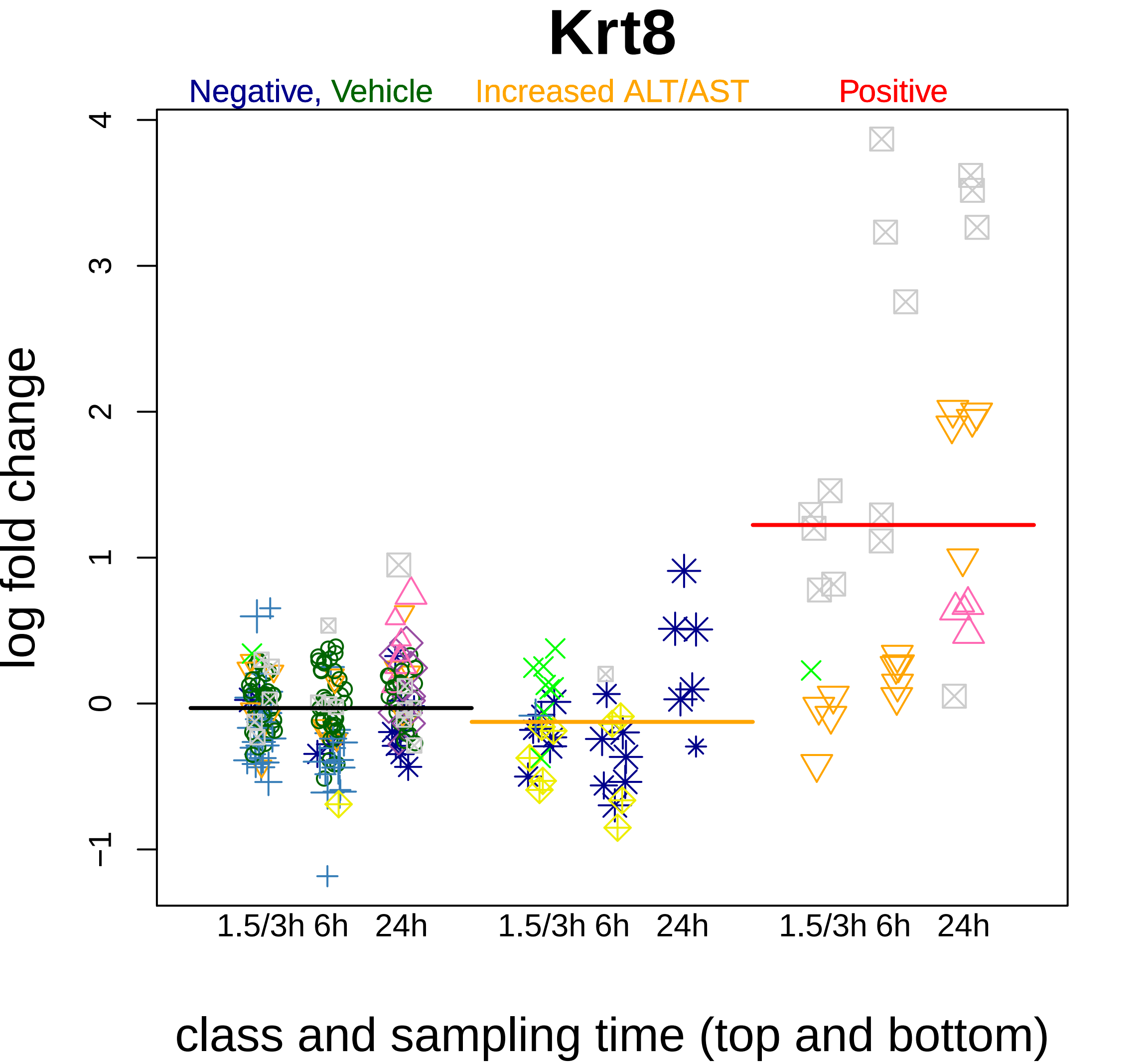

Krt8
Negative, Vehicle
Increased ALT/AST
Positive
log fold change
4
3
2
1
0
−1
1.5/3h 6h 24h
1.5/3h 6h 24h
1.5/3h 6h 24h
class and sampling time (top and bottom)

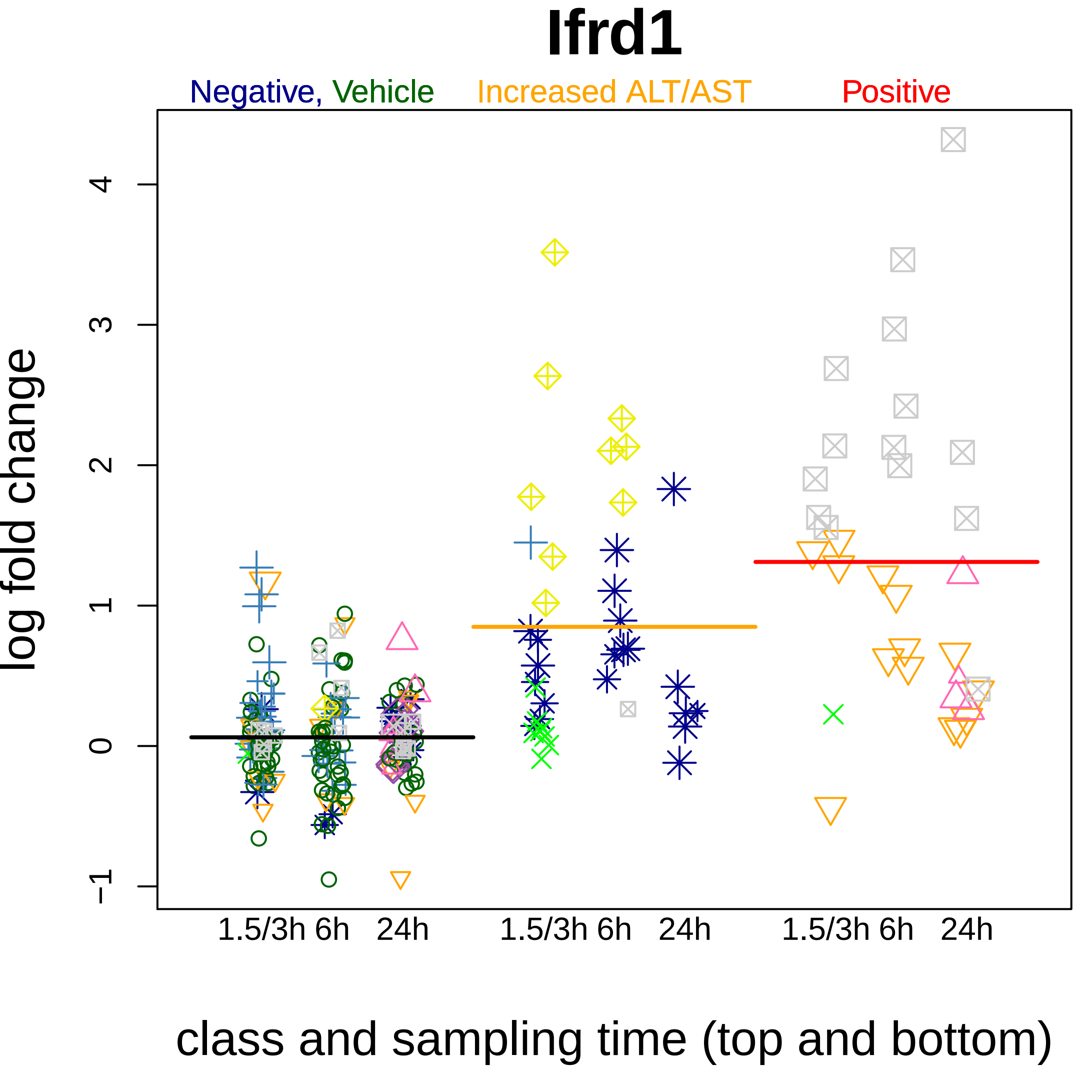

Ifrd1
Negative, Vehicle
Increased ALT/AST
Positive
log fold change
4
3
2
1
0
−1
1.5/3h 6h 24h
1.5/3h 6h 24h
1.5/3h 6h 24h
class and sampling time (top and bottom)

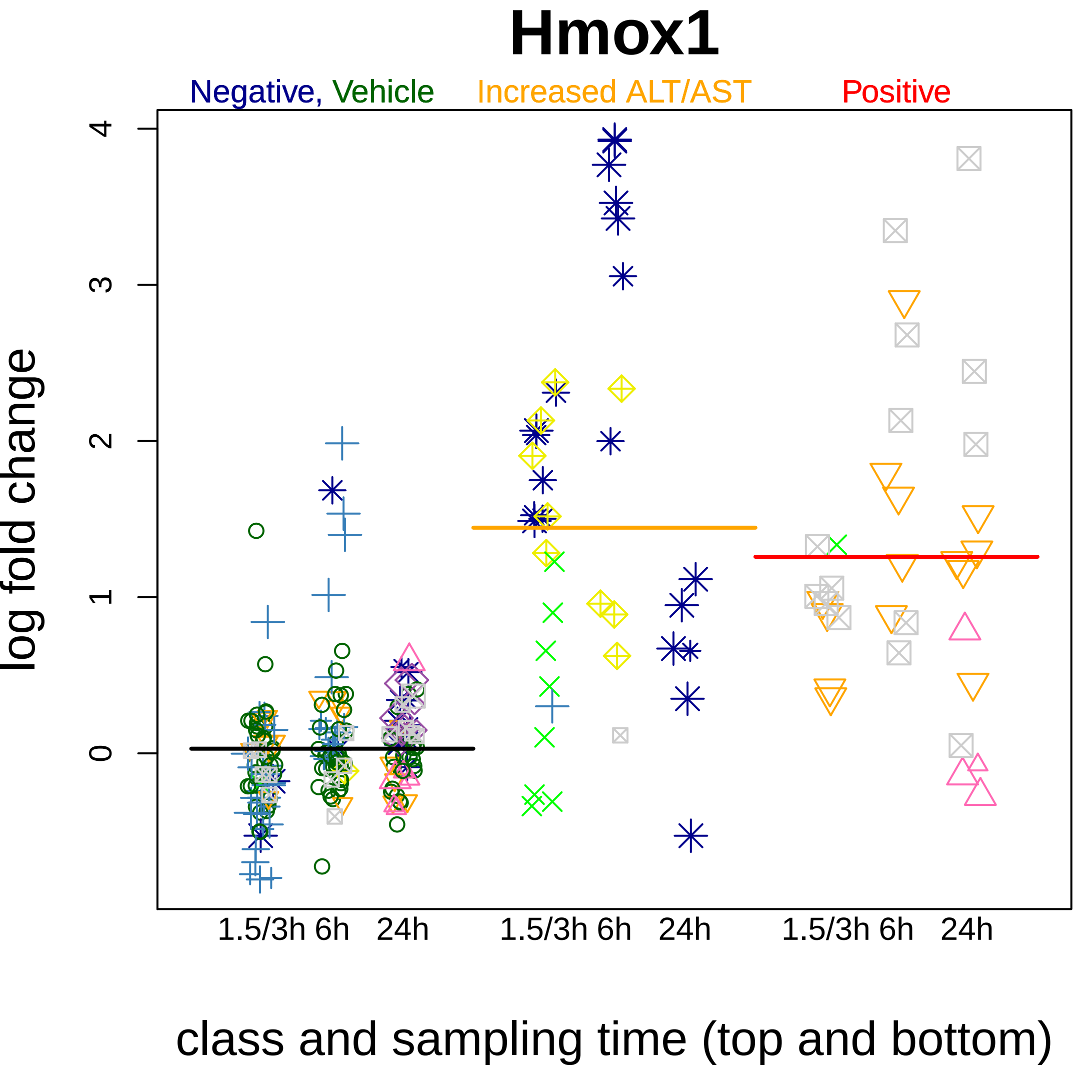

Hmox1
Negative, Vehicle
Increased ALT/AST
Positive
log fold change
0
1
2
3
4
1.5/3h 6h 24h
1.5/3h 6h 24h
1.5/3h 6h 24h
class and sampling time (top and bottom)

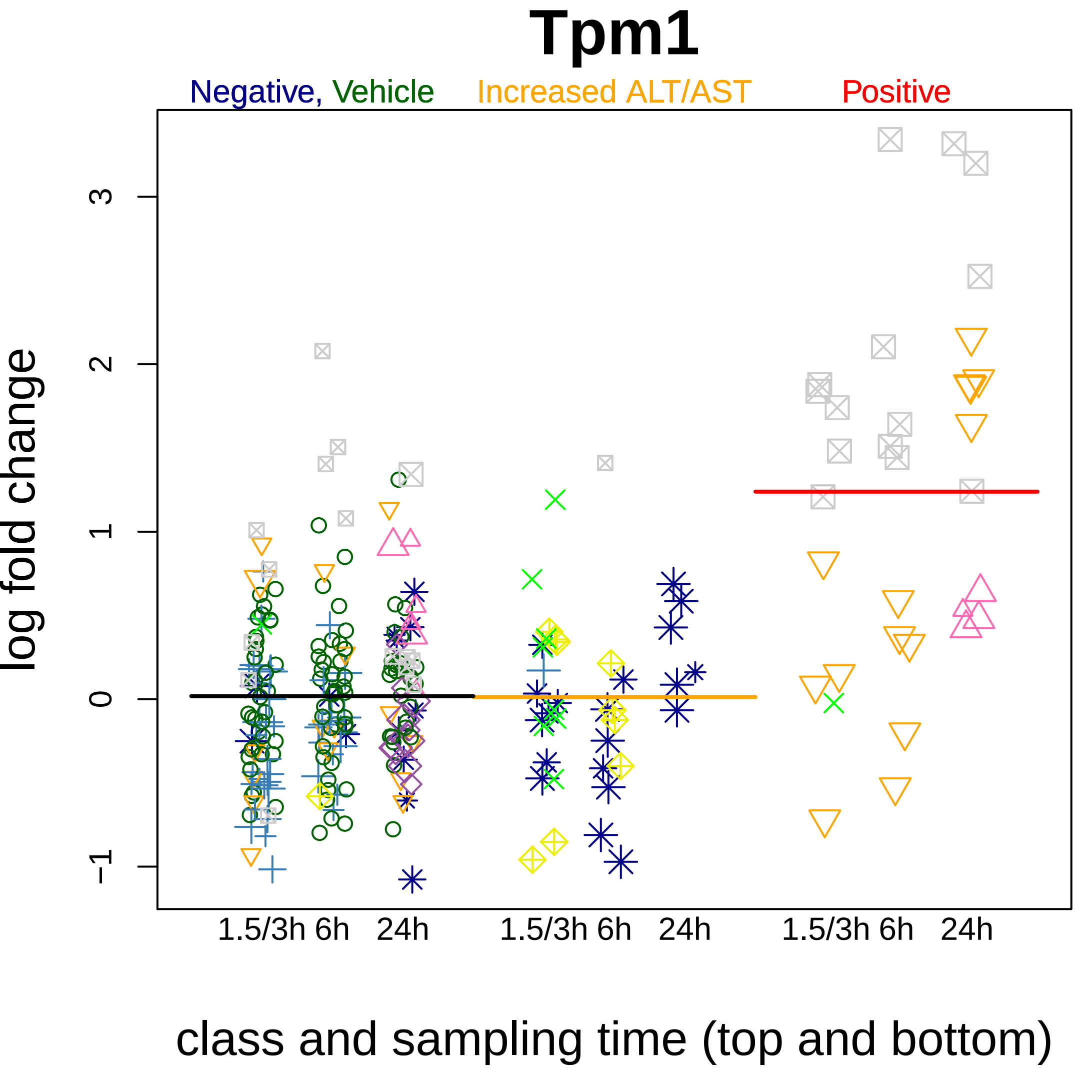

Tpm1
Negative, Vehicle
Increased ALT/AST
Positive
log fold change
−1
0
1
2
3
1.5/3h 6h 24h
1.5/3h 6h 24h
1.5/3h 6h 24h
class and sampling time (top and bottom)

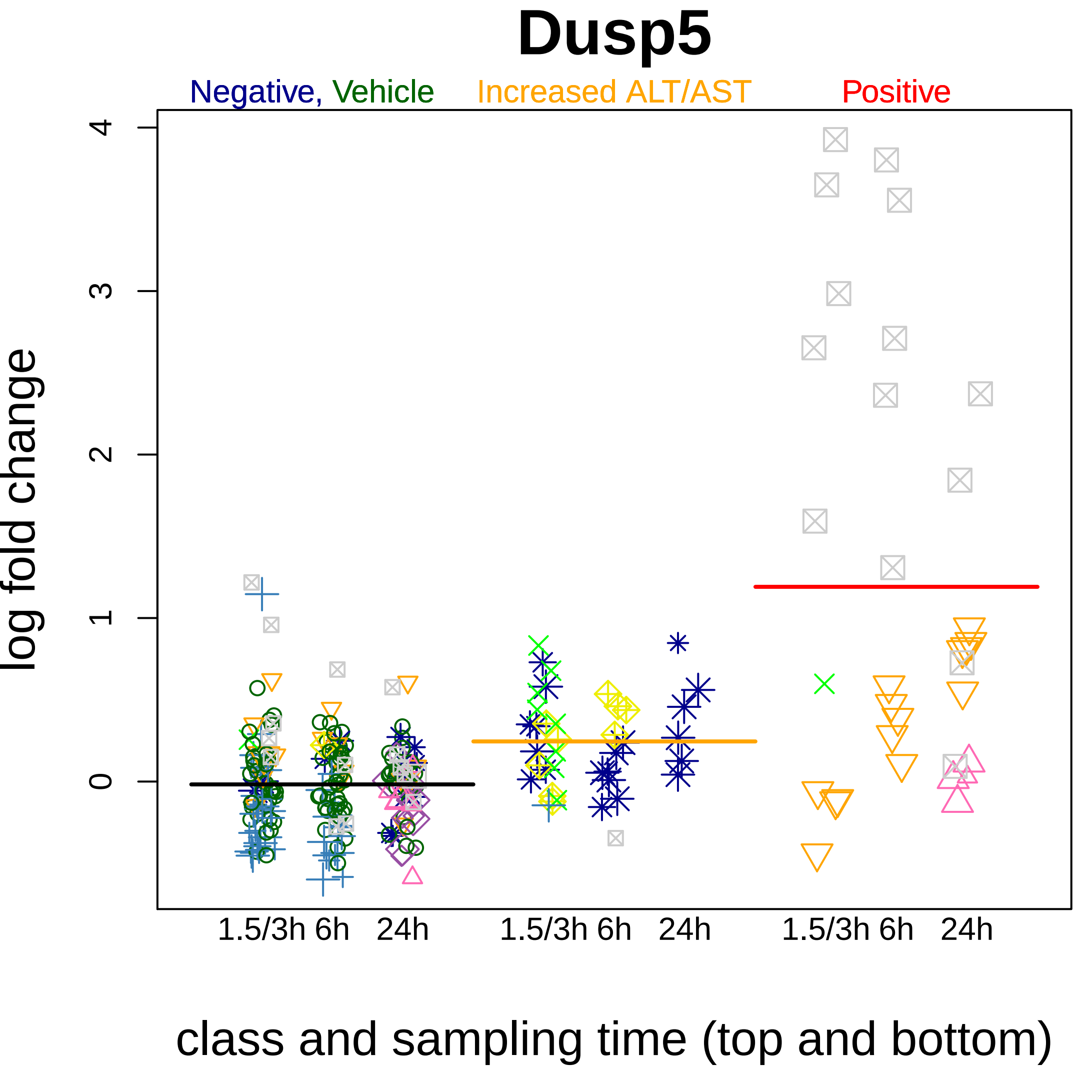

Dusp5
Negative, Vehicle
Increased ALT/AST
Positive
log fold change
0
1
2
3
4
1.5/3h 6h 24h
1.5/3h 6h 24h
1.5/3h 6h 24h
class and sampling time (top and bottom)

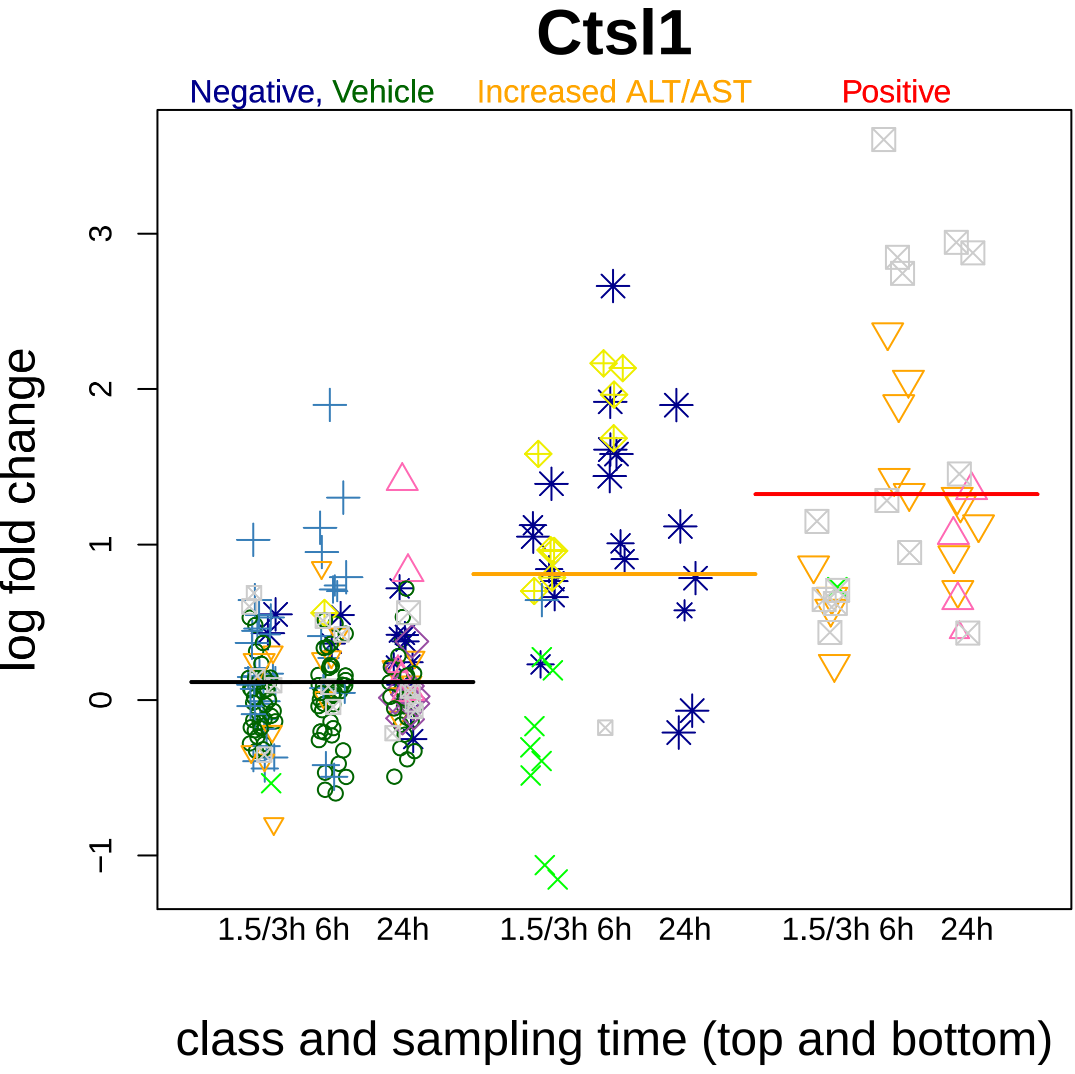

Ctsl1
Negative, Vehicle
Increased ALT/AST
Positive
log fold change
−1
0
1
2
3
1.5/3h 6h 24h
1.5/3h 6h 24h
1.5/3h 6h 24h
class and sampling time (top and bottom)

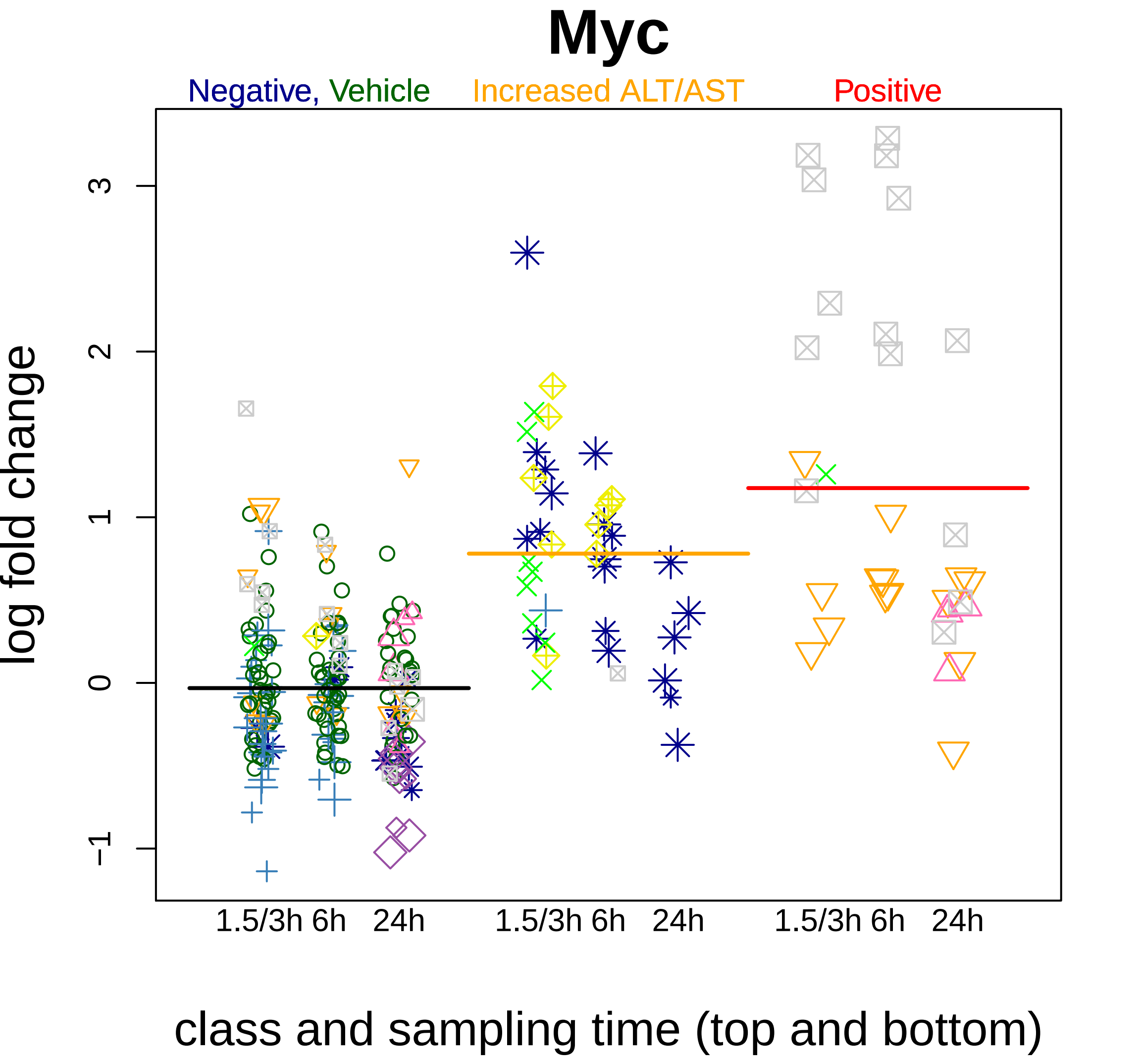
Myc
Negative, Vehicle
Increased ALT/AST
Positive
log fold change
3
2
1
0
−1
1.5/3h 6h 24h
1.5/3h 6h 24h
1.5/3h 6h 24h
class and sampling time (top and bottom)

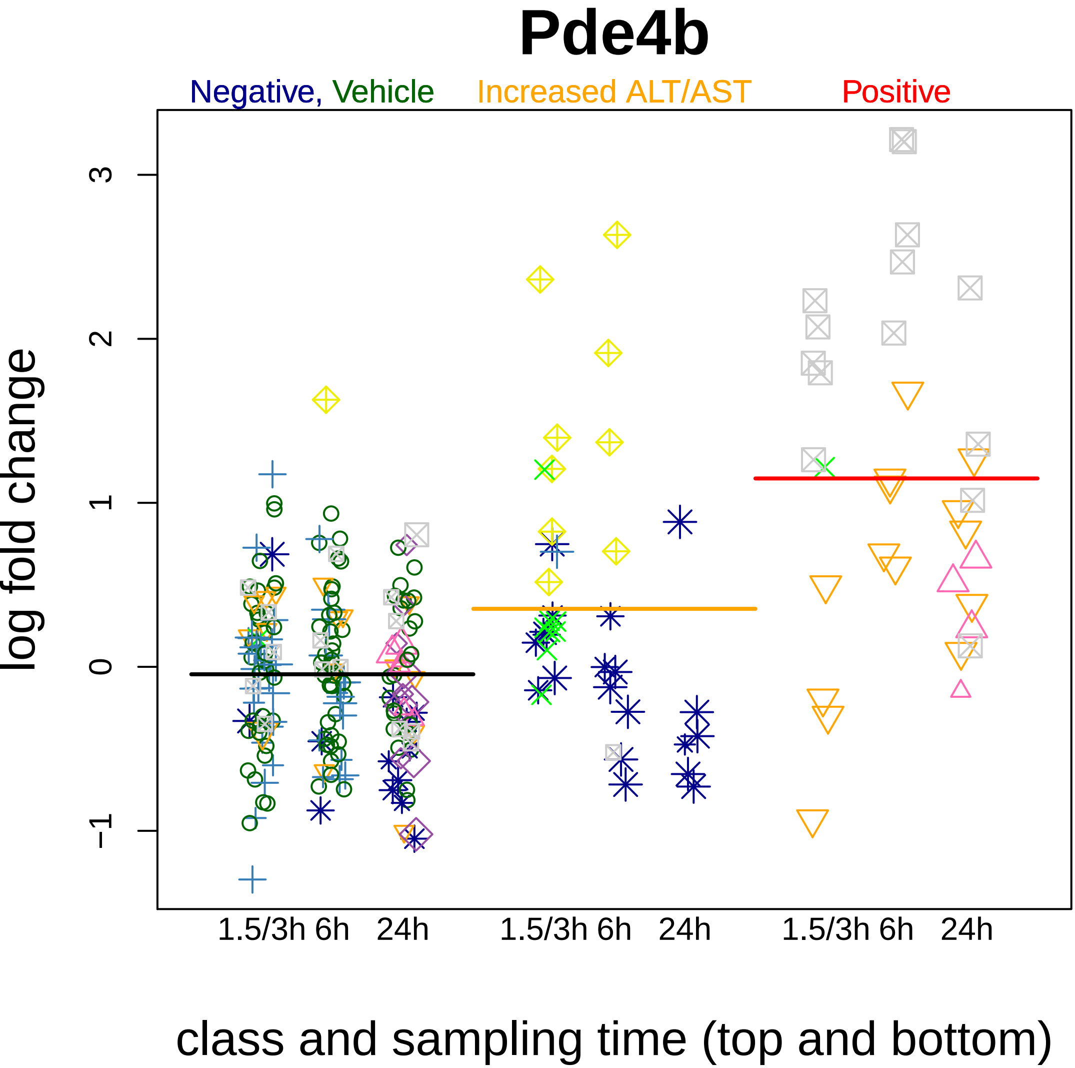
Pde4b
Negative, Vehicle
Increased ALT/AST
Positive
log fold change
3
2
1
0
−1
1.5/3h 6h 24h
1.5/3h 6h 24h
1.5/3h 6h 24h
class and sampling time (top and bottom)

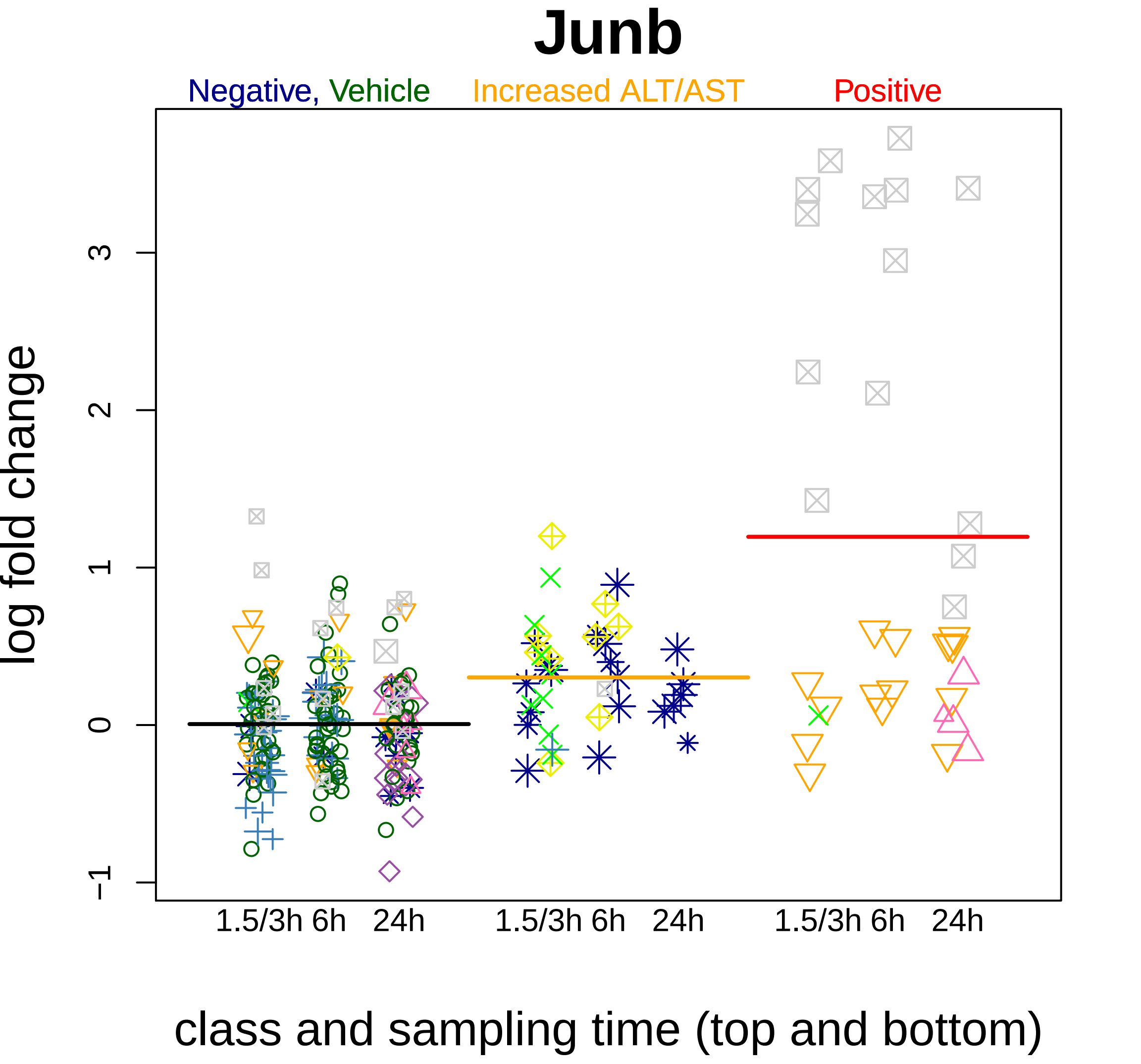

Junb
Negative, Vehicle
Increased ALT/AST
Positive
log fold change
3
2
1
0
−1
1.5/3h 6h 24h
1.5/3h 6h 24h
1.5/3h 6h 24h
class and sampling time (top and bottom)

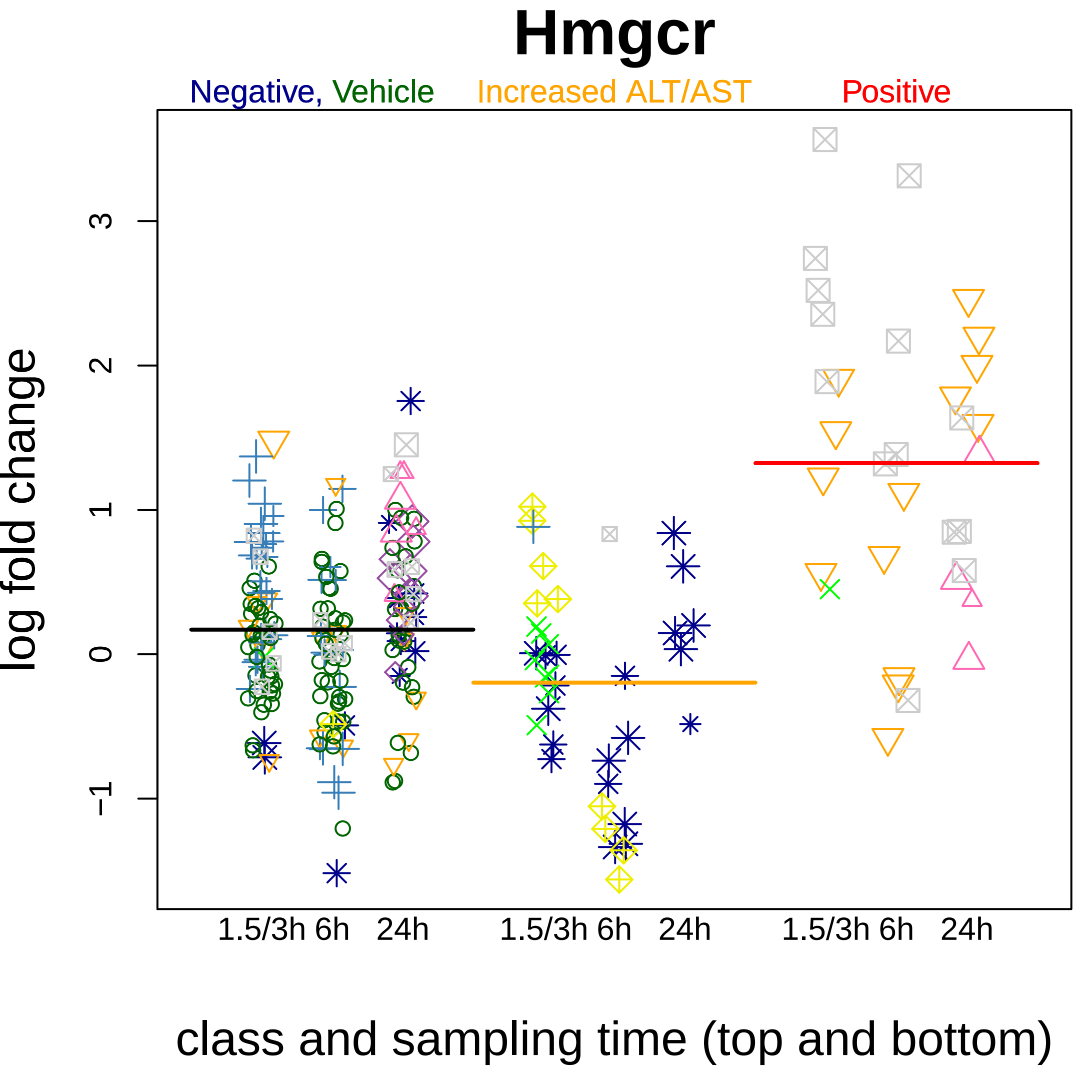
Hmgcr
Negative, Vehicle
Increased ALT/AST
Positive
log fold change
3
2
1
0
−1
1.5/3h 6h 24h
1.5/3h 6h 24h
1.5/3h 6h 24h
class and sampling time (top and bottom)

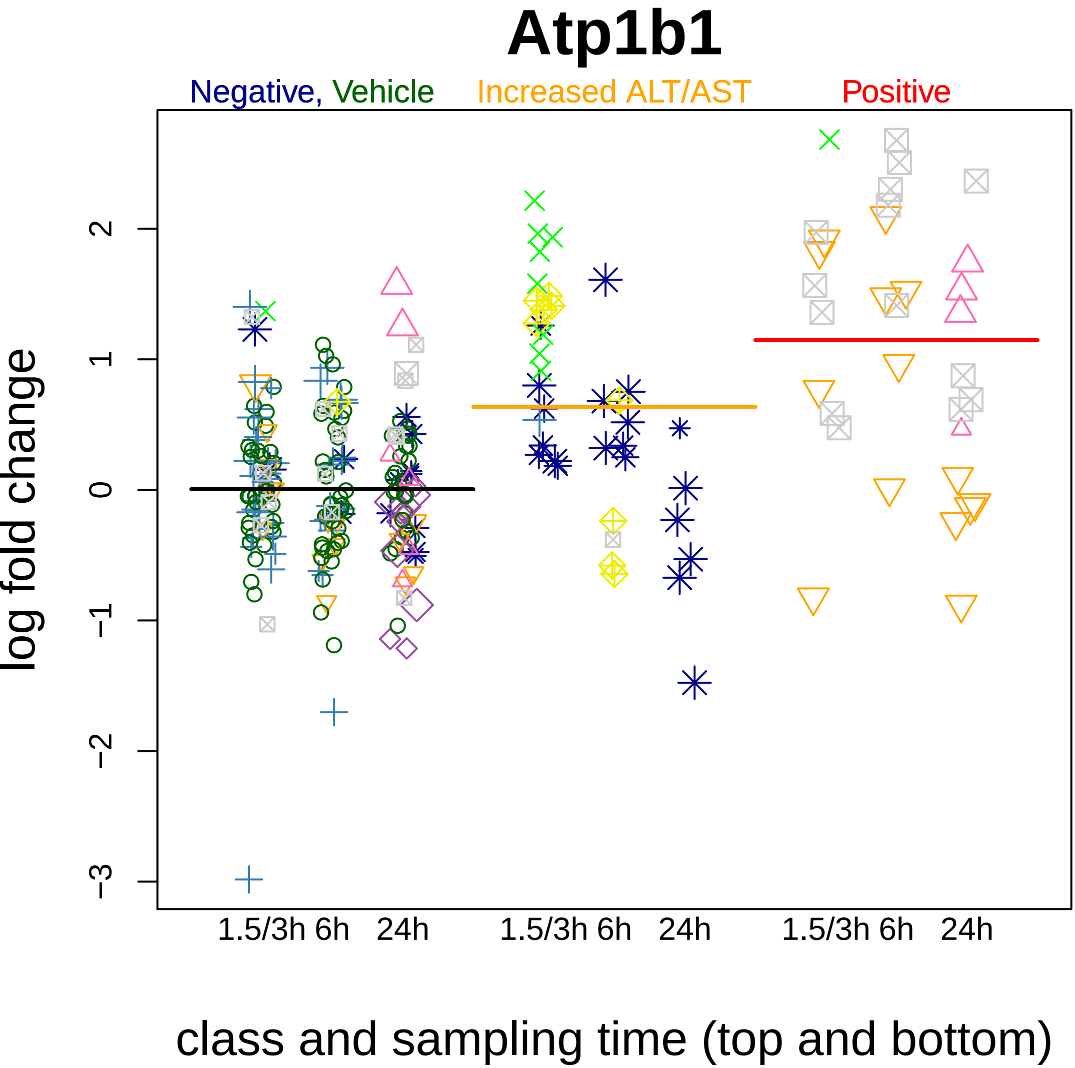

Atp1b1
Negative, Vehicle
Increased ALT/AST
Positive
log fold change
2
1
0
−1
−2
−3
1.5/3h 6h 24h
1.5/3h 6h 24h
1.5/3h 6h 24h
class and sampling time (top and bottom)

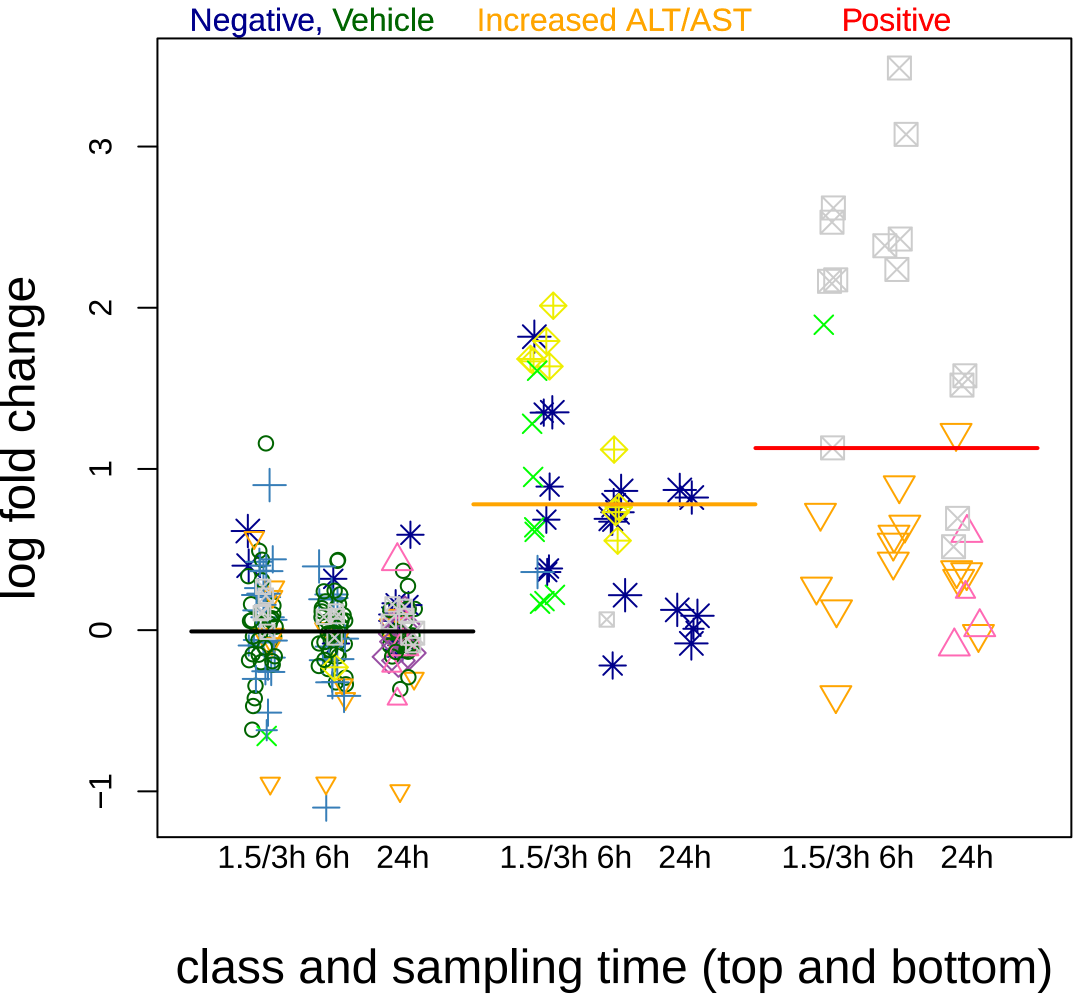

Negative, Vehicle
Increased ALT/AST
Positive
log fold change
-1
0
1
2
3
1.5/3h 6h 24h
1.5/3h 6h 24h
1.5/3h 6h 24h
class and sampling time (top and bottom)

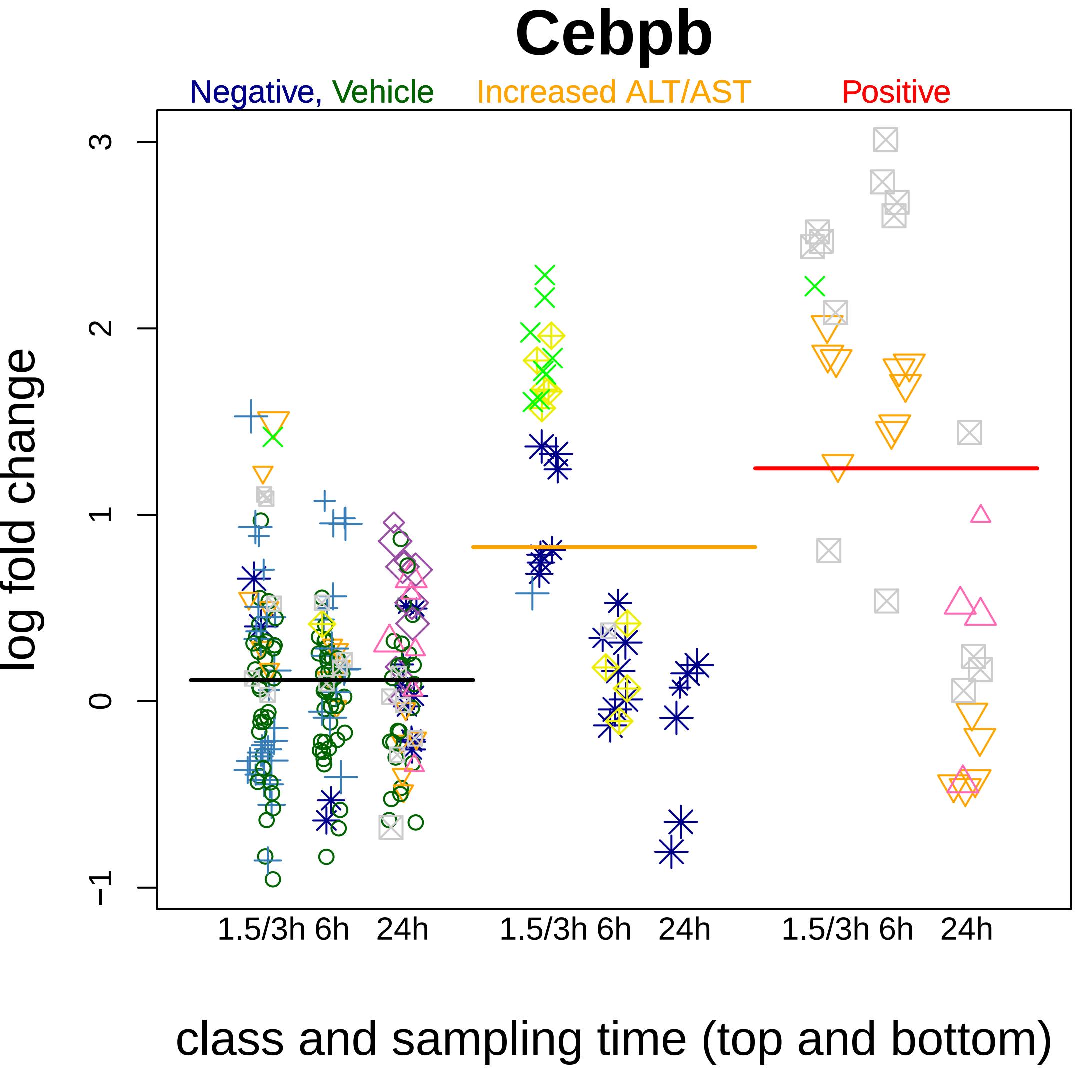
Cebpb
Negative, Vehicle
Increased ALT/AST
Positive
log fold change
3
2
1
0
−1
1.5/3h 6h 24h
1.5/3h 6h 24h
1.5/3h 6h 24h
class and sampling time (top and bottom)

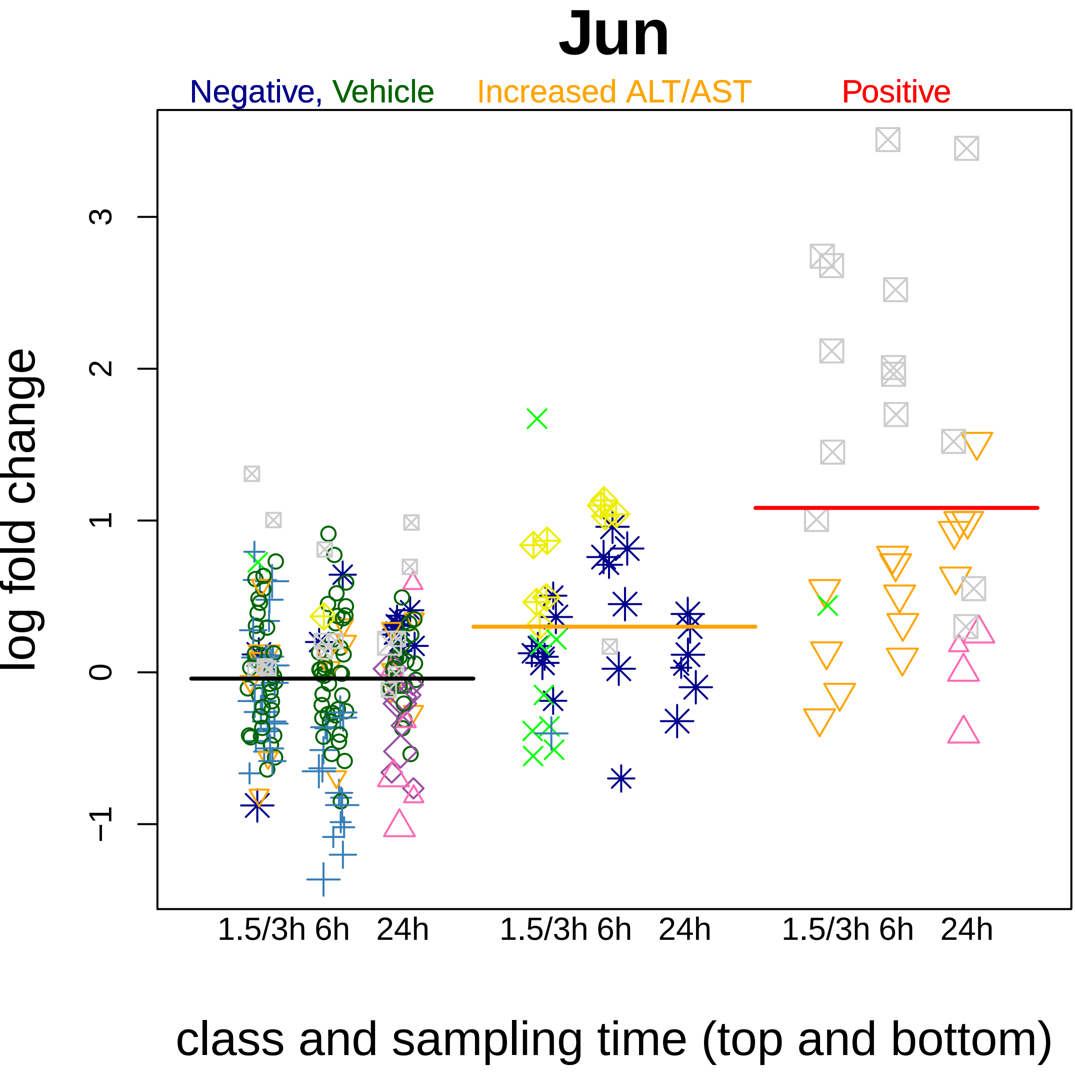
Jun
Negative, Vehicle
Increased ALT/AST
Positive
log fold change
−1
0
1
2
3
1.5/3h 6h 24h
1.5/3h 6h 24h
1.5/3h 6h 24h
class and sampling time (top and bottom)

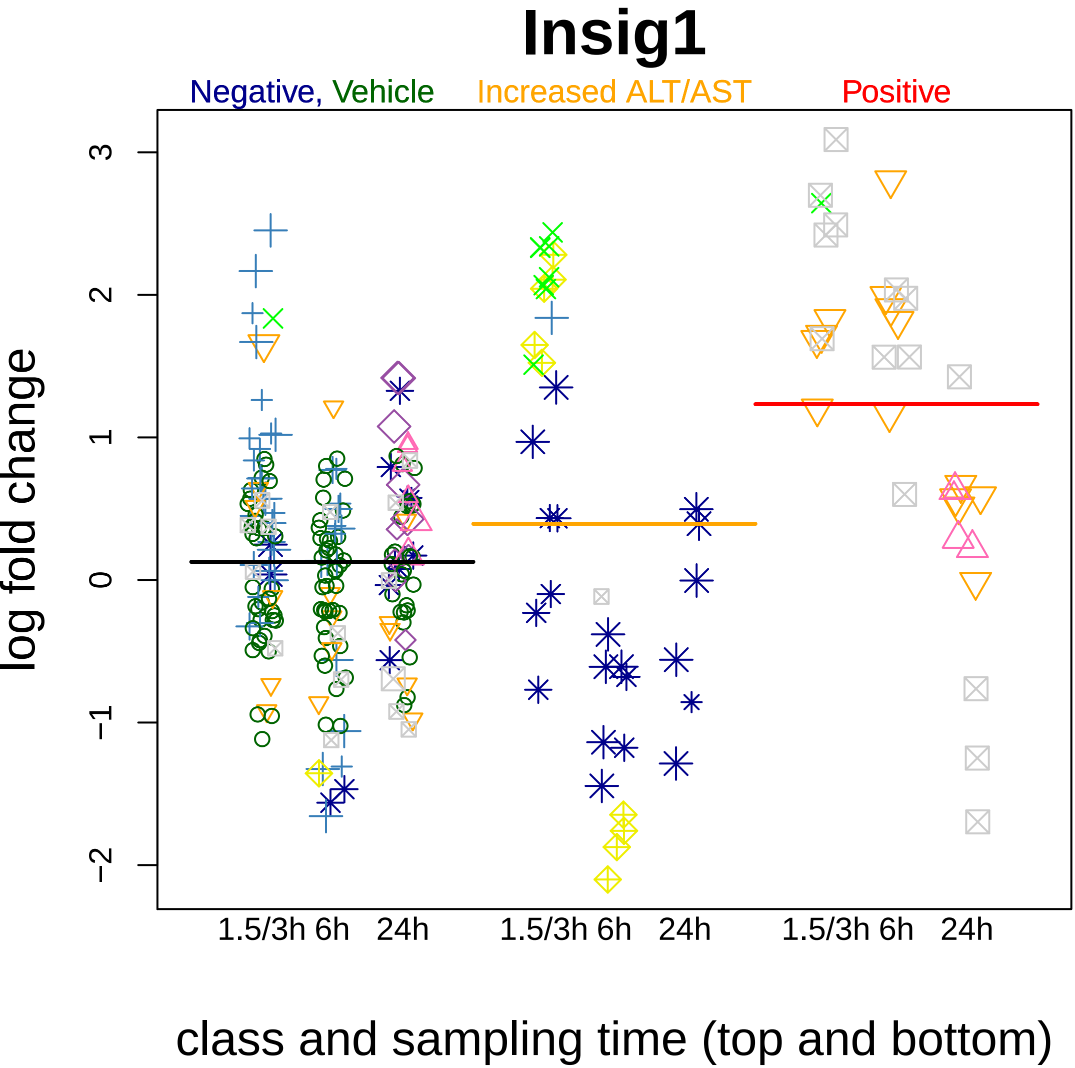
Insig1
Negative, Vehicle
Increased ALT/AST
Positive
log fold change
3
2
1
0
−1
−2
1.5/3h 6h 24h
1.5/3h 6h 24h
1.5/3h 6h 24h
class and sampling time (top and bottom)

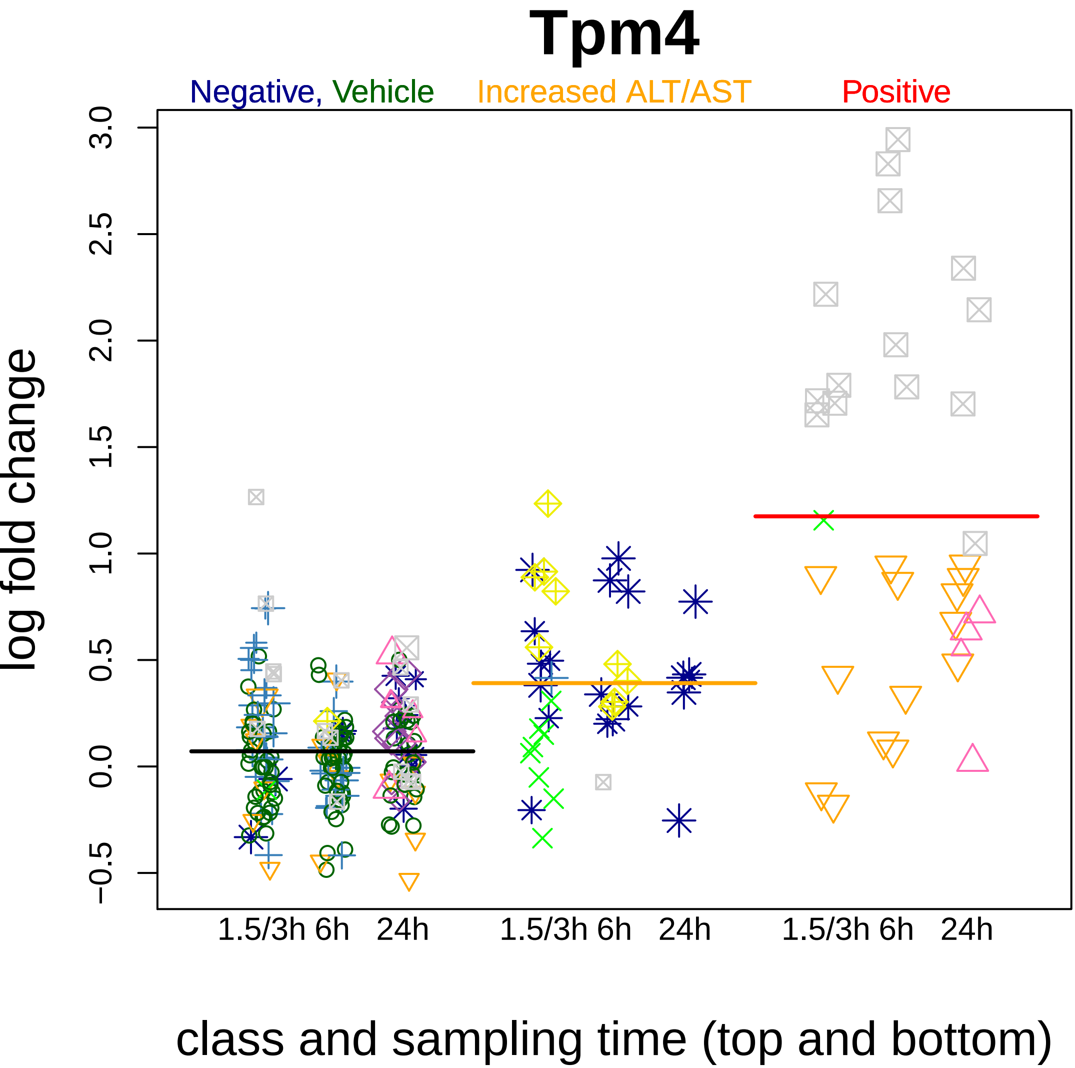

Tpm4
Negative, Vehicle
Increased ALT/AST
Positive
log fold change
−0.5
0.0
0.5
1.0
1.5
2.0
2.5
3.0
1.5/3h 6h 24h
1.5/3h 6h 24h
1.5/3h 6h 24h
class and sampling time (top and bottom)

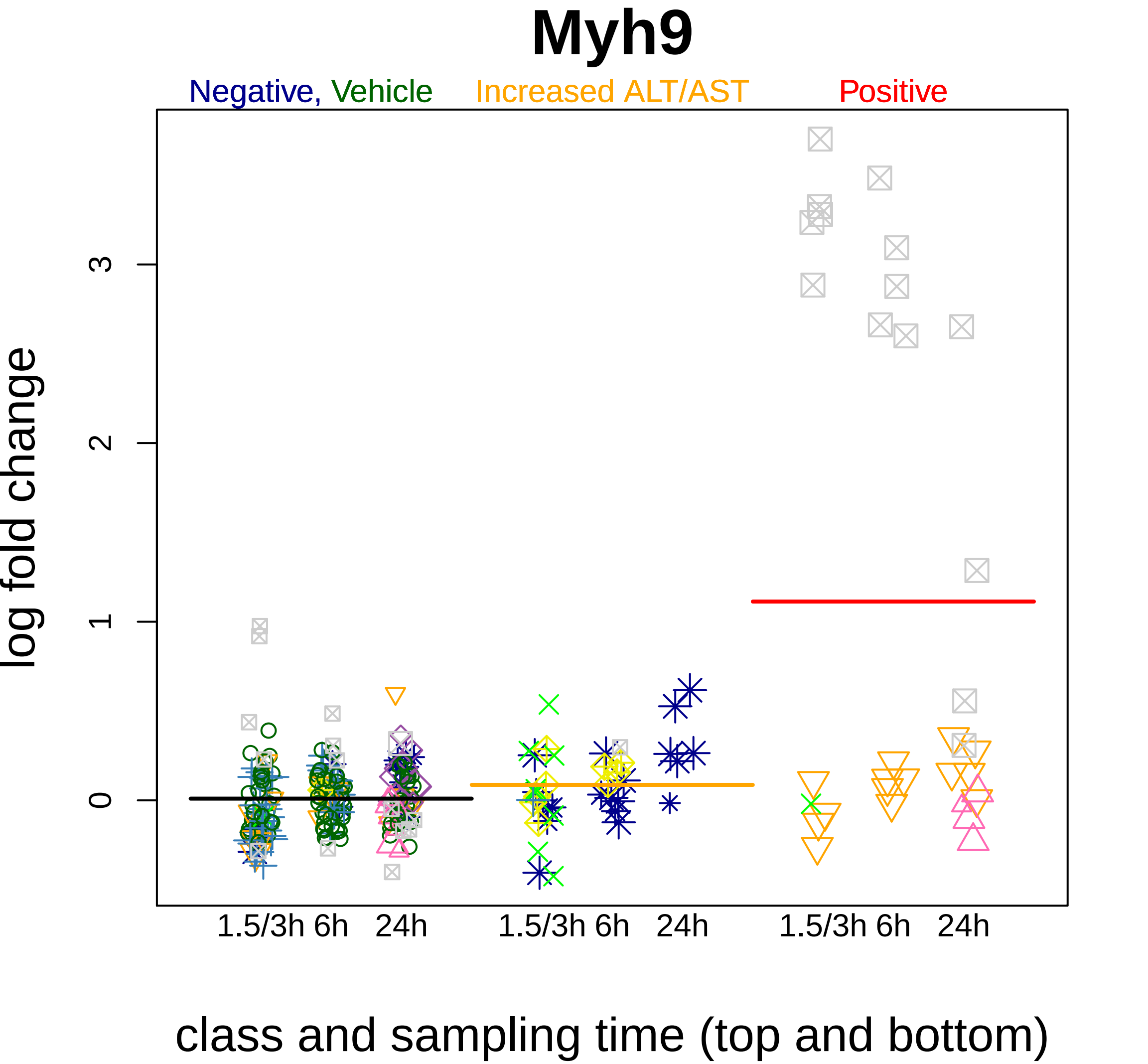

Myh9
Negative, Vehicle
Increased ALT/AST
Positive
log fold change
0
1
2
3
1.5/3h 6h 24h
1.5/3h 6h 24h
1.5/3h 6h 24h
class and sampling time (top and bottom)

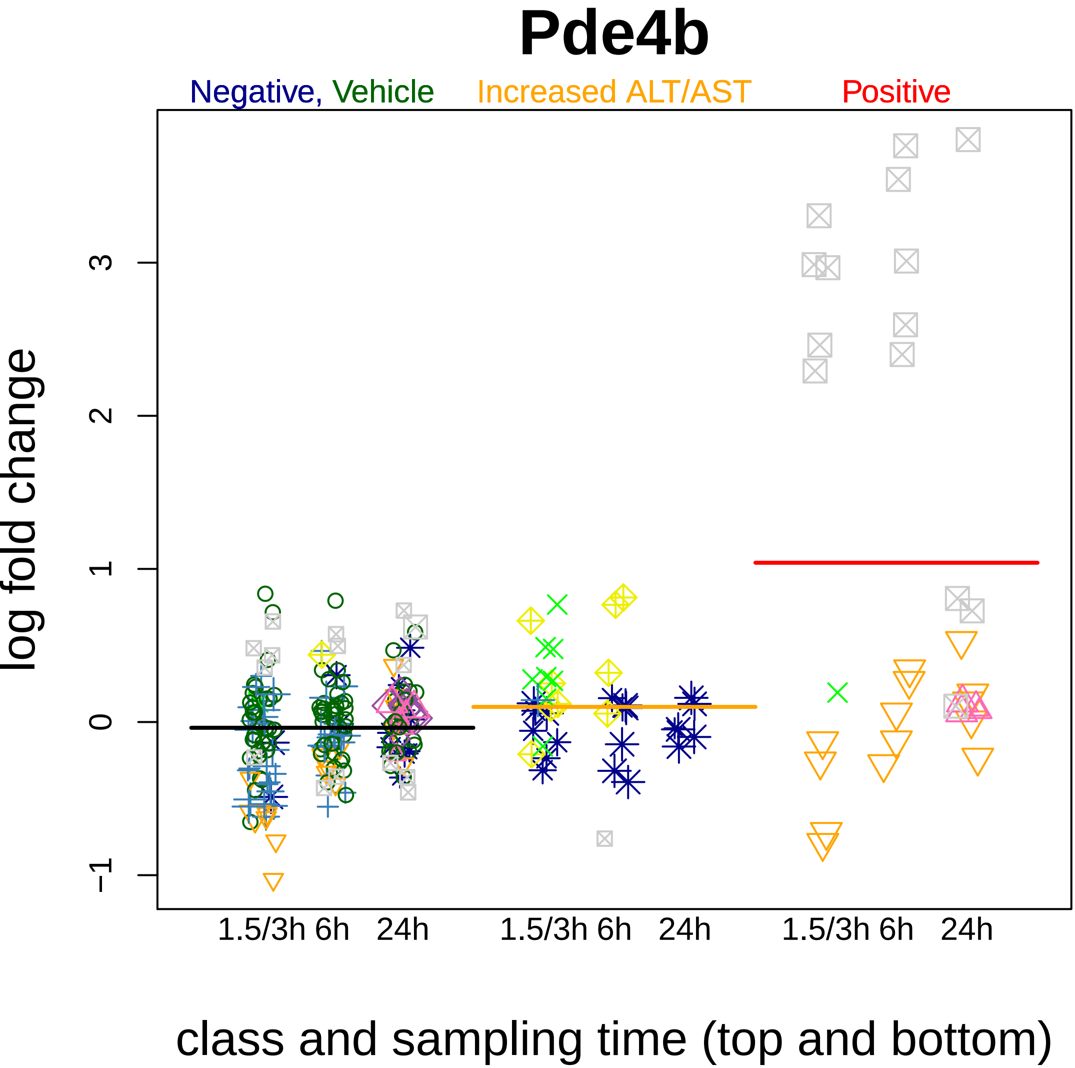
Pde4b
Negative, Vehicle
Increased ALT/AST
Positive
log fold change
−1
0
1
2
3
1.5/3h 6h 24h
1.5/3h 6h 24h
1.5/3h 6h 24h
class and sampling time (top and bottom)

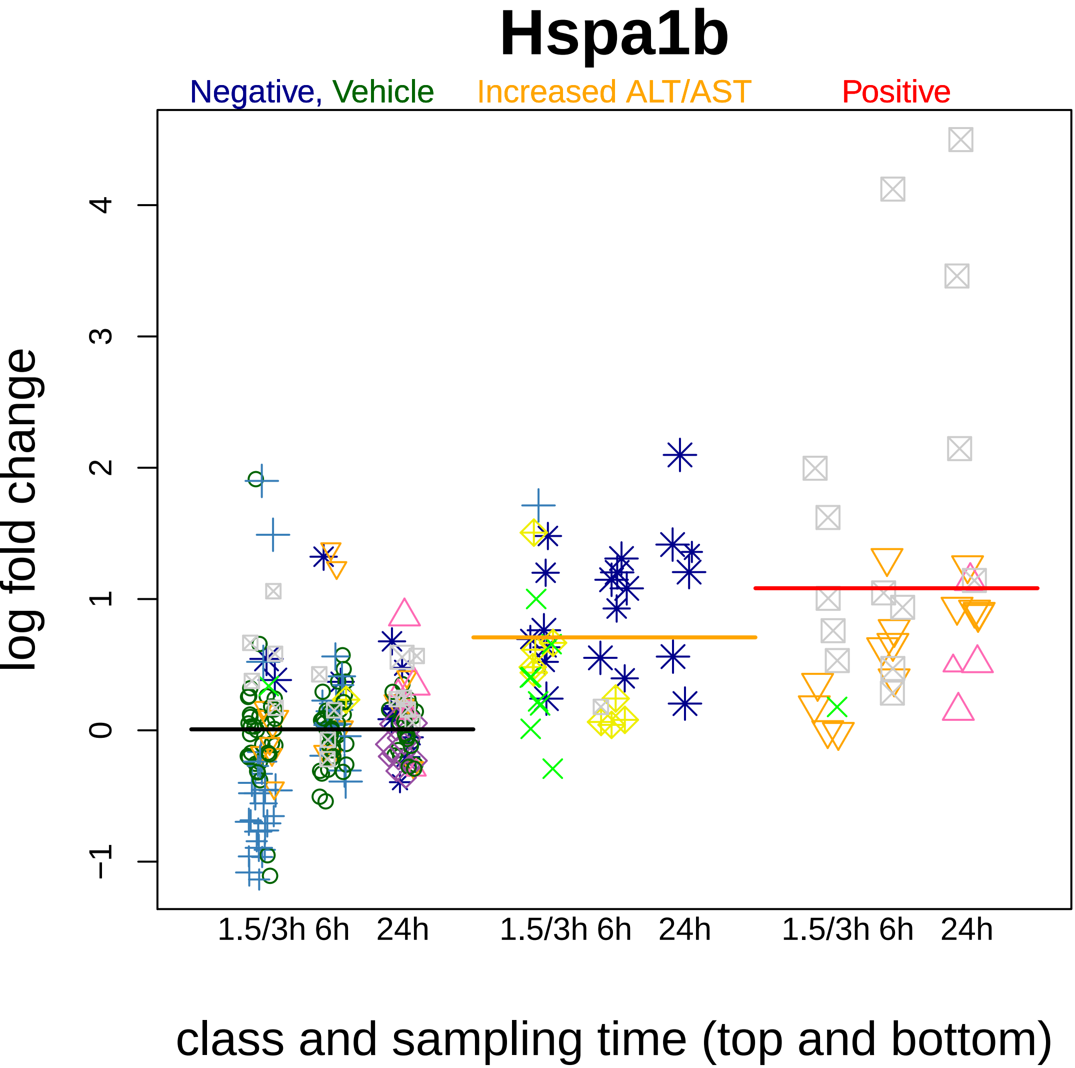

Hspa1b
Negative, Vehicle
Increased ALT/AST
Positive
log fold change
−1
0
1
2
3
4
1.5/3h 6h 24h
1.5/3h 6h 24h
1.5/3h 6h 24h
class and sampling time (top and bottom)

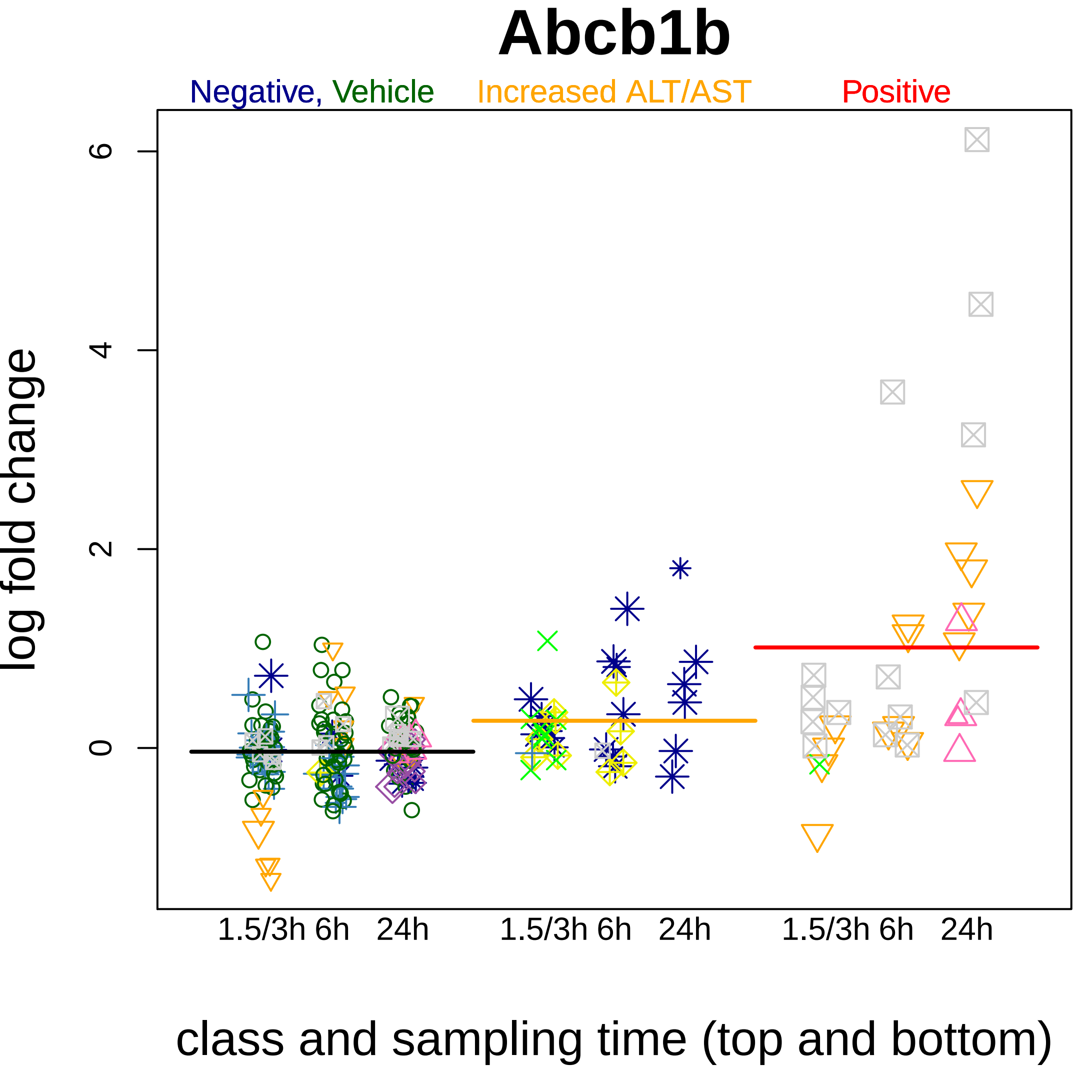
Abcb1b
Negative, Vehicle
Increased ALT/AST
Positive
log fold change
0
2
4
6
1.5/3h 6h 24h
1.5/3h 6h 24h
1.5/3h 6h 24h
class and sampling time (top and bottom)

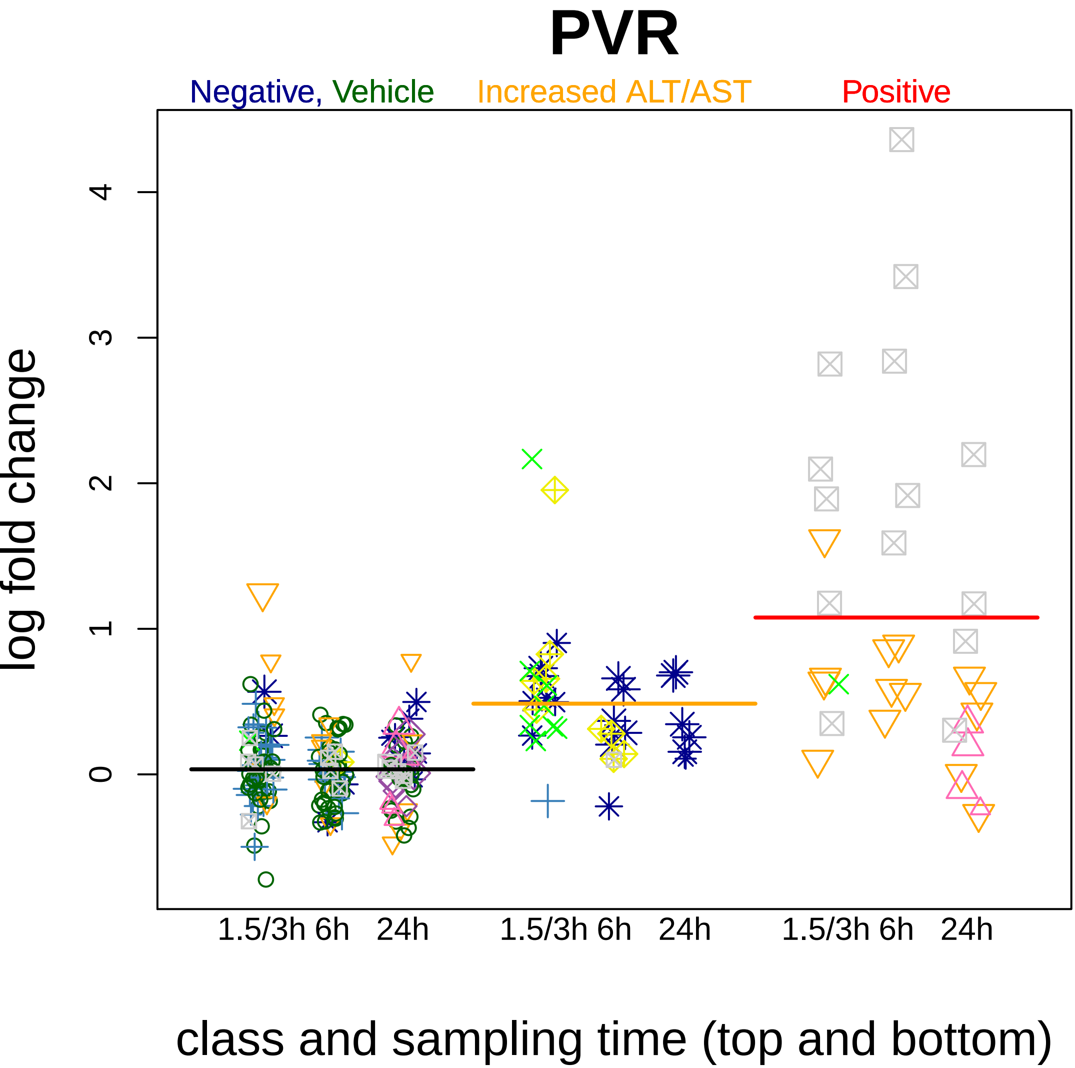
PVR
Negative, Vehicle
Increased ALT/AST
Positive
log fold change
0
1
2
3
4
1.5/3h 6h 24h
1.5/3h 6h 24h
1.5/3h 6h 24h
class and sampling time (top and bottom)

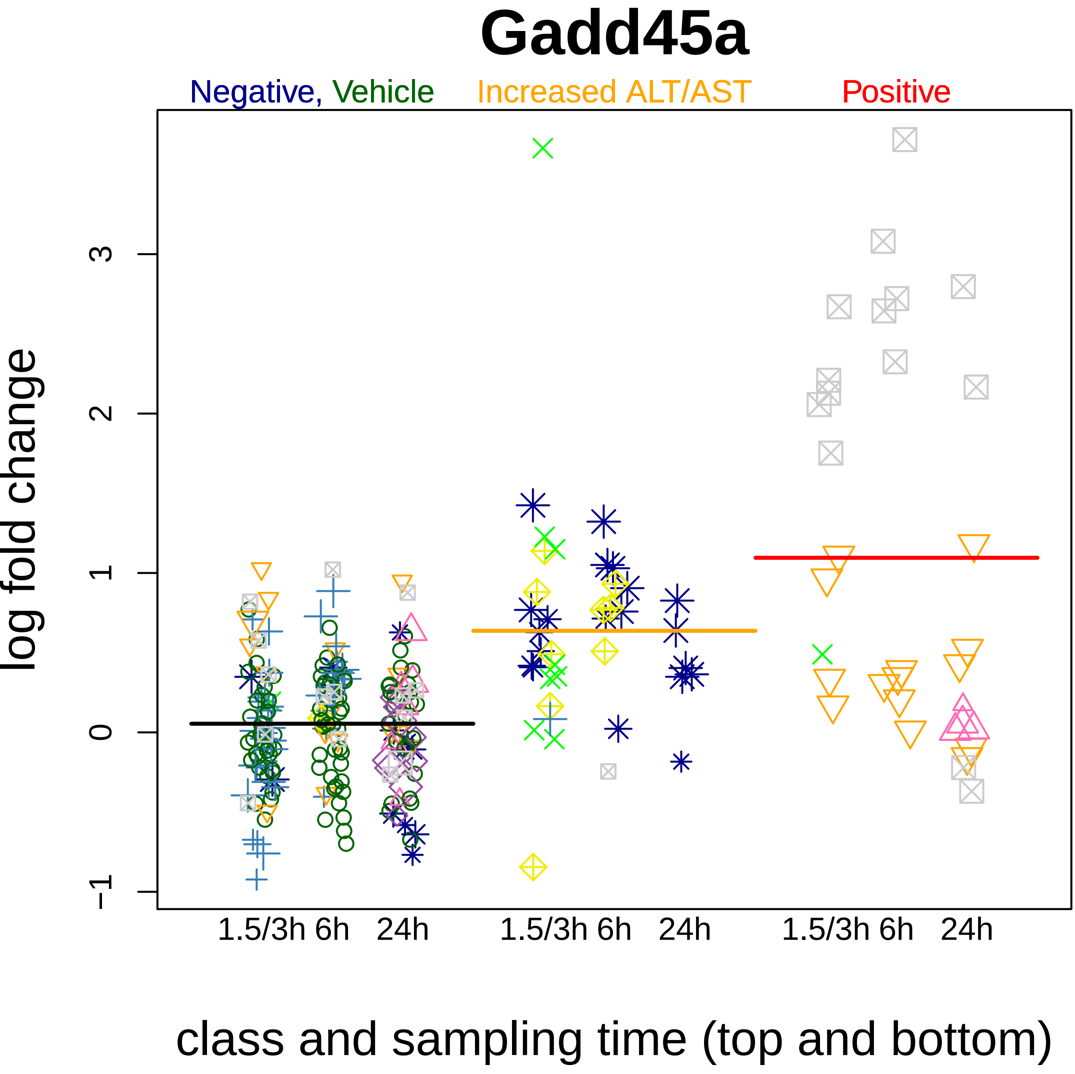
Gadd45a
Negative, Vehicle
Increased ALT/AST
Positive
log fold change
−1
0
1
2
3
1.5/3h 6h 24h
1.5/3h 6h 24h
1.5/3h 6h 24h
class and sampling time (top and bottom)

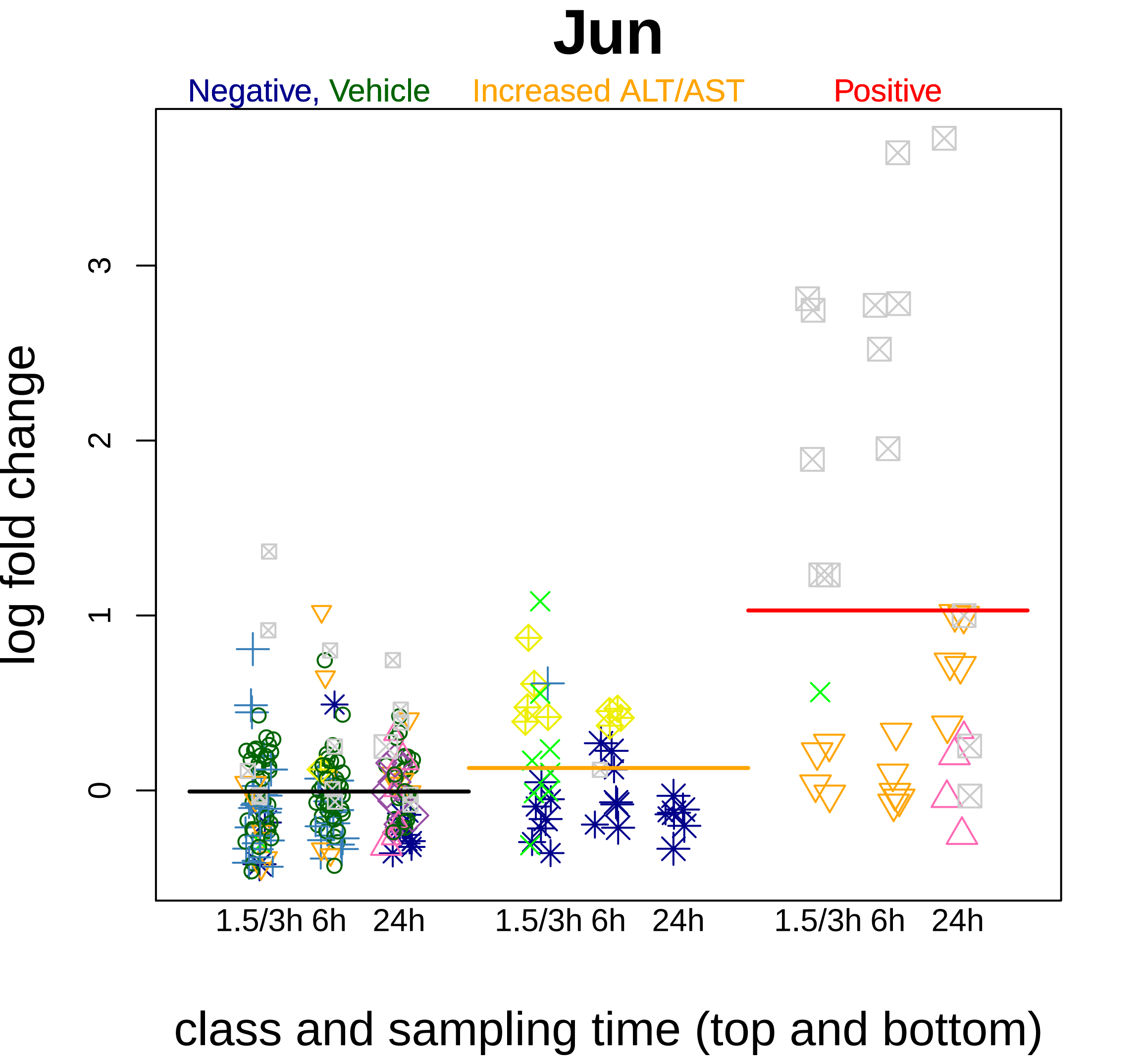
Jun
Negative, Vehicle
Increased ALT/AST
Positive
log fold change
0
1
2
3
1.5/3h 6h 24h
1.5/3h 6h 24h
1.5/3h 6h 24h
class and sampling time (top and bottom)

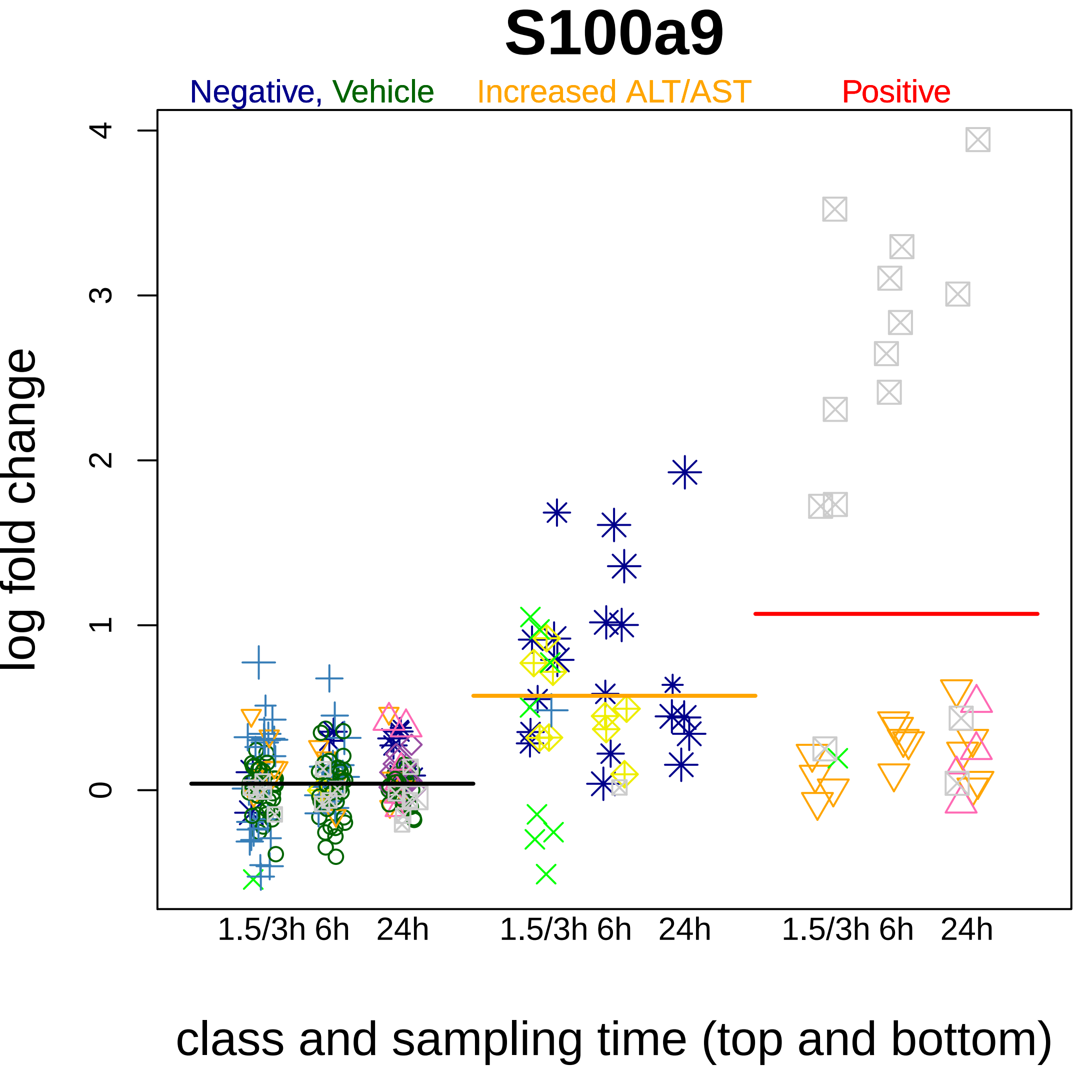
S100a9
Negative, Vehicle
Increased ALT/AST
Positive
log fold change
0
1
2
3
4
1.5/3h 6h 24h
1.5/3h 6h 24h
1.5/3h 6h 24h
class and sampling time (top and bottom)

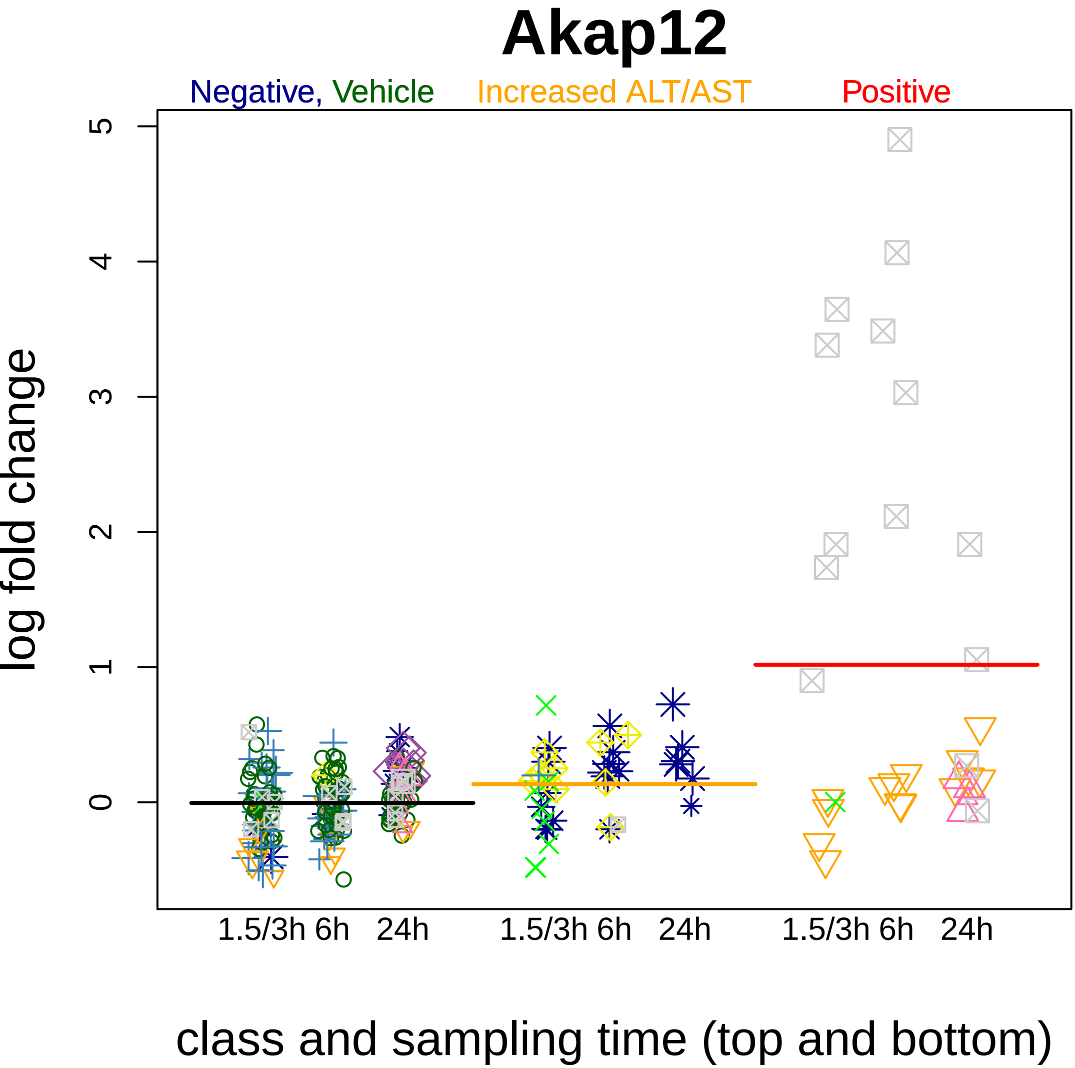

Akap12
Negative, Vehicle
Increased ALT/AST
Positive
log fold change
0
1
2
3
4
5
1.5/3h 6h 24h
1.5/3h 6h 24h
1.5/3h 6h 24h
class and sampling time (top and bottom)

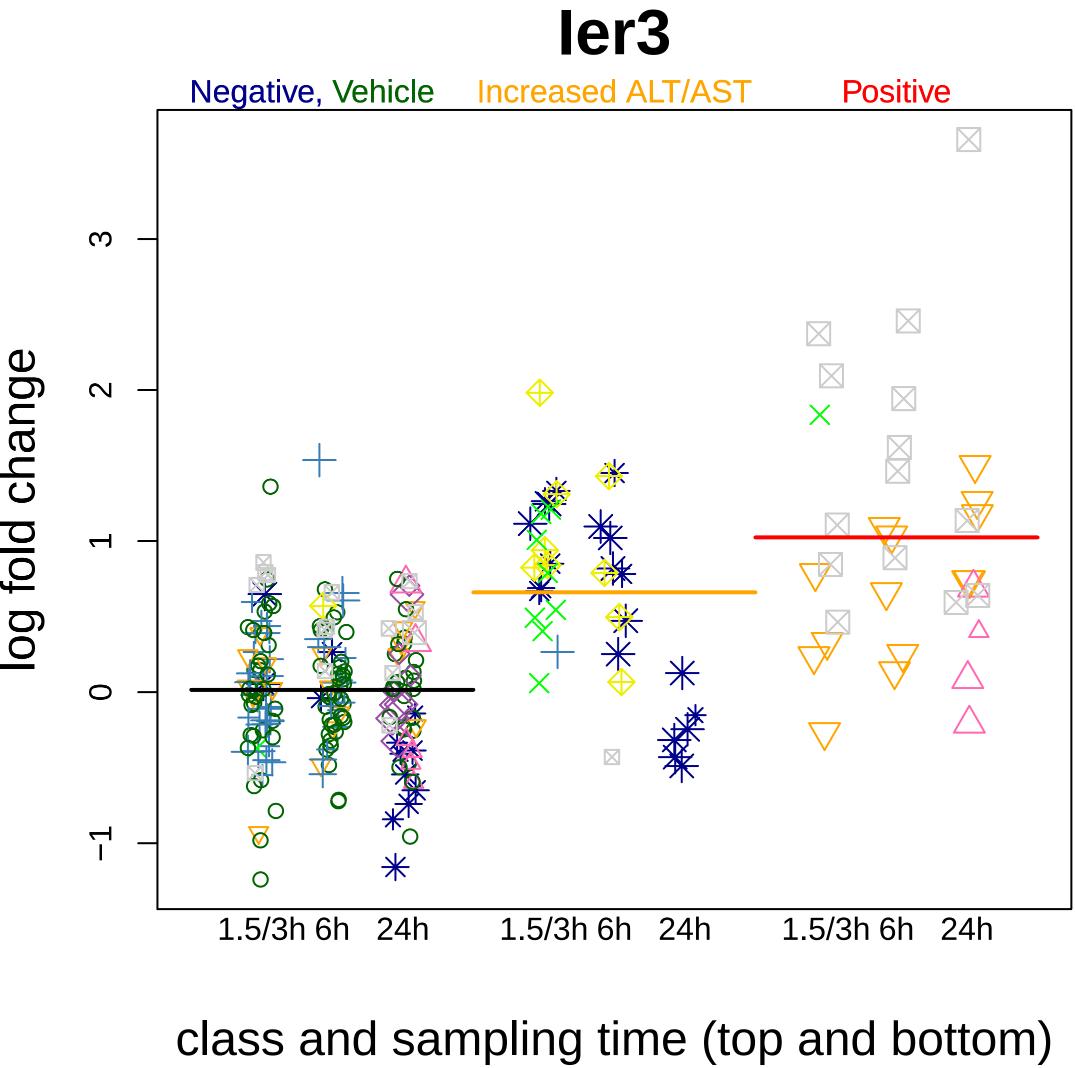
Ier3
Negative, Vehicle
Increased ALT/AST
Positive
log fold change
−1
0
1
2
3
1.5/3h 6h 24h
1.5/3h 6h 24h
1.5/3h 6h 24h
class and sampling time (top and bottom)

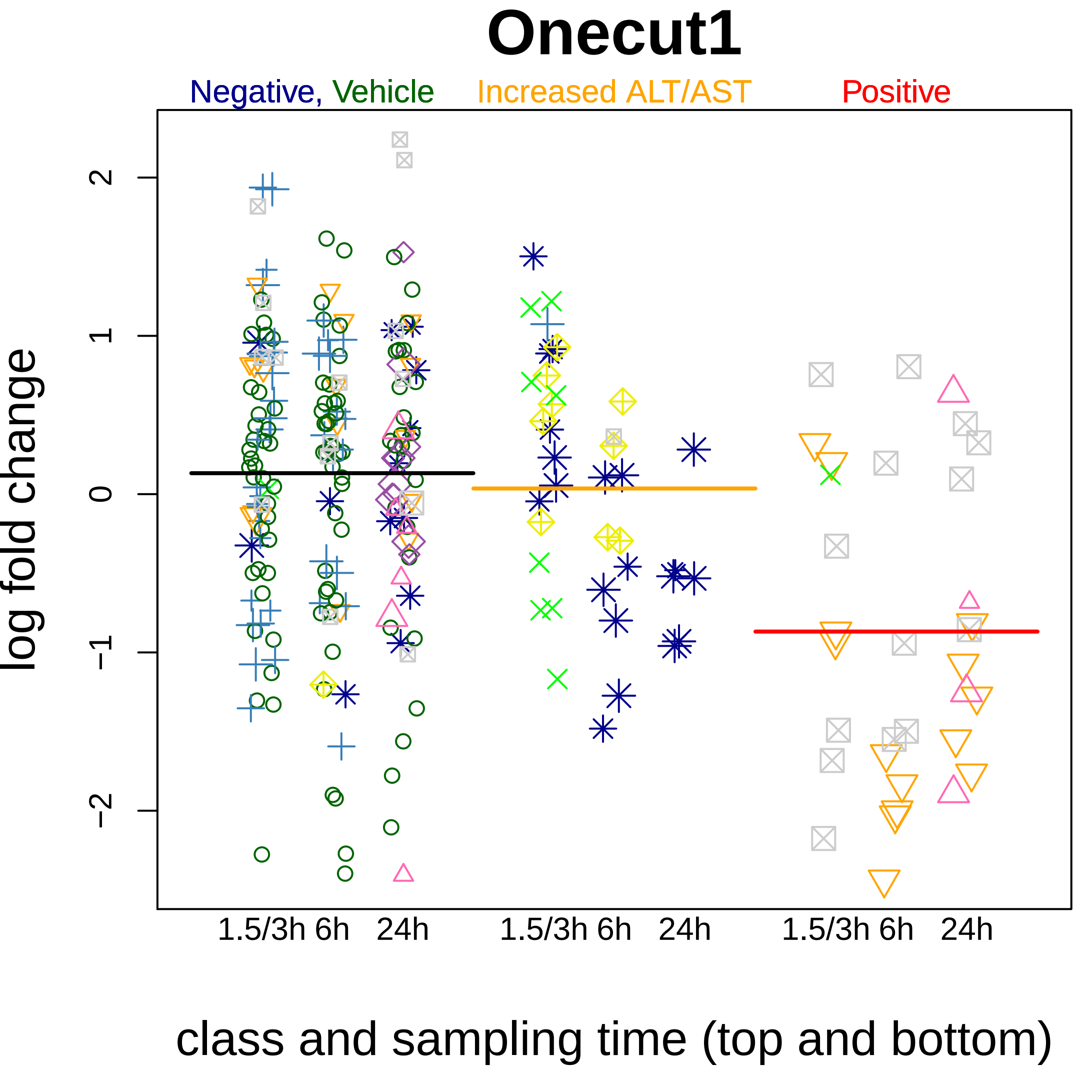
Onecut1
Negative, Vehicle
Increased ALT/AST
Positive
log fold change
2
1
0
−1
−2
1.5/3h 6h 24h
1.5/3h 6h 24h
1.5/3h 6h 24h
class and sampling time (top and bottom)
